# Analyzing the Solvent Effects in Palladium/*N*‑Heterocyclic Carbene (Pd/NHC)-Catalyzed Suzuki–Miyaura Coupling of Aryl Chlorides: A Computational Study of the Oxidative Addition Step with Experimental Validation

**DOI:** 10.1021/acs.jpcb.5c06092

**Published:** 2025-12-26

**Authors:** Max Collier, Brandon Rodriguez, Sean Lasiter, Addison D. Olmsted, Evan K. Simmons, Gregory R. Boyce, Daniel S. Lambrecht

**Affiliations:** † Department of Chemistry and Physics, 3391 Florida Gulf Coast University, 10501 FGCU Blvd. S., Fort Myers, Florida 33965, United States; ‡ Department of Chemistry and Biochemistry, 3087 East Stroudsburg University, 200 Prospect Street, East Stroudsburg, Pennsylvania 18301, United States

## Abstract

The impact of solvent effects on the oxidative addition step in the palladium/*N*-heterocyclic carbene (Pd/NHC)-catalyzed Suzuki–Miyaura coupling of aryl chlorides was studied using computational approaches and evaluated experimentally to determine which solvent properties are important when selecting a solvent for this catalyst system. Since oxidative addition is typically considered the rate-determining step of the cross-coupling with aryl chlorides, density functional theory (DFT) was employed to evaluate its energetics across 24 solvents spanning a broad range of physicochemical properties such as polarity, aromaticity, surface tension, and Abraham’s hydrogen bond acidity and basicity. The activation barrier and reaction energy were found to depend primarily on the polarity of the solvent with activation barriers ranging from 10.95 kcal/mol in the most polar solvent, water, to 12.35 kcal/mol in the least polar solvent, hexane. This corresponds to a 10.6-fold variation in rate constants at room temperature based on the solvent’s dielectric constant. The reaction energies were predicted to vary from −22.70 to −17.39 kcal/mol between the most and least polar solvents, respectively. The molecular origins for these findings arise predominantly from the charge transfer from the Pd(0) catalyst to the aryl chloride during the formation of the transition state for the oxidative addition step; however, this study investigated several additional solvent properties, such as the refractive index, hydrogen-bond acidity and basicity, surface tension, and aromaticity, that were not examined comprehensively in prior computational studies for this catalyst/substrate system. Some of these additional solvent properties were also found to impact the activation barriers. A series of solvents were then analyzed experimentally to determine if the yields corroborate the computational findings. These findings aid in developing a molecular understanding of the solvent properties’ effects on the oxidative addition in palladium/*N*-heterocyclic carbene (Pd/NHC)-catalyzed Suzuki–Miyaura coupling of aryl chlorides and help inform solvent selection for efficient performance of this important coupling reaction.

## Graphical abstract

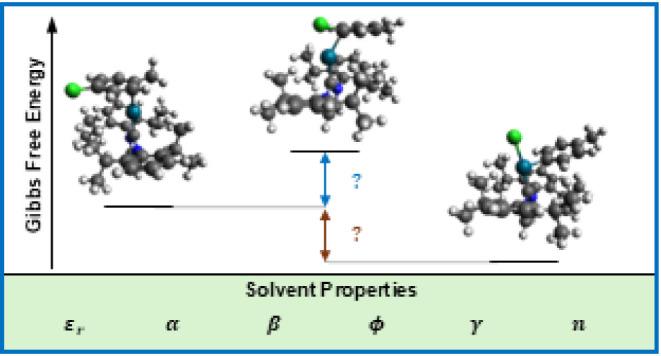

## 1 Introduction

The Suzuki–Miyaura reaction^1^ (Scheme 1) is a palladium-catalyzed carbon–carbon coupling reaction that is widely used in the synthesis of fine chemicals, pharmaceuticals, and functional materials.^2^ The reaction’s mild conditions, broad substrate scope, and generally high yields in a variety of solvents^3–5^ contribute to its significance in organic synthesis. While the cross-coupling is frequently performed in conventional organic solvents like acetonitrile, DMF, and THF, it can proceed in benign solvents like isopropanol and water.^2^ Numerous catalyst systems and precatalysts have been developed over the past decades employing a variety of ligand classes including phosphines^3–10^ and *N*-heterocyclic carbenes (NHCs)^11–14^ that further enhance the efficiency of the cross-coupling. Despite the considerable progress in the optimization of this reaction, side reactions such as protodeborylation, dehalogenation, and homocoupling can still occur demonstrating the need for a deeper understanding of the factors that determine reactivity and selectivity in the reaction. Consequently, the Suzuki–Miyaura coupling has been studied extensively mechanistically and computationally for many catalyst/substrate systems.^10,15–27^

**Scheme 1:**
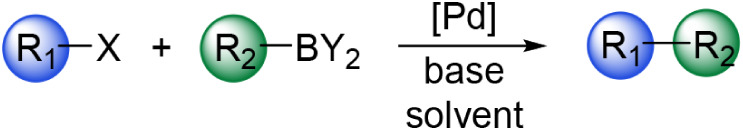
Suzuki–Miyaura Coupling Reaction

Solvent effects have a significant impact on the selectivity and reactivity of the Suzuki–Miyaura coupling as well as Pd-catalyzed cross-couplings in general.^28^ Practical considerations such as the need to solubilize polar boronic acids, nonpolar aryl halides as well as the metal catalyst and base can limit solvent selection in specific catalyst systems. Additionally, coordinating solvents can alter the identity of the active catalyst by competing with ligands and affect the outcome of the reaction. For instance, Neufeldt and coworkers determined that the chemoselectivity switch in the Suzuki coupling of chloroaryl triflates was likely due to coordinating solvents acting as ligands in the oxidative addition of the examined phosphine system.^29^ Moreover, solvent polarity has been found to impact the equilibrium between neutral and anionic Pd species as the active catalysts in reactions where anionic additives are present.^30^ This highlights the importance of solute–solvent interactions in Pd-catalyzed systems and the need to consider solvent effects specific to each catalyst/substrate system independently since they cannot be generalized across the diverse reaction conditions used in the Suzuki–Miyaura reaction. Furthermore, the specific reaction conditions of each catalyst system can confine the solvent choices based on the compatibility with the additives and bases employed leading to a variety of factors that need to be considered in identifying optimal solvents per catalyst system.

The complex effects of solvation in the Suzuki–Miyaura reaction prompted us to examine how the physicochemical properties of a range of solvents impact the oxidative addition elementary step in Nolan’s NHC/Pd catalyst system with aryl chlorides.^13^ The motivation for selecting this catalyst/substrate system was both fundamental and pragmatic. Nolan’s system is particularly useful as it allows for high-yielding reactions at room temperature even with challenging substrates like aryl chlorides. Additionally, the rate-determining step with aryl chlorides is the oxidative addition elementary step which differs from the more common aryl bromides and iodides where transmetalation is rate-determining.^19,21,24^ Finally, Nolan’s Pd/NHC catalyst system provides a well-established active catalyst structure that has been elucidated at atomistic detail for computational modeling.^13,27,31^ These features differ from other, more well-studied catalyst/substrate systems and led us to investigate which physicochemical solvent properties are important for this specific system.

To enable comparison with prior results, the NHC/Pd-catalyzed [Pd­(IPr)] (IPr = *N*,*N*’-bis­[2,6-(diisopropyl)­phenyl]­imidazole-2-ylidene) (Figure 1) coupling of *p*-chlorotoluene and phenylboronic acid to form 4-phenyltoluene (Scheme 2) that was previously studied by Meconi et al.^31^ was selected for the test reaction. Moreover, this study focused on the oxidative addition as the rate-determining step of the coupling reaction. While this selection neglects the importance of other stages of the reaction (e.g., the formation of the active catalyst species, cf. ref. 27), this limited scope enabled a more exhaustive study of the effects of a broad range of solvents on this important elementary step.

**Figure 1:**
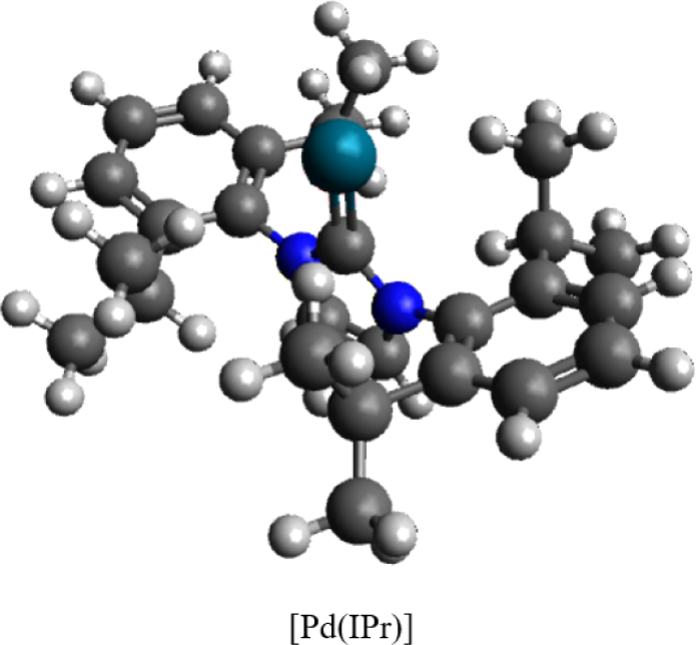
Structure of the active catalyst species [Pd­(IPr)] (IPr = *N*,*N*’-bis­[2,6-(diisopropyl)­phenyl]­imidazole-2-ylidene) studied in this work.

**Scheme 2:**
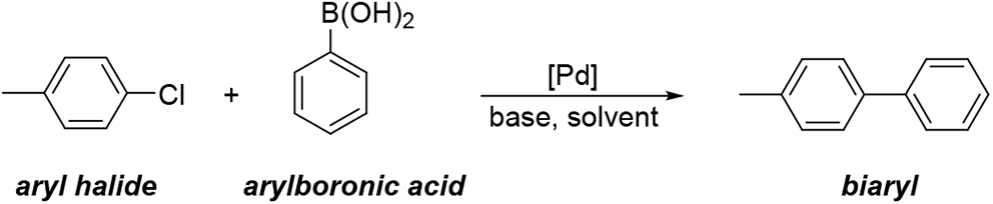
Test Suzuki–Miyaura Cross-Coupling Catalyzed by [Pd­(IPr)] Studied in This Work

The oxidative addition step of the coupling reaction begins with the formation of the first intermediate (**I1**) (Figure 2), a catalyst–substrate complex in which the active Pd species is loosely coordinated with carbon-1 (C1) of the substrate. The complex then proceeds to form the first transition state (**TS1**), where the Pd forms a partial bond to C1 and the chlorine (Cl) atom on the substrate, thereby partially weakening the C–Cl bond, as demonstrated by the elongation of this bond. The partial weakening of the C–Cl bond is key to enabling the Pd to be inserted into this bond to form intermediate 2 (**I2**), which enters the transmetalation step where the arylboronic acid adds ultimately enabling coupling of the two molecules.

**Figure 2:**
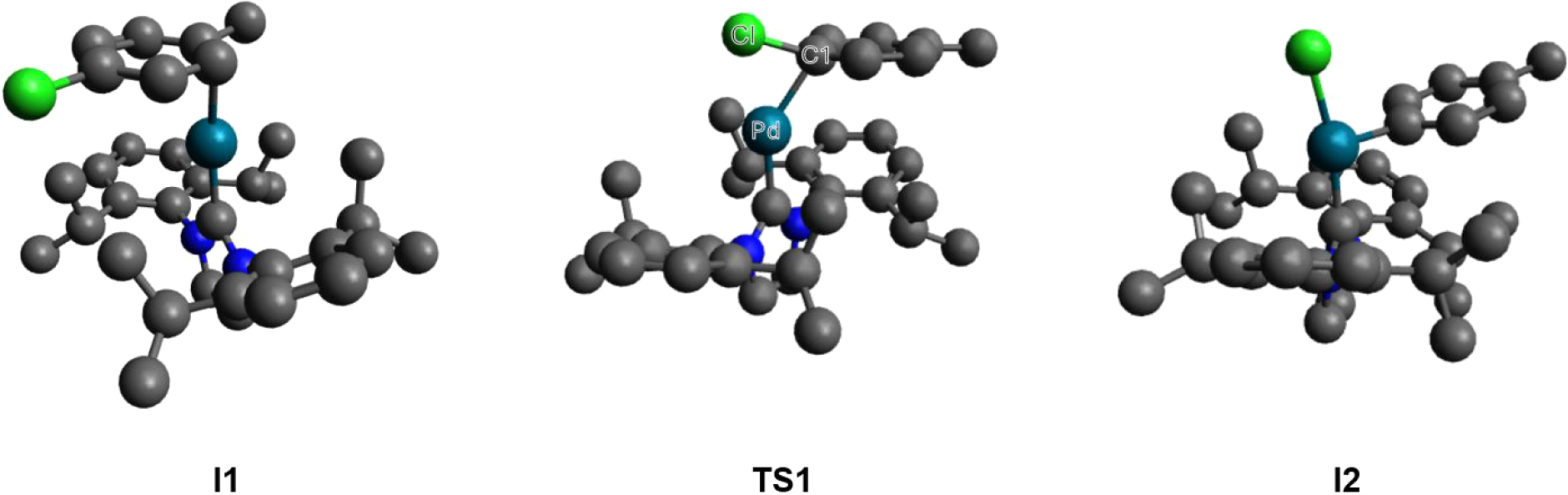
Structures of the first intermediate (**I1**), first transition state (**TS1**), and second intermediate (**I2**) of the oxidative addition as the rate-determining step of the coupling reaction. (Hydrogen atoms are removed for clarity.)

Focusing on this significant elementary step enables an in-depth study of solvent effects for this reaction. Twenty-four solvents were selected to span a range of physicochemical properties such as polarity, Abraham’s hydrogen bond acidity and basicity, and aromaticity (Table 1).^32–34^ Most of the solvents were selected from the CHEM21 database^35^ for being green, environmentally benign solvents. Conventional solvents were selected when there was no green alternative readily available for a conventional solvent with specific properties. Solvent impacts on the oxidative addition step were studied by investigating the energetics of intermediates **I1** and **I2** and transition state **TS1** when exposed to interactions with the different solvents. Moreover, the geometric and electronic structures of **I1**, **I2**, and **TS1** were investigated to develop a molecular understanding of how these would interact with solvents of different properties.

**Table 1:** Electronic and Gibbs Free Energies (in kcal/mol) for the Activation Energy (**I1** → **TS1**) and Reaction Energy (**I1** → **I2**) for the Oxidative Addition in Different Solvents Classified by Functional Groups and Dielectric Constants^a^

Solvent	ε*_r_* ^57^	Δ*E* ^‡^	Δ*E_rxn_*	Δ*G* ^‡^	Δ*G_rxn_*	*k*/*k*_0_
Water	80.1	12.93	–23.73	10.95	–22.70	2.045
DMSO	47.24	13.34	–23.32	11.34	–22.31	1.057
**Alcohols**						
Ethylene glycol	41.4	13.10	–23.45	11.10	–22.45	1.585
MeOH	33.0	13.35	–23.28	11.34	–22.30	1.069
EtOH	25.3	13.39	–23.14	11.37	–22.18	1.017
i-PrOH	20.18	13.41	–22.98	11.38	–22.04	1.000
*n*-BuOH	17.84	13.40	–22.91	11.36	–21.99	1.032
2-Methoxyethanol	17.2	13.31	–22.94	11.26	–22.03	1.216
*t*-BuOH	12.47	13.46	–22.60	11.41	–21.69	0.946
Isoamyl alcohol	15.63	13.45	–22.64	11.38	–21.75	0.984
2-Methyl-1-butanol	15.63	13.45	–22.63	11.39	–21.74	0.971
2-Pentanol	13.71	13.45	–22.53	11.38	–21.65	0.987
Benzyl alcohol	11.916	13.44	–22.58	11.37	–21.71	1.004
**Ketones**						
Acetone	21.01	13.51	–22.95	11.48	–22.02	0.843
MEK	18.56	13.54	–22.85	11.49	–21.92	0.818
MIBK	13.11	13.58	–22.56	11.51	–21.71	0.800
**Esters**						
Ethyl acetate	6.0814	13.68	–21.50	11.57	–20.80	0.721
PrOAc	5.62	13.70	–21.33	11.59	–20.64	0.697
*n*-BuOAc	5.07	13.72	–21.11	11.61	–20.45	0.677
**Ethers**						
THF	7.52	13.62	–21.87	11.51	–21.12	0.792
Anisole	4.30	13.82	–20.64	11.71	–20.04	0.570
**Hydrocarbons**						
Toluene	2.379	14.07	–18.67	12.04	–18.26	0.324
Cyclohexane	2.0243	14.25	–17.95	12.32	–17.60	0.203
Heptane	1.9209	14.27	–17.70	12.35	–17.39	0.193

a ωB97X-D3/def2-TZVPP/SMD//PBE-D3­(BJ)/def2-SVP/C-PCM level of theory.

## 2 Computational Approach

The test reaction was studied at the ωB97X-D3/def2-TZVPP//PBE-D3­(BJ)/def2-SVP level of theory.^36–45^ This choice of methodology follows computational workflows based on extensive benchmarks and previously published model studies.^46–48^ Initial geometries were adopted from the structures published by Meconi et al.^31^ followed by geometry relaxation at the PBE-D3­(BJ)/def2-SVP level of theory. Single-point calculations were performed at the ωB97X-D3/def2-TZVPP level of theory at these optimized geometries. Solvent effects were included using the Conductor-like Polarizable Continuum Model (C-PCM)^49,50^ within the geometry optimizations and the Solvation Model based on Density (SMD)^51^ for single-point calculations. SMD parameters were not available for the solvent i-PrOAc; therefore, the parameters for *n*-PrOAc were used. For the same reason, the solvents isoamyl alcohol, 2-methyl-1-butanol, and 2-pentanol were treated entirely at the C-PCM level shifted by a correction to adjust the energy scale to that of the SMD-based results (Figure S1). The def2-ecp scalar relativistic effective core potential was employed for palladium.^52^ Calculations were performed using the ORCA quantum chemistry program package.^53–56^ It was found that the transition state optimizations were highly sensitive to the initial geometry, the tightness of the numerical thresholds, and optimization settings such as the frequency of exact Hessian evaluation (cf. Table S2 and Figure S1). Therefore, the TightSCF and TightOpt settings were used along with exact Hessian reevaluations for every 10 optimization steps; especially, the Hessian reevaluation was found to be critical to obtain reliable and reproducible transition state structures. Thermodynamic corrections were calculated using the standard approaches implemented in ORCA. However, the calculation of vibrational entropies required some modifications, as outlined in the (Supporting Information pp. S6–S11).

## 3 Results and Discussion

The Gibbs free energies of activation or activation energy (Δ*G* ^‡^) for the activation step **I1** → **TS1** and the free energy of reaction Δ*G_rxn_* for the reaction **I1** → **I2** were predicted using density functional theory as outlined in the computational approach section. Computed numbers are shown in Table 1 and visualized in Figures 3 and 4. Electronic energies are also reported to enable assessment of purely electronic effects. Trends in activation energies and reaction energies were analyzed with respect to solvent properties as described by the solvent physicochemical descriptors as well as grouped by the chemical classification of the solvents based on functional groups.

**Figure 3:**
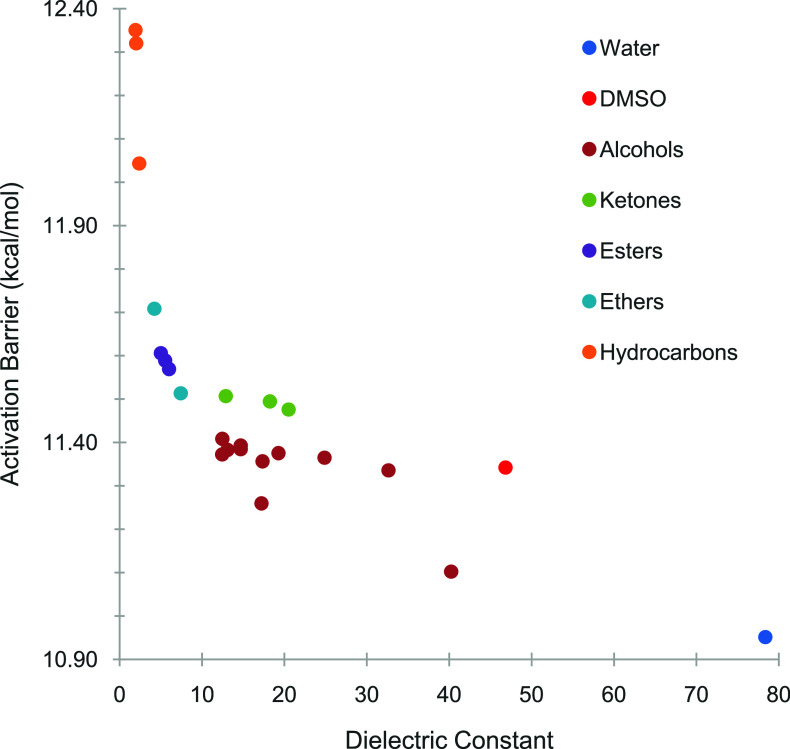
Gibbs free energies (in kcal/mol) for the activation energy (**I1** → **TS1**) for the oxidative addition in different solvents classified by functional group.

**Figure 4:**
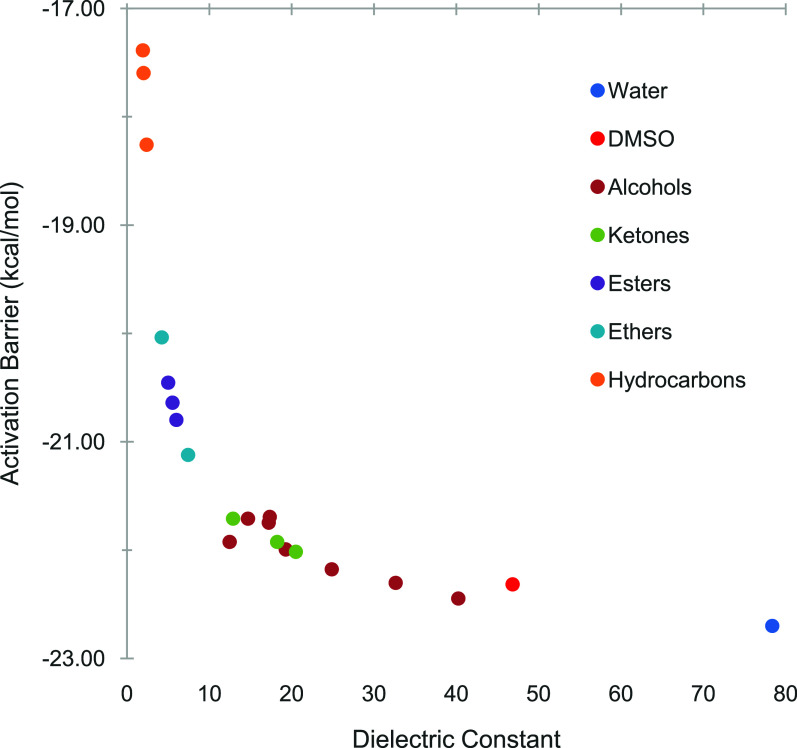
Gibbs free energies (in kcal/mol) for the reaction energy (**I1** → **I2**) for the oxidative addition in different solvents classified by functional group.

### 3.1 Solvent Effect on Free Energy of Activation

For the solvents investigated in this study, the free energy of activation Δ*G* ^‡^ was predicted to range between 10.95 and 12.35 kcal/mol depending on the solvent (Table 1). Overall, a negative correlation between the activation barrier and the solvent polarity was found, where the lowest barrier was predicted for water as the most polar solvent tested and the highest barrier was predicted for heptane as the least polar solvent tested. Plotting the dependence of the activation barrier on the solvent dielectric constant results in a hyperbolic curve with minor outliers from this trend (Figure 3). Trends in the electronic contributions to the activation barrier are identical, suggesting that the observed trends stem primarily from the electronic energy. The variation in activation barriers can be put into perspective by calculating the relative rate constants in different solvents. The activation barrier and rate constant for isopropanol were used as a baseline since this solvent has been previously reported to work well for the test reaction.^13^ At a calculated barrier of 11.38 kcal/mol, the rate constant in isopropanol is calculated via Eyring’s equation as $k_{0}=\frac{k_{B}T}{h}e^{-\Delta G^{‡}/RT}=2.85\times 10^{4}s^{-1}$ . Relative to this, the variation in activation barrier between different solvents corresponds to a range of relative rate constants from *k*/*k*_0_ = 0.193 for heptane to *k*/*k*_0_ = 2.045 for water (Table 1). In other words, the relative rate of the studied solvents can vary by a magnitude of 10.6×. The finding that the solvent impacts the relative rate constant by over an order of magnitude speaks to the significance of solvent effects on the oxidative addition step.

An interesting observation is that the activation barrier varies rapidly over a range of approximately 12.4–11.4 kcal/mol for solvent dielectric constants between ε ≈ 1 and 12, whereas it remains relatively constantly low at a range of approximately 11.4–10.9 kcal/mol for dielectric constants of ε ≈ 12–80. In other words, there is a large range of solvent polarities over which the activation barrier for the oxidative addition reaction remains relatively low. This finding could be one explanation why the Suzuki–Miyaura reaction works well in a broad range of solvents.

Results were also analyzed by grouping solvents according to their chemical functionality. This classification can be found in the subheadings within Table 1 as well as the color-coding in Figure 3. Clearly, solvents within the different functional groups show activation barriers that are clustered together, with hydrocarbons leading to the highest barriers, followed by ethers and esters, then ketones, alcohols, and finally water with the lowest barrier. These trends roughly follow the observation discussed above that the trends in activation barriers overall follow the trends in polarity of the solvents. However, it is also noted that there is some scattering in the trends when the activation barrier is plotted against the dielectric constant, as observed for example between ketones and alcohols. This difference suggests that while the trends in the activation barriers are dominated by the solvent polarity, other solvent properties also need to be considered. This finding warranted a more in-depth investigation of the interdependency between the solvent properties and activation barriers, as well as the molecular origins for the observed trends.

### 3.2 Solvent Effect on Reaction Energy

The reaction free energies Δ*G_rxn_* were predicted using density functional theory with the results displayed in Table 1 and visualized in Figure 4. Reaction energies range between −22.70 kcal/mol for water as the most polar solvent and −17.39 kcal/mol for heptane as the least polar solvent, a 5.31 kcal/mol range. Overall, the predicted trends are similar to those reported for the activation barriers in that plotting the reaction energy versus the solvent dielectric constant results in a curve with an overall hyperbolic shape (Figure 4). Also, the classification by functional group results in similar results as the analysis of the activation barrier, namely that reaction energies become more favorable (more exergonic) in the order from hydrocarbons, ethers and esters, ketones and alcohols, and finally water. As observed above, trends in the electronic contributions to the reaction energies are identical to those for the free energies, suggesting that the observed trends stem primarily from the electronic energy. Fewer outliers are observed in the reaction energy versus dielectric constant trend compared to the activation barrier.

### 3.3 Solvent Physicochemical Properties Impact

To better understand the role that the different physicochemical solvent properties play in impacting the energetics of the oxidative addition reaction, we analyzed the statistical covariance between solvent descriptors and the free energies of activation and reaction, respectively. Since covariance is a measure for the joint variation between a pair of variables, it is a measure for how much the free energies vary with the solvent parameters. Included in this analysis were the solvent properties incorporated in the SMD solvation model, that is, the relative dielectric constant ε, index of refraction at optical frequencies *n*, Abraham’s hydrogen bond acidity α, Abraham’s hydrogen bond basicity β, surface tension γ, and aromaticity ϕ. Halogenicity was not considered since none of the tested solvents contained halogens.

For both the activation and the reaction energy, the strongest covariance was found with the relative dielectric constant ε (Table 2). At covariances of −3.863 and −15.849, respectively, the activation energy and reaction energy decrease strongly with the dielectric constant. This finding agrees well with the results from plotting the activation/reaction energy versus the dielectric constant, which suggested a strong dependence of these energies on the solvent polarity. The covariances for all other solvent parameters are substantially smaller, with the surface tension γ showing the next-largest values of −0.083 and −0.830 for the activation barrier and reaction energy, respectively, followed by Abraham’s hydrogen bond basicity and Abraham’s hydrogen bond acidity at covariances of −0.053 and −0.033 for activation barriers as well as −0.269 and −0.150 for reaction energies. This analysis confirms the dominant role of solvent polarity as a predictor of activation barriers and reaction energies. It should be noted, however, that a small covariance does not necessarily imply that there is no connection between the variable pair, but rather that there is no linear relationship. We analyzed the data by plotting the energies versus all of the solvent parameters and found mostly random patterns for all parameters other than the dielectric constant and Abraham's hydrogen bond basicity, confirming that there is no clear correlation between energetics and surface tension, Abraham’s hydrogen bond acidity, index of refraction at optical frequencies, or aromaticity.

**Table 2:** Covariance Analysis between Solvent Physicochemical Descriptor and the Predicted Free Energy of Activation and Reaction Energy, Respectively

	ε	*n*	α	β	γ	ϕ
Δ*G* ^‡^	–3.863	0.005	–0.033	–0.053	0.083	0.022
Δ*G*_*rxn*_	–15.849	0.030	–0.150	–0.269	–0.830	0.112

The special role of the solvent polarity and its impact on the activation barrier was further confirmed by designing a simple predictive model that correlates solvent properties with the activation barrier. In this model, the activation barrier is estimated via the equation:

1 $${\Delta G}_{\mathrm{Model}}^{‡}=c_{0}+c_{\varepsilon }\cdot \varepsilon^{-1}+c_{n}\cdot n+c_{\alpha }\cdot \alpha +c_{\beta }\cdot \beta +c_{\gamma }\cdot \gamma +c_{\phi }\cdot \phi$$

where the coefficients *c*_*x*_ are relative weights for property *x* with *c*_0_ being an additive constant. For the dielectric constant, an inverse relationship was assumed based on the rationale that Coulomb interactions depend on the inverse of the dielectric constant. Several simple expressions were tested for the other solvent descriptors, including linear and quadratic terms, but simple linear expressions were found to work equally well. The parameters in eq 1 were optimized to minimize the quadratic error in predicted activation barriers compared to the density functional theory predictions (Table 3). The optimized model reproduced the DFT activation barriers with a root-mean-square deviation (RMSD) of 0.085 kcal/mol and plotting the model versus the DFT reference resulted in a regression coefficient of *R* ^2^ = 0.929 (Figure 5), demonstrating that the model incorporates the most important correlations. Successively removing individual solvent descriptors from the model and reoptimizing the constants *c*_*x*_ enabled an alternative assessment of the relative significance of the different solvent parameters (Table 3). Removing the dielectric constant resulted in a deterioration of the model to an RMSD of 0.191 kcal/mol, and the regression coefficient relative to the DFT reference dropped to *R* ^2^ = 0.641. In contrast, the removal of any of the other solvent parameters from the model had no significant impact on the RMSD (0.085–0.090 kcal/mol) or the correlation coefficient (*R* ^2^ = 0.920–0.929). In fact, a minimal predictive model utilizing only the dielectric constant

2 $${\Delta G}_{\mathrm{Model}}^{‡}=c_{0}+c_{\varepsilon }\cdot \varepsilon^{-1}$$

was found to reproduce the trends in the activation barriers as predicted by DFT as a reference with an RMSD of 0.094 kcal/mol and a linear regression correlation coefficient of *R* ^2^ = 0.913 (Figure 6). This finding further confirms that the trends in activation barriers are dominated by the solvent polarity and that solvent polarity is the most important predictor for the activation barrier, with the overall significance of the solvent parameters described by the relation ε ≫ β > *n* ≈ α > γ ≈ ϕ.

**Table 3:** Performance of Predictive Models Based on Physicochemical Solvent Properties^a^

*c*_0_	*c*_ε_	*c*_*n*_	*c*_α_	*c*_β_	*c*_γ_	*c*_ϕ_	RMSD	*R* ^2^
11.2189	2.4163	–0.0742	–0.1322	0.3330	–0.0018	0.0267	0.085	0.929
11.3880	-	0.1479	–0.4884	–1.2077	–0.0151	–0.1597	0.191	0.641
11.1896	2.3026	-	–0.1453	0.2592	–0.0023	0.0146	0.088	0.925
11.2133	2.5001	–0.0802	-	0.3458	–0.0025	0.0411	0.087	0.925
11.2444	2.0698	–0.0417	–0.1446	-	0.0018	–0.0235	0.090	0.920
11.2279	2.3053	–0.0798	–0.1509	0.2267	-	0.0044	0.086	0.928
11.2197	2.3920	–0.0711	–0.1402	0.3040	–0.0014	-	0.085	0.929

a The models have the general form $\Delta G_{\mathrm{Model}}^{‡}=c_{0}+c_{\varepsilon }\cdot \varepsilon^{-1}+c_{n}\cdot n+c_{\alpha }\cdot \alpha +c_{\beta }\cdot \beta +c_{\gamma }\cdot \gamma +c_{\phi }\cdot \phi$ ,where ε, *n*, α, β, γ, and ϕ are the solvent dielectric constant, refractivity, acidity, basicity, surface tension, and aromaticity, respectively. The constants *c*_*x*_ are the relative weights for property x with *c*_0_ being an additive constant. The impact of the different solvent properties was tested by selectively removing individual properties from the model, which is indicated by a dash in the table. The root mean square deviation (RMSD) for the model is reported relative to the density functional theory results as a reference (in kcal/mol). The regression coefficient *R* ^2^ is reported for a linear regression analysis of the model predictions versus the DFT results as a reference.

**Figure 5:**
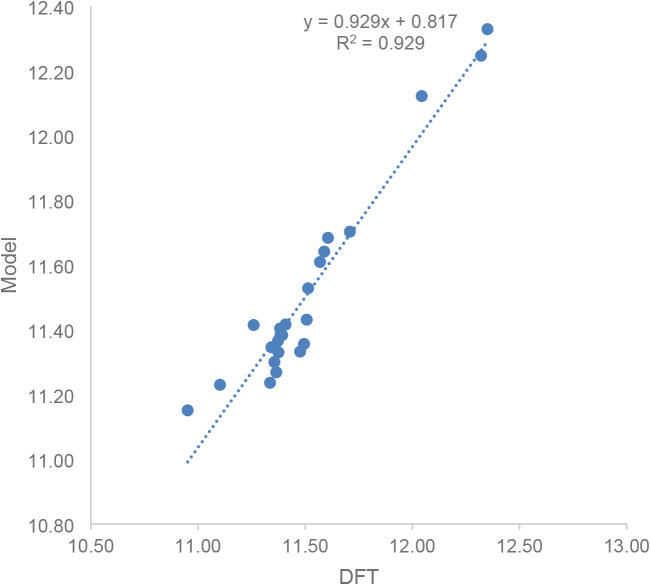
A comparison of the predictive model based on the full set of six physicochemical solvent properties considered in this study.

**Figure 6:**
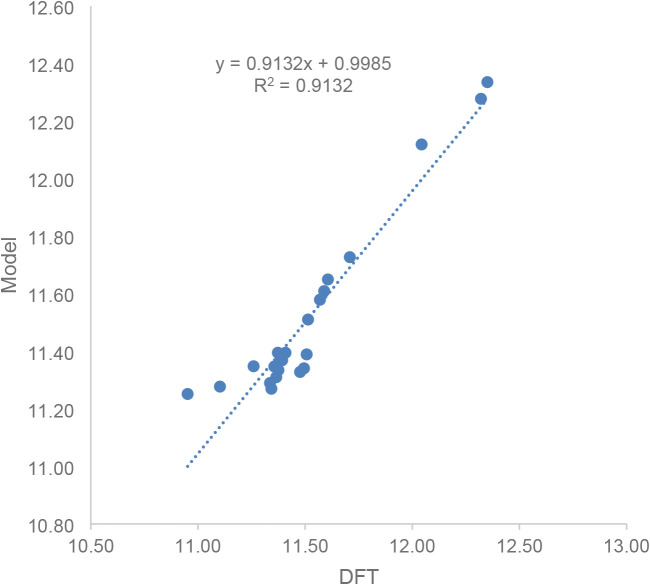
Activation barriers predicted with the two-parameter model compared to density functional theory for the reference.

While this analysis revealed the dominant trend in the activation barriers as solvent properties are considered, the question remains how the scattering of the data around the overall ε^–1^ trend can be explained. Clearly, the scatter around the overall hyperbolic dependence of the activation barriers on the value of the dielectric constant is small, but investigating this finding could add further insights into how solvents impact the activation barrier of the oxidative addition step. To address this question, the activation barriers were plotted against the dielectric constant with the data partitioned into subsets according to the magnitudes of secondary solvent properties and color-coded according to these subsets. An interesting pattern arose when the solvent Abraham’s hydrogen bond acidity α was chosen as a secondary solvent property and the data was partitioned according to smaller and higher solvent Abraham’s hydrogen bond acidity (Figure 7). This analysis revealed that the activation barriers versus dielectric constant data are bifurcated into one curve showing slightly higher overall barriers for small solvent Abraham’s hydrogen bond acidity (α < 0.1) and a branch showing slightly lower barriers for higher solvent Abraham’s hydrogen bond acidity (α = 0.3–0.6). Another interesting correlation was observed between the activation energies versus the solvent basicity (Figure 8), which shows a clear negative linear correlation. While these effects are less pronounced than the overall trends observed with respect to the dielectric constant (Δ*G* ^‡^ ∝ ε^–1^), they demonstrate that secondary solvent properties such as solvent acidity and basicity also impact the overall trends in the activation barrier. These effects were only investigated for the activation barriers, since the dependence of the reaction energies on the dielectric constant is even more pronounced and other solvent parameters have an even smaller effect on the reaction energies.

**Figure 7:**
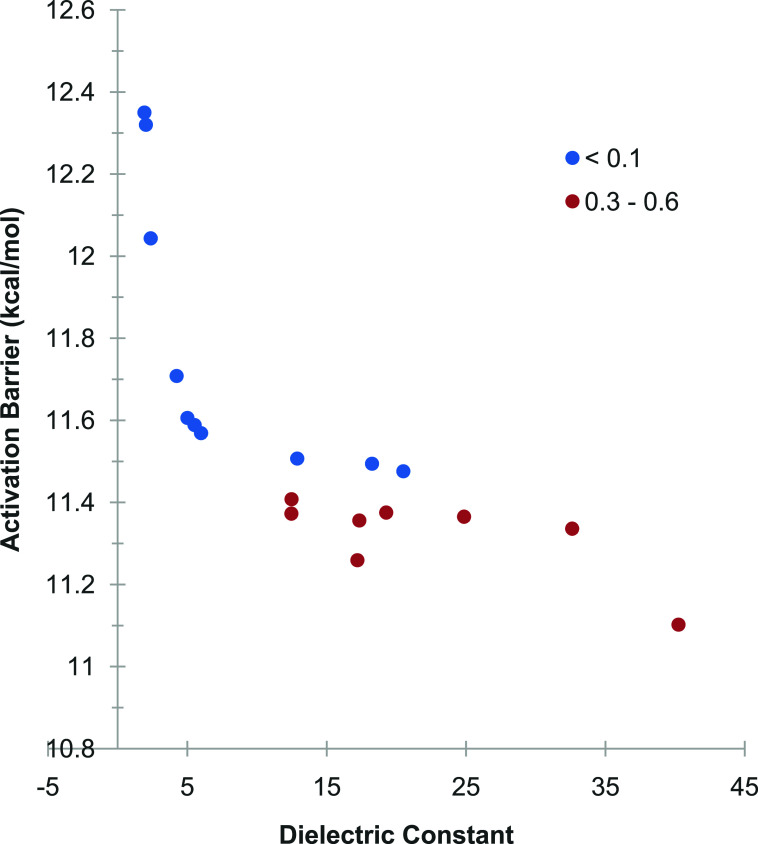
Activation barriers plotted versus the dielectric constant of the solvent, where the data is split into two subsets—one for small solvent acidity (α < 0.1) and the remaining solvents (α = 0.3–0.6). Solvents without acidities listed in the SMD solvent database were omitted.

**Figure 8:**
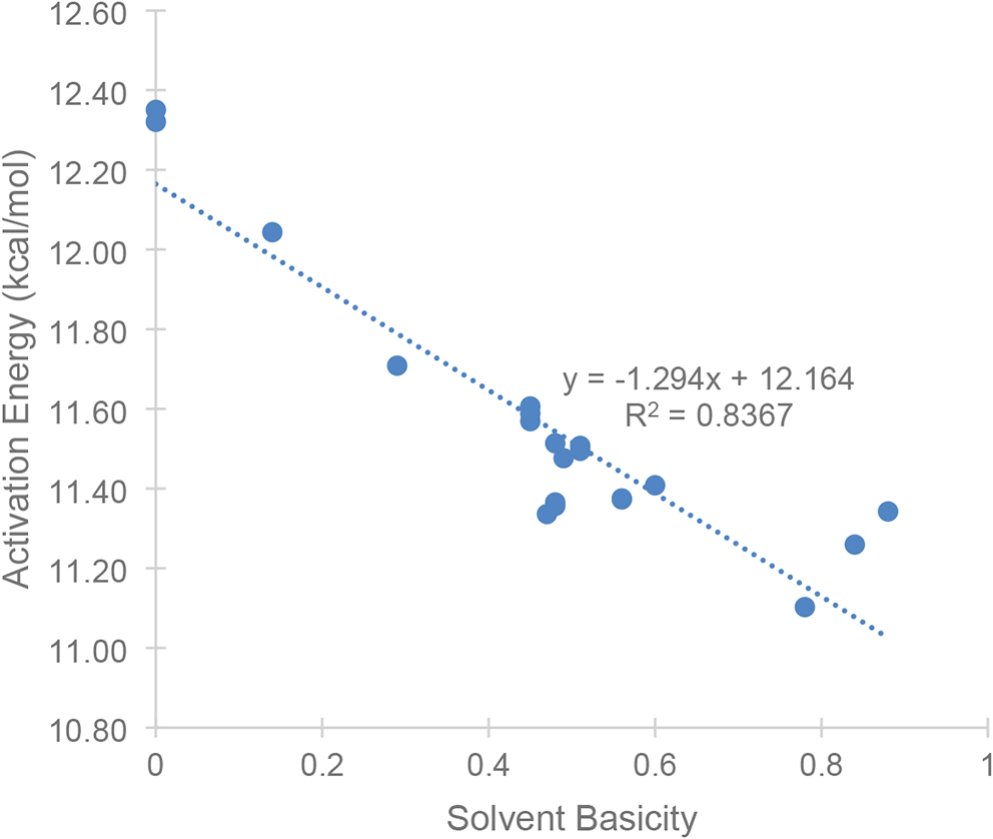
Activation barriers plotted versus the solvent basicity β. Solvents without basicities listed in the SMD solvent database were omitted.

### 3.4 Molecular Origins of Predicted Trends in Activation Barriers

The strong reciprocal relationship between the activation/reaction energy and the solvent dielectric constant suggests that electrostatic interactions between the solute and solvent play an important role in explaining the predicted effects. To investigate the molecular origins of these interactions, the overall polarities of the different species (**I1**, **TS1**, and **I2**) were investigated (Table 4). The overall polarity of the intermediates increases in the order **I1** < **TS1** < **I2**, with **TS1** having a 0.96 D (16.5%) higher dipole moment and **I2** having a 7.93 D (236%) higher dipole moment compared to **I1**. These differences in solute polarity suggest that the intermediates experience different levels of electrostatic (dipole–dipole) stabilization in polar solvents, with **I1** experiencing the lowest stabilization as the least polar solute, **TS1** experiencing medium stabilization, and **I2** experiencing the strongest stabilization as the most polar solute. This ranking based on solute polarities also explains why the activation barriers show a moderate 1.40 kcal/mol lowering versus the reaction energies experiencing a stronger 5.31 kcal/mol lowering between the least and most polar solvents.

**Table 4:** Dipole Moments of the Reactants **I1**, Transition State **TS1**, and Intermediate **I2** Calculated at the PBE-D3­(BJ)/def2-SVP Level of Theory within the C-PCM Model for Isopropanol as the Solvent

Structure	Dipole (Debye)
**I1**	5.83
**TS1**	6.79
**I2**	13.76

To rationalize the differences in polarities between intermediates **I1**, **TS1**, and **I2**, we considered the change in geometry as **I1** rearranges into **TS1** and then finally into **I2** (Figure 2) as well as how charges are redistributed during this process (Figure 9). First, it is noted that the atoms most involved in the oxidative addition reaction are the palladium atom of the catalyst and the chlorine and carbon atoms on the substrate, labeled Pd, Cl, and C1 in Figure 9. During the course of the oxidative addition reaction, the Pd approaches the carbon-1 (C1) and chlorine (Cl) atoms and ultimately inserts itself into the C1–Cl bond to form a new C1–Pd–Cl bond. During this process, charge is redistributed between these three atoms leading to an overall higher charge separation, which is consistent with the notion that the step corresponds to an oxidation of the palladium. Within **I1**, the charge on Pd is −0.64 e, whereas C1 is essentially neutral with a charge of 0.01 e, and Cl is slightly negative with a charge of −0.11 e. As the transition state **TS1** forms, a partial bond is formed between the Pd atom and C1 and between Pd and Cl; at the same time, the palladium loses part of its charge to −0.45 e, while C1 becomes partially negative at −0.21 e, and Cl becomes close to neutral at a charge of −0.01 e. As intermediate **I2** forms, a full bond of type C1–Pd–Cl is formed, with palladium losing even more of its charge as it becomes −0.32 e. Cl is the ultimate recipient of the charge and becomes −0.31 e as well, while C1 returns to its neutral (0.00 e) atomic charge. In essence, this process amounts to a transfer of electron density from the [Pd­(IPr)] catalyst onto the substrate. Due to the overall neutrality of the complex, the IPr ligand carries an overall positive partial charge, and the transfer from Pd onto C1 (in **TS1**) and onto Cl (in **I2**) means an increase of charge separation. As a result, the overall dipole moment increases in the order **I1** < **TS1** < **I2** due to the increasing degree of charge separation, thus leading to stronger dipole–dipole interactions between the more polar solutes and solvents. Overall, this study provides a simple molecular explanation for the dependence of the activation and reaction energies on the solvent polarity based on considering the charge transfer occurring during the oxidative addition reaction.

**Figure 9:**
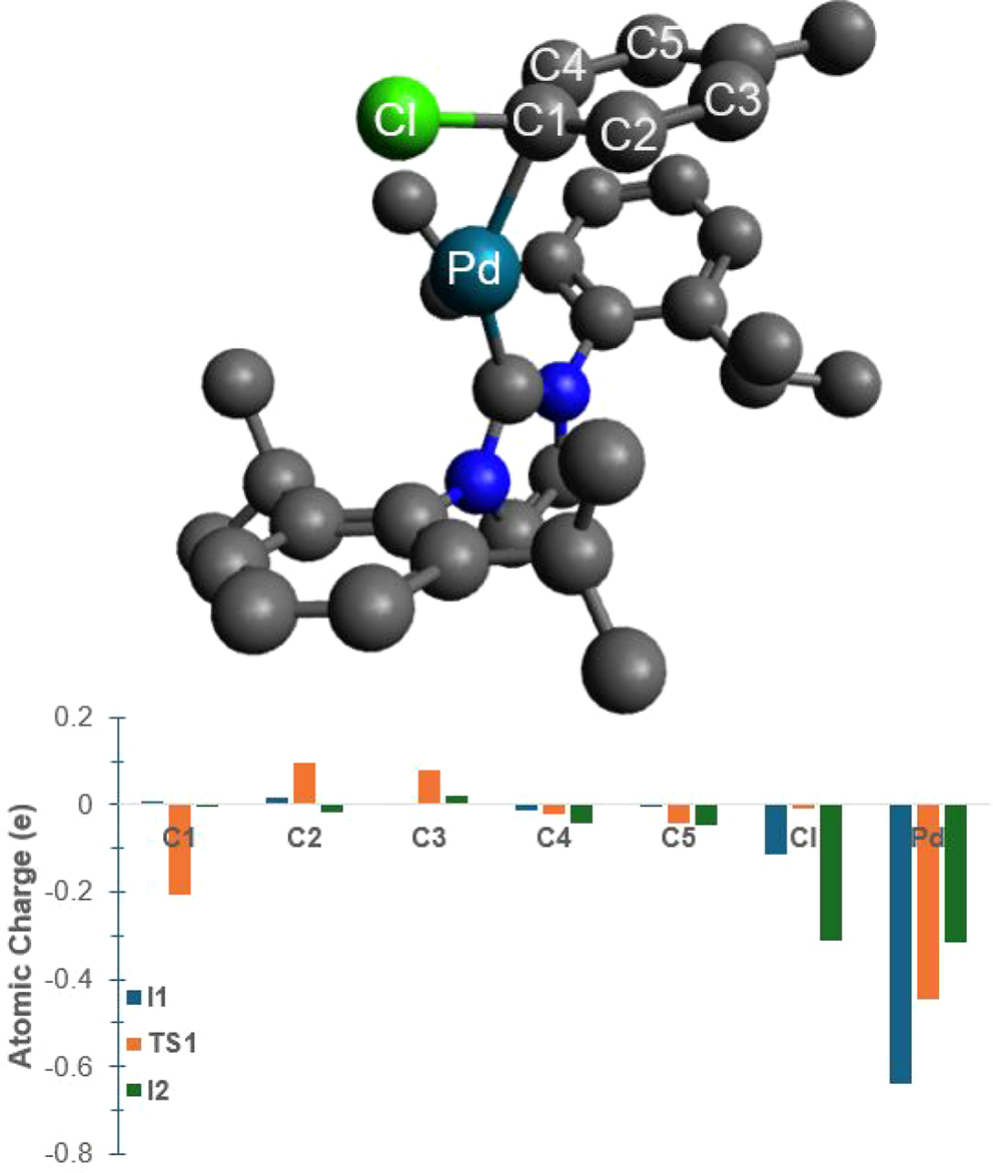
Atomic charges of selected atoms involved in the formation of intermediate 1, the transition state, and intermediate 2. For reference, the structure of the transition state (**TS1**) is shown.

## 4 Experimental Studies

The coupling of 4-chlorotoluene and 4-phenylboronic acid catalyzed by (IPr)­Pd­(cin)Cl (cin = cinnamyl) was performed in the computationally investigated solvents to determine the correlation between the computational findings and the experimental yield of the reaction. It is important to note that some variation in yield can be attributed to other factors including side reactions like homocoupling, catalyst decomposition, and a variety of parameters that can affect the outcome of the reaction including precatalyst loading, reaction stoichiometries, time, and temperature. Several of the computationally investigated solvents were disqualified from the experimental study either due to the insolubility of one or more of the reagents or incompatibility with the alkoxide reaction conditions (i.e., acetic acid, nitromethane, nitriles, ketones, esters, amides) specific to the test reaction that would lead to neutralization of the alkoxide, aldol reactivity, or saponification. This constrained the scope of the experimental study to alcohol-based solvents and a few aprotic examples (see Supporting Information for the full list of attempted solvents). The reaction conditions, temperature, and time were standardized based on the originally reported procedure^13^ with the exception that KOH was used as the base instead of KOtBu in primary and secondary alcohol solvents.

The test reaction generally proceeded well in primary and secondary alcohol solvents (Table 5). Aside from methanol (entry 1), which provided the lowest yield of any of the nontertiary alcohol solvents, the other primary alcohol solvents (entries 2–7) provided higher yields than the secondary alcohol solvents (entries 8–10). Water, which was determined to have the lowest activation barrier computationally, was disqualified due to the insolubility of 4-chlorotoluene leading to no conversion. Tertiary alcohols (entries 11–12) provided no conversion. A highly polar aprotic solvent, DMSO (entry 13), and the moderately polar aprotic solvent, THF (entry 14), also provided no conversion. For THF, this result appears to align with the predicted higher activation barrier for this reaction system in that solvent, while the rationale for the failure of the reaction in DMSO is less apparent.

**Table 5:**
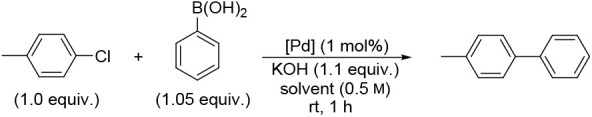
Suzuki Coupling in Various Polar Protic and Polar Aprotic Solvents

entry	solvent	yield (%)^a^
1	MeOH	15
2	EtOH	58
3	1-propanol	73
4	1-butanol	76
5	1-pentanol	68
6	2-methyl-1-butanol	59
7	3-methyl-1-butanol	65
8	2-propanol	40
9	2-butanol	43
10	2-pentanol	37
11	*tert*-amyl alcohol^b^	0
12	*tert*-butyl alcohol^b^	0
13	DMSO	0
14	THF^b^	0

a Isolated yield.

b KOtBu was used instead of KOH.

The experimental yields corroborate a general trend with the increasing polarity of the solvent as predicted from the computational model. The primary alcohols (ε = 15.13–33.0) provided higher yields than secondary alcohols (ε = 13.71–20.18), which aligns well with the higher dielectric constant of primary alcohol solvents. The failure of the less polar tertiary solvents (ε = 5.78 and 12.47) also corroborates this trend. A contributor to this trend could be steric hindrance, which might limit the participation of the solvent in stabilizing the intermediates in the order from primary to secondary to tertiary alcohols. Another interesting observation is that the solvents with the highest dielectric constants, DMSO (ε = 47.24) and methanol (ε = 33.0), provided no conversion and a 15% yield, respectively. This finding suggests that additional factors or mechanistic steps may need to be considered for determining the optimal solvent for this Suzuki–Miyaura reaction system.

## 5 Conclusions

This work investigated the impact of solvent properties on the energetics of the oxidative addition reaction in the Pd/NHC-catalyzed Suzuki–Miyaura coupling with aryl chlorides and evaluated the computational results with experimental corroboration. Data for the activation and reaction energies were predicted using density functional theory for a test reaction in over 20 selected solvents to enable an analysis of general trends and an investigation of the molecular origins of the predicted solvent effects. It was found that the activation barriers and reaction energies primarily depend on the solvent polarity, as quantified by the dielectric constant. An analysis of solute polarity and atomic charges confirmed that the lowering of the activation barrier and reaction energy can be attributed to the increased polarity of the intermediates from **I1** < **TS1** < **I2**, which is due to electron transfer from the catalyst onto the substrate during the oxidative addition. Solvents with moderate to high polarity, i.e., dielectric constants ε ≥ 12, were found to have similarly low activation barriers. Since many solvents fall into this range, this finding provides an interesting insight that might explain why the Pd/NHC-catalyzed Suzuki coupling of aryl chlorides can be performed successfully in a broad range of solvents. Moreover, this study also revealed that additional solvent properties, such as hydrogen-bond acidity and basicity, show substantial correlations with the activation barriers for the oxidative addition in this catalyst/substrate system. As corroborated by the experimental results, the overall efficiency of the reaction depends on additional factors, including the remaining mechanistic steps of the Suzuki coupling such as the formation of the active catalyst and the catalytic cycle’s sensitivities to solvent effects, or the solubilities of the reactants and catalyst, which were not studied in this work. Future studies are underway to investigate these additional aspects.

## Supplementary Material







## Notes

Solvation energy correction calculation for isoamyl alcohol, 2-methyl-1-butanol, and 2-pentanol; analysis of the sensitivity of final results on the geometry optimization approach; description of the numerical approach for the calculation of the free energy correction; numerical convergence of the free energy correction; experimental approach; ^1^H NMR spectra for the reaction product (PDF)
Electronic energies and free energies for intermediate 1, transition state 1, and intermediate 2 in 24 solvents (XLSX)
Optimized geometries obtained at the PBE-D3­(BJ)/def2-SVP level of theory (PDF)
