# Supplementary material for: Analyzing the Solvent Effects in Palladium/N‑Heterocyclic Carbene (Pd/NHC)-Catalyzed Suzuki–Miyaura Coupling of Aryl Chlorides: A Computational Study of the Oxidative Addition Step with Experimental Validation
Source: J Phys Chem B. 2025 Dec 26;130(1):362–73. doi: 10.1021/acs.jpcb.5c06092 (PMC12794135; doi:10.1021/acs.jpcb.5c06092)
Supplement: Supplementary file 1 [file jp5c06092_si_001.pdf]

Supporting Information to

“Analyzing the Solvent Effects in Palladium/N-heterocyclic Carbene (Pd/NHC) Catalyzed Suzuki-Miyaura Coupling of Aryl Chlorides: A Computational Study of the Oxidative Addition Step with Experimental Validation”

Max Collier,[1] Brandon Rodriguez,[1] Sean Lasiter,[1] Addison D. Olmsted,[1] Evan K. Simmons,[2] Gregory R. Boyce,[2] and Daniel S. Lambrecht,[1]*

[1] Department of Chemistry and Physics, Florida Gulf Coast University, 10501 FGCU Blvd. S., Fort Myers, Florida, 33965, USA

[2] Department of Chemistry and Biochemistry, East Stroudsburg University, 200 Prospect Street, East Stroudsburg, Pennsylvania, 18301, USA

Computational Approach

Solvation energy correction for isoamyl alcohol, 2-methyl-1-butanol, and 2-pentanol

Activation Barriers:

Solvent	C-PCM	SMD	$\Delta_{SMD-CPCM}$
t-BuOH	13.50	13.46	-0.03
Isoamyl alcohol	13.50	13.45	
2-methyl-1-butanol	13.50	13.45	
2-pentanol	13.50	13.45	
Benzyl alcohol	13.51	13.44	-0.07

Estimated by adding
$\Delta_{av} = \frac{1}{2}(\Delta_{t-BuOH} + \Delta_{BnOH}) = -0.05$
to C-PCM result

Reaction Energies:

Solvent	C-PCM	SMD	$\Delta_{SMD-CPCM}$
t-BuOH	-23.96	-22.60	1.36
Isoamyl alcohol	-23.88	-22.64	
2-methyl-1-butanol	-23.88	-22.63	
2-pentanol	-23.77	-22.53	
Benzyl alcohol	-23.71	-22.58	1.13

Estimated by adding
$\Delta_{av} = \frac{1}{2}(\Delta_{t-BuOH} + \Delta_{BnOH}) = 1.245$
to C-PCM result

Figure S1: Since SMD solvent parameters were not available for the listed solvents, the activation barriers and reaction energies in these solvents was estimated by correcting the C-PCM results with the average SMD-CPCM difference for the most similar solvents, t-butanol and benzyl alcohol.

Sensitivity of geometry optimizations

We noticed that the final structures of the geometry optimizations were very sensitive to the specific numerical settings and to the way in which initial geometries were generated. For example, two different approaches were tested for generating geometries to test the sensitivity to the starting geometry. Approach 1 started each optimization from the same geometries published by Meconi et al. (DOI:10.1021/acs.organomet.7b00114) for 2-propanol as the solvent. Approach 2 utilized a sequential approach, where we started with (re)optimization of the structures for 2-propanol and then utilized these structures as initial geometries for the optimization within water for the solvent. Then we sequentially stepped through solvents with decreasing polarity, where the converged geometries were used for initial geometries for the subsequent solvent. It was found that Approach 2 typically generated structures with lower energy (Table S1 and Figure S2); therefore, all calculations presented in the main text utilized geometries obtained via the sequential approach 2.

Table S1: Activation barriers calculated from TS1 structures at the PBE-D3(BJ)/def2-SVP/C-PCM level of theory (all in kcal/mol). Approach 1 means that all geometry optimizations were started from the same initial geometries. Approach 2 means that the optimized geometries for one solvent were used as initial geometries for the solvent with the next-higher polarity.

Solvent	Approach 1	Approach 2	Difference
water	8.95	8.94	0.01
ethylene glycol	8.91	8.92	0.00
MeOH	8.90	8.90	0.00
EtOH	8.88	8.86	0.02
i-PrOH	8.84	8.86	0.02
n-BuOH	8.85	8.78	0.07
t-BuOH	8.83	8.76	0.06
Benzyl alcohol	8.69	8.67	0.02
Acetone	8.85	8.84	0.01
MEK	8.83	8.81	0.02
MIBK	8.74	8.72	0.02
Ethyl acetate	8.95	8.95	0.00
i-PrOAc (n-PrOAc)	9.01	9.01	0.00
n-BuOAc	9.07	9.08	0.01
THF	8.81	8.80	0.01
Anisole	9.12	9.18	0.06
Toluene	9.62	9.48	0.14
Cyclohexane	10.04	9.64	0.40
Heptane	9.99	9.66	0.33

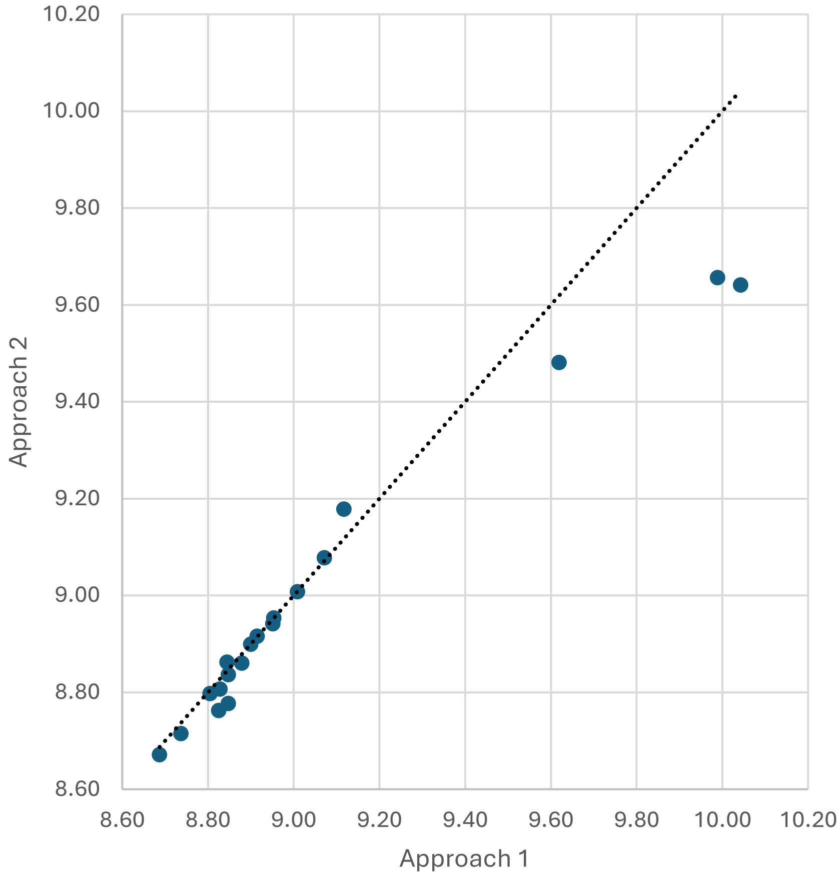

Figure S2: Comparison of activation barriers obtained via Approach 2 versus Approach 1 calculated at the PBE-D3(BJ)/def2-SVP/C-PCM level of theory (all in kcal/mol).

Figure S3. Harmonic vibrational frequencies of the lowest-lying modes in three solvents representative of a broad range of dielectric constants from low (heptane) over medium (benzyl alcohol, BnOH) to high (water). (All for TS1.)

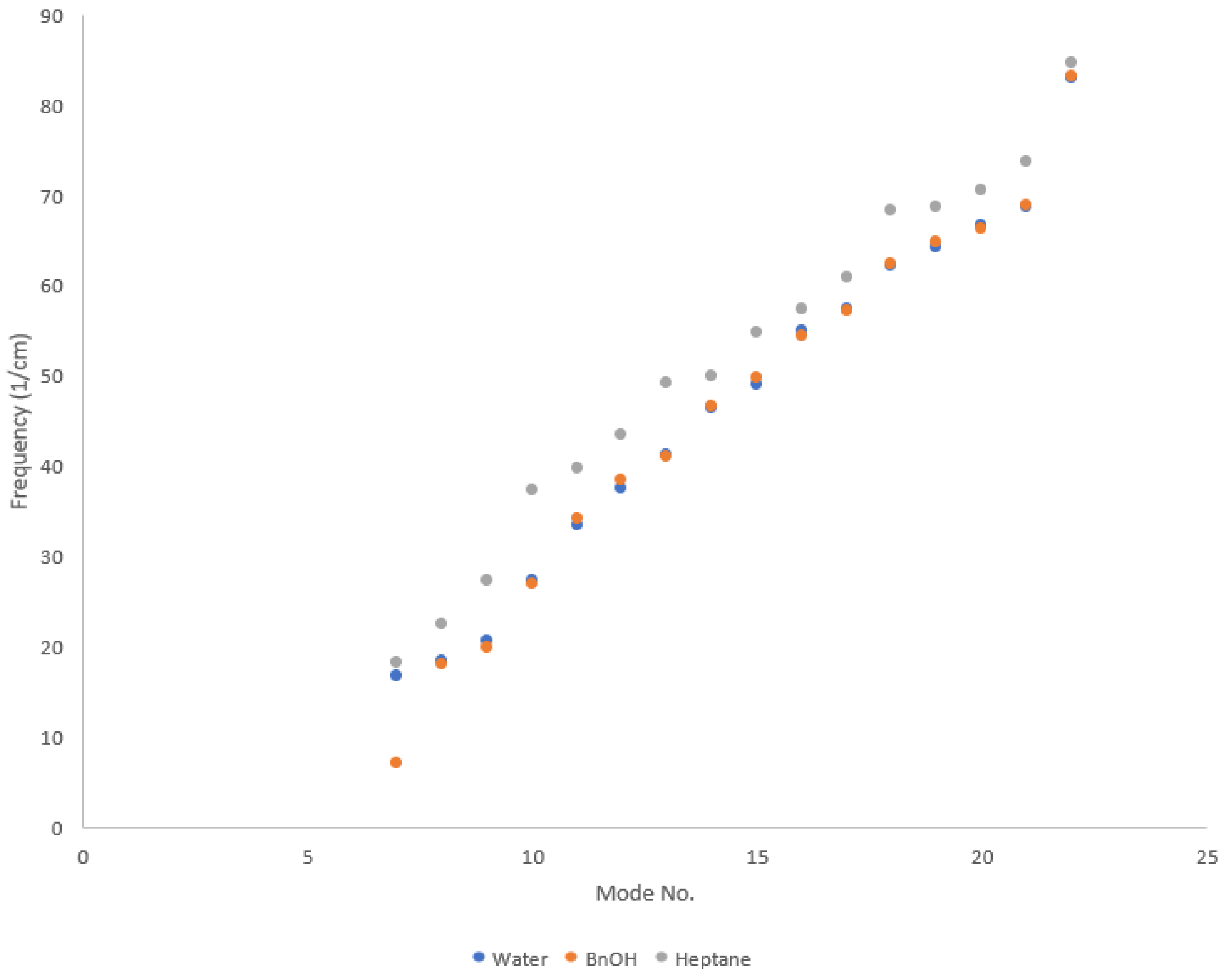

Free Energy Correction – Numerical Considerations

The free energy ΔG was calculated using the thermodynamics module implemented in ORCA.[1–4] However, the approach for calculating vibrational entropy calculations was modified for several reasons. Vibrational entropies are notoriously difficult to treat accurately due to numerical errors stemming from the incompleteness and lack of rotational invariance of the DFT integration grid. For example, free energies for transition states have been reported to vary by over 5 kcal/mol for a metalorganic test reaction using commonly employed integration grids.[5] Another potential numerical issue stems from the discretization of the surface charges of the solute cavity within implicit solvent models, which depends on the positions of the solute atoms and can therefore lead to discontinuities in potential energy surfaces and random numerical errors in calculated frequencies.[6,7] Moreover, the harmonic oscillator model is inappropriate for calculating free energies of low-frequency vibrational modes as discussed for example in Ref. [[8]], This issue led to the proposition for example of the quasiharmonic approximation, in which the harmonic vibrational frequencies lower than $100\ \mathrm{cm}^{-1}$ are raised to $100\ \mathrm{cm}^{-1}$ to avoid the breakdown of the harmonic oscillator approximation.[8]

The high sensitivity of the harmonic vibrational entropy S_{HO} to numerical errors in the low-frequency modes becomes apparent from the expression:

$$S_{HO} = R \cdot \sum_i \frac{h\nu_i}{k_B T} \cdot \frac{e^{-\frac{h\nu_i}{k_B T}}}{1 - e^{-\frac{h\nu_i}{k_B T}}} - \ln\left(1 - e^{-\frac{h\nu_i}{k_B T}}\right),$$

Where ν_i is the vibrational frequency of mode i and T the temperature (in Kelvin). h, k_B and R are Planck's and Boltzmann's constants and the gas constant, respectively. This expression approaches infinity for low-frequency modes ($\nu_i \to 0$) due to the divergence of the second (logarithmic) term. For this reason, low-frequency modes contribute strongly to the vibrational

entropy and small numerical errors in the low frequencies can have a large impact on the vibrational entropy and thereby the free energy correction.

The standard approach to alleviate this problem within the ORCA program package is the quasi-rigid rotor harmonic oscillator (QRRHO) approximation as proposed by Grimme.[9] Here, the vibrational entropies of low-frequency modes are replaced with those of a free rotor:

$$S_{vib}^{QRRHO}(\nu_i) = \big(1 - w(\nu_i)\big)S_{FR}(\nu_i) + w(\nu_i)S_{HO}(\nu_i),$$

where $w(\nu_i) = \frac{1}{1+\left(\frac{\nu_0}{\nu_i}\right)^a}$ is the damping function of Chai and Head-Gordon[10] which smoothly connects between the harmonic oscillator entropy S_{HO} with the entropy term of a free rotor S_{FR}. The standard parameters for the damping function used within ORCA are $\nu_0 = 100\ \text{cm}^{-1}$ for the cutoff frequency with an exponent of $a = 4$. The entropy for a free rotor S_{FR} is given by:

$$S_{FR}(\nu_i) = R \cdot \left[\frac{1}{2} + \ln\left(\frac{8\pi^3 \mu_i' k_B T}{h^2}\right)^{\frac{1}{2}}\right]$$

with $\mu_i' = \frac{\mu_i B_{av}}{\mu_i + B_{av}}$ and $\mu_i = \frac{h}{8\pi^2 \nu_i}$. The reduced moment of inertia μ_i' approaches a small, but finite value of $B_{av} = 10^{-44}\ kg\ m^2$ for small frequencies. The free rotor entropy expression therefore remains bounded even for small frequencies and offers improved numerical stability; consequently, the vibrational entropy calculated within the QRRHO approach has been reported to "remain reasonable even for very low frequencies".[9]

Despite improvements such as the QRRHO approximation or the quasiharmonic approximation, the accurate prediction of vibrational entropies remains challenging.[11] In fact, we observed that the vibrational entropies calculated within the QRRHO approach varied substantially even between solvents with very similar properties (**Figure S4**). For example, the free energy correction for toluene and anisole varies by 0.14 kcal/mol, although both solvents are apolar with

similar dielectric constants (4.2247 versus 2.3741) and refractive indexes (1.5174 versus 1.4961). Another clear outlier is the free energy correction for water, which at -1.71 kcal/mol is about 3 × larger than for all other solvents. These findings were surprising since one would expect that solvents with similar properties should approach the same numerical values for the vibrational entropy.

We concluded that these fluctuations could indicate numerical issues in the calculation of the vibrational entropies. In the system studied in this work, TS1 has 16 harmonic modes with frequencies below 100 cm^{-1} and 2-3 modes below 20 cm^{-1} (**Figure S3**). Numerical errors in these low-frequency modes could have a relevant impact on the predicted vibrational entropies since the free rotor entropy expression increases substantially as $\nu \rightarrow 0$. Consider for example the lowest-frequency harmonic mode in benzyl alcohol. This mode was calculated to have a harmonic frequency of 7 cm^{-1}, which corresponds to a QRRHO vibrational entropy contribution to the free energy of -1.64 kcal/mol. If there was a numerical error of ± 5 cm^{-1}, which would be a common magnitude of error for this type of calculation, the predicted vibrational entropy could change to -2.01 respectively -1.48 kcal/mol, depending on the sign of the error (**Table S2**). A numerical uncertainty of $0.16 - (-0.37) = 0.53$ kcal/mol is large compared to the solvent effects investigated in this study. Moreover, numerical errors could potentially be larger as there are several harmonic modes with frequencies below 100 cm^{-1}, which in a worst-case scenario could each contribute to the numerical noise to further increase the error in the vibrational entropy.

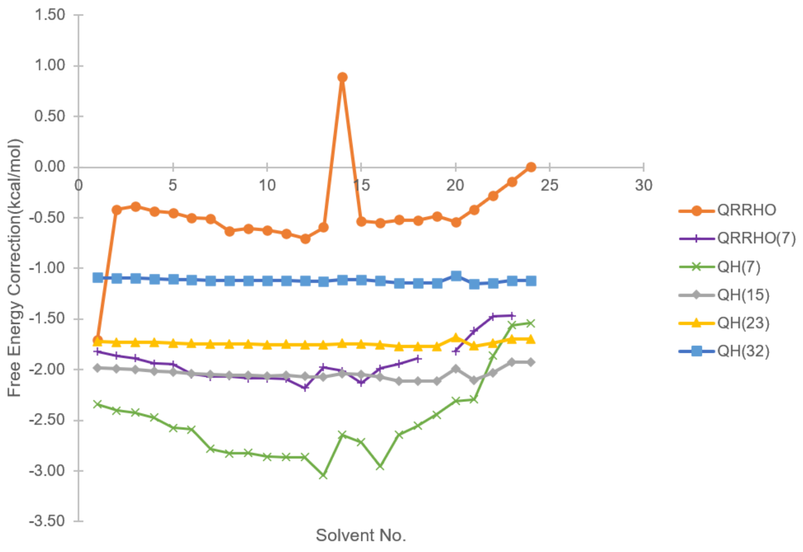

Figure S4. Magnitude of free energy correction as predicted within the QRRHO approach and the quasiharmonic (QC) approach. For the QH approach, numbers in parentheses indicate the number of lowest non-zero modes that have been raised to 100 cm^{-1}. For the QRRHO approach, numbers in parentheses indicate the number of lowest non-zero modes that were removed. The solvents are numbered from 1 (water) to 24 (heptane).

To assess the impact of numerical noise on predicted free energies, we tested a variety of approaches such as modifying the cutoff parameter and the exponent for the QRRHO approach, removing low-frequency modes from the QRRHO calculation, and a quasiharmonic (QH) approach where low-frequency modes are raised to 100 cm^{-1} (representative examples shown in **Figure S4**). Removing the seven lowest-frequency modes (corresponding to modes < 50 cm^{-1}, or modes 6-15 for TS1)[1] as in the QRRHO(7) approach or raising these frequencies to 100 cm^{-1} as in the QH(7) approach reduces the oscillations in the free energy correction. This finding supports

[1] We adopt here the mode numbering convention of ORCA, which assigns mode numbers 0-5 to the translations and rotations, so that the first non-zero frequency mode is mode 6.

our hypothesis that the variations are in fact stemming from numerical errors in the lowest-frequency modes. Raising additional modes to 100 cm^{-1} as in the QH(15) approach further smoothed out the free energy corrections, so that solvents with similar properties show similar free energy corrections. Including more than 15 modes, as in QH(23) or QH(32) changes the absolute values but not the trends in the free energy corrections. Moreover, skipping more than 7 modes in the QRRHO approach also does not lead to a qualitative change. We therefore smoothed out numerical noise by using the QH(15) approach as a compromise between removing numerical noise and keeping as many modes as feasible for all calculations reported in this study unless noted otherwise.

Table S2. Vibrational entropy contribution to the free energy correction for different harmonic frequencies (calculated for a typical rotational constant of 10^{-44} kg · m^2) at room temperature.

$\tilde{\nu}$ (cm^{-1})	$-T \cdot S_{HO}$ (kcal/mol)	$-T \cdot S_{FR}$ (kcal/mol)
0.01	−6.48	−3.18
0.1	−5.12	−2.82
1	−3.75	−2.21
2	−3.34	−2.01
3	−3.10	−1.89
4	−2.93	−1.80
5	−2.80	−1.74
6	−2.69	−1.68
7	−2.60	−1.64
8	−2.52	−1.60
9	−2.45	−1.56
10	−2.39	−1.53
11	−2.33	−1.50
12	−2.28	−1.48
13	−2.23	−1.45
14	−2.19	−1.43
15	−2.15	−1.41
16	−2.11	−1.39
17	−2.07	−1.38
18	−2.04	−1.36
19	−2.01	−1.34
20	−1.98	−1.33

Experimental Approach

General Information

All reagents and solvents were used as received from commercial sources. (IPr)Pd(cin)Cl was purchased from Aurum Pharmatech. Reactions were run under a nitrogen (N_2) atmosphere using Schlenk techniques and anhydrous solvents. Room temperature (rt) refers to 20−25 °C. Reactions were monitored by thin-layer chromatography (TLC) carried out on commercial silica gel plates (60 F_{254} silica) with visualization accomplished with UV light. Yield refers to isolated yield of analytically pure material. Yields are reported for a specific experiment and as a result may differ slightly from those found in the tables and schemes, which are averages of at least two experiments.

Procedure for the Suzuki Cross-Coupling of Phenylboronic acid and 4-Chlorotoluene. Phenylboronic acid (162 mg, 1.33 mmol, 1.05 equiv.), potassium hydroxide (78 mg, 1.40 mmol, 1.10 equiv.) and (IPr)Pd(cin)Cl (8.0 mg, 0.013 mmol, 0.01 equiv.) and a stirbar were added to a vial with a screw cap fitted with a septum. The vial was evacuated under vacuum then backfilled with N_2 (3 cycles). Solvent (2.5 mL, 0.50 M) was added via syringe and the mixture was allowed to stir for 15 minutes. 4-Chlorotoluene (0.15 mL, 1.27 mmol, 1.0 equiv.) was added to the solution then run for 1 h at room temperature. The solvent was then removed under reduced pressure and the crude residue was triturated with hexanes (10 mL). The solution was filtered and concentrated under reduced pressure. The residue was purified by column chromatography (100% hexanes) to provide 4-phenyltoluene as a white solid with spectroscopic data in accordance with the literature.

^{1}H NMR (400 MHz, $CDCl_3$) δ_H: 2.45 (3H, s), 7.30−7.32 (2H, m), 7.36−7.40 (1H, m), 7.46−7.50 (2H, m), 7.54−7.57 (2H, m), 7.62−7.65 (2H, m).

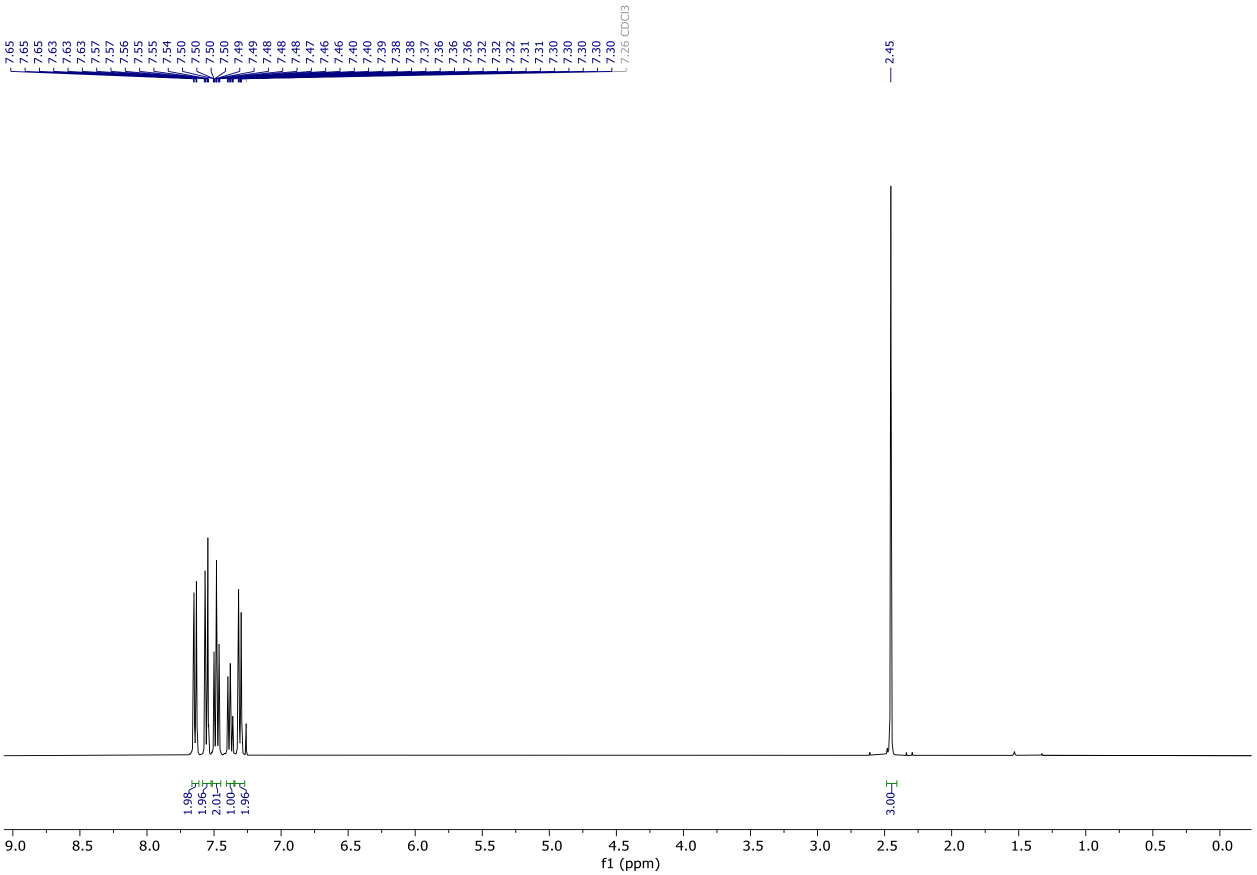

The following solvents were disqualified due to insolubility of the reagents.

Water, propylene carbonate, dihydrolevoglucosenone (Cyrene), toluene, anisole, hexane, heptane, glycerol.

References

(1) Neese, F.; Wennmohs, F.; Becker, U.; Riplinger, C. The ORCA Quantum Chemistry Program Package. *Journal of Chemical Physics* **2020**, *152* (22), 224108. https://doi.org/10.1063/5.0004608.

(2) Neese, F. Software Update: The ORCA Program System, Version 4.0. *WIREs Computational Molecular Science* **2018**, *8* (1), e1327. https://doi.org/10.1002/wcms.1327.

(3) Neese, F. Software Update: The <scp>ORCA</Scp> Program System—Version 5.0. *WIREs Computational Molecular Science* **2022**, *12* (5), e1606. https://doi.org/10.1002/wcms.1606.

(4) Neese, F. The ORCA Program System. *WIREs Computational Molecular Science* **2012**, *2* (1), 73–78. https://doi.org/10.1002/wcms.81.

(5) Bootsma, A. N.; Wheeler, S. Popular Integration Grids Can Result in Large Errors in DFT-Computed Free Energies. July 25, 2019. https://doi.org/10.26434/chemrxiv.8864204.v4.

(6) Lange, A. W.; Herbert, J. M. Polarizable Continuum Reaction-Field Solvation Models Affording Smooth Potential Energy Surfaces. *J Phys Chem Lett* **2010**, *1* (2), 556–561. https://doi.org/10.1021/jz900282c.

(7) Lange, A. W.; Herbert, J. M. A Smooth, Nonsingular, and Faithful Discretization Scheme for Polarizable Continuum Models: The Switching/Gaussian Approach. *J Chem Phys* **2010**, *133* (24), 244111. https://doi.org/10.1063/1.3511297.

(8) Ribeiro, R. F.; Marenich, A. V.; Cramer, C. J.; Truhlar, D. G. Use of Solution-Phase Vibrational Frequencies in Continuum Models for the Free Energy of Solvation. *J Phys Chem B* **2011**, *115* (49), 14556–14562. https://doi.org/10.1021/jp205508z.

(9) Grimme, S. Supramolecular Binding Thermodynamics by Dispersion-Corrected Density Functional Theory. *Chemistry – A European Journal* **2012**, *18* (32), 9955–9964. https://doi.org/10.1002/chem.201200497.

(10) Chai, J.-D.; Head-Gordon, M. Long-Range Corrected Hybrid Density Functionals with Damped Atom–Atom Dispersion Corrections. *Physical Chemistry Chemical Physics* **2008**, *10* (44), 6615. https://doi.org/10.1039/b810189b.

(11) Tarannam, N.; Alassad, N.; Lemcoff, N. G.; Kozuch, S. Right Answer for the Right Reason? Benchmarking Protocols and Pitfalls on a Ru-Metathesis Example. *J Chem Theory Comput* **2023**, *19* (15), 5024–5035. https://doi.org/10.1021/acs.jctc.3c00205.
