# Supplementary material for: Analyzing the Solvent Effects in Palladium/N‑Heterocyclic Carbene (Pd/NHC)-Catalyzed Suzuki–Miyaura Coupling of Aryl Chlorides: A Computational Study of the Oxidative Addition Step with Experimental Validation
Source: J Phys Chem B. 2025 Dec 26;130(1):362–73. doi: 10.1021/acs.jpcb.5c06092 (PMC12794135; doi:10.1021/acs.jpcb.5c06092)
Supplement: Supplementary file 3 [file jp5c06092_si_003.pdf]

```
I1_2-methoxyethanol.xyz
81
Coordinates from ORCA-job I1 E -2016.089163932224
  N      -1.041655     -1.168395      1.396183
  C      -2.389261     -0.748338      1.127127
  N       1.098106     -1.368768      1.230118
  C      -3.213009     -1.580262      0.331444
  C      -4.499985     -1.104864      0.012689
  H      -5.164201     -1.718996     -0.614081
  C      -4.943001      0.141194      0.478306
  H      -5.950016      0.498539      0.212858
  C      -4.114049      0.931230      1.287339
  H      -4.477853      1.904297      1.651083
  C      -2.818889      0.501635      1.636313
  C      -2.715729     -2.917198     -0.204732
  H      -1.800976     -3.183618      0.363368
  C      -2.310405     -2.787566     -1.683700
  H      -1.538521     -1.998024     -1.808021
  H      -1.895520     -3.745064     -2.061228
  H      -3.185465     -2.518933     -2.312060
  C      -3.733130     -4.049363      0.004516
  H      -4.654200     -3.888197     -0.592639
  H      -3.297673     -5.017451     -0.316360
  H      -4.027693     -4.139538      1.069704
  C      -1.908188      1.378052      2.488714
  H      -1.049478      0.753970      2.810911
  C      -1.338559      2.539781      1.656004
  H      -2.148635      3.213680      1.307389
  H      -0.624018      3.140844      2.255014
  H      -0.806410      2.151752      0.761392
  C      -2.606564      1.880800      3.761719
  H      -3.005002      1.040383      4.365686
  H      -1.890337      2.447911      4.390520
  H      -3.450380      2.561742      3.526421
  C       0.014877     -0.816545      0.588005
  C      -0.630384     -1.912518      2.500609
  H      -1.336799     -2.280364      3.250931
  C       0.730082     -2.037857      2.396357
  H       1.463328     -2.536508      3.037387
  C       2.450873     -1.197658      0.775725
  C       2.991688     -2.143628     -0.128761
  C       4.317732     -1.945971     -0.559594
  H       4.766785     -2.657749     -1.268806
  C       5.069093     -0.853124     -0.104016
  H       6.105286     -0.718026     -0.450746
  C       4.504906      0.070913      0.786081
  H       5.100887      0.929731      1.131452
  C       3.180327     -0.077706      1.242674
  C       2.150354     -3.286004     -0.682883
  H       1.253034     -3.381013     -0.037952
```

```
  C       2.882001     -4.635844     -0.654980
  H       3.228467     -4.888044      0.367770
  H       2.204754     -5.444010     -0.998452
  H       3.766363     -4.640198     -1.325271
  C       1.660676     -2.931588     -2.099849
  H       2.516527     -2.833097     -2.800134
  H       0.981593     -3.717889     -2.489368
  H       1.106729     -1.964480     -2.085556
  C       2.571419      0.947086      2.191676
  H       1.513074      0.664466      2.361366
  C       2.564731      2.353067      1.569106
  H       2.019857      2.355289      0.603666
  H       2.061612      3.073491      2.245947
  H       3.594215      2.724965      1.386173
  C       3.278048      0.929255      3.557744
  H       4.346771      1.213531      3.462632
  H       2.797568      1.648880      4.251937
  H       3.233179     -0.077811      4.019986
  C       0.063428      3.586679     -1.643025
  C       1.023162      2.682548     -2.106855
  C      -1.316736      3.345075     -1.816124
  C       0.606234      1.509491     -2.808210
  H       2.094113      2.890836     -1.973169
  C      -1.726404      2.167838     -2.447667
  C      -0.787384      1.229431     -2.989919
  H       1.363378      0.924904     -3.356236
  H      -2.802632      1.974646     -2.582344
  C      -1.263691      0.171853     -3.960567
  H      -1.461397      0.623918     -4.957083
  H      -0.513344     -0.630350     -4.095521
  H      -2.208901     -0.289459     -3.612969
  Cl      0.576669      5.010048     -0.749397
  H      -2.053497      4.060458     -1.424863
  Pd     -0.010199      0.261478     -1.114430
I1_PrOAc.xyz
81
Coordinates from ORCA-job I1 E -2016.086659239937
  N      -1.041785     -1.171550      1.395746
  C      -2.389394     -0.750392      1.128168
  N       1.098147     -1.372975      1.231131
  C      -3.215687     -1.582866      0.335920
  C      -4.504177     -1.108690      0.022502
  H      -5.170494     -1.723383     -0.601384
  C      -4.946088      0.137115      0.489348
  H      -5.954469      0.493591      0.228067
  C      -4.114113      0.928236      1.293793
  H      -4.476644      1.901603      1.657811
  C      -2.817397      0.499782      1.637689
  C      -2.717451     -2.917458     -0.204965
  H      -1.805655     -3.187624      0.366268
```

C	-2.303656	-2.777483	-1.680656
H	-1.533119	-1.984932	-1.794479
H	-1.884843	-3.731496	-2.062710
H	-3.175819	-2.506058	-2.311831
C	-3.736001	-4.050680	-0.008560
H	-4.653086	-3.887006	-0.611159
H	-3.298722	-5.017180	-0.331751
H	-4.038480	-4.146820	1.053889
C	-1.902747	1.378052	2.483751
H	-1.045994	0.752537	2.808619
C	-1.330035	2.531762	1.642280
H	-2.138505	3.205285	1.289514
H	-0.613343	3.135458	2.235940
H	-0.799960	2.135792	0.749843
C	-2.597116	1.891616	3.754559
H	-3.000108	1.057300	4.363975
H	-1.877504	2.458762	4.379433
H	-3.437245	2.576112	3.516452
C	0.015572	-0.819750	0.588083
C	-0.631550	-1.916037	2.500390
H	-1.338683	-2.283475	3.250098
C	0.728845	-2.042005	2.397085
H	1.461451	-2.540938	3.038467
C	2.452244	-1.201508	0.780583
C	2.996947	-2.148620	-0.120344
C	4.325490	-1.953011	-0.543566
H	4.777701	-2.665496	-1.249887
C	5.075561	-0.860810	-0.084701
H	6.113832	-0.727291	-0.425676
C	4.507158	0.064948	0.800461
H	5.101553	0.924198	1.147387
C	3.179985	-0.081655	1.249728
C	2.154637	-3.286891	-0.681281
H	1.262859	-3.391826	-0.030009
C	2.888276	-4.635778	-0.677936
H	3.249659	-4.899236	0.336790
H	2.207613	-5.441140	-1.021195
H	3.763048	-4.632351	-1.360685
C	1.652143	-2.913522	-2.088993
H	2.502447	-2.803460	-2.794286
H	0.971028	-3.695153	-2.484349
H	1.097230	-1.947036	-2.055512
C	2.565971	0.947261	2.190807
H	1.508390	0.661994	2.360447
C	2.554194	2.347538	1.555908
H	2.008763	2.337869	0.591013
H	2.048587	3.072751	2.225659
H	3.582309	2.721409	1.369179
C	3.270105	0.942950	3.558162
H	4.337855	1.231020	3.463644

```
  H       2.785504      1.665789      4.246125
  H       3.229104     -0.060643      4.028309
  C       0.066343      3.583398     -1.641893
  C       1.023434      2.677451     -2.107438
  C      -1.314423      3.343546     -1.812496
  C       0.603454      1.505022     -2.807829
  H       2.094800      2.883863     -1.975040
  C      -1.727287      2.166667     -2.442036
  C      -0.790986      1.226667     -2.985580
  H       1.358600      0.919100     -3.357104
  H      -2.804024      1.973648     -2.572120
  C      -1.271115      0.169056     -3.954350
  H      -1.468886      0.619742     -4.951534
  H      -0.523224     -0.635586     -4.088436
  H      -2.216636     -0.289704     -3.604361
  Cl      0.583135      5.005667     -0.749849
  H      -2.048829      4.060244     -1.419451
  Pd     -0.006938      0.258386     -1.113123
I1_i-PrOH.xyz
81
Coordinates from ORCA-job I1 E -2016.089469234610
  N      -1.041604     -1.168129      1.396246
  C      -2.389251     -0.748214      1.127194
  N       1.098121     -1.368239      1.229637
  C      -3.212677     -1.579904      0.330926
  C      -4.499464     -1.104292      0.011628
  H      -5.163423     -1.718227     -0.615616
  C      -4.942609      0.141686      0.477366
  H      -5.949453      0.499197      0.211487
  C      -4.114042      0.931401      1.287150
  H      -4.478021      1.904336      1.651076
  C      -2.819078      0.501609      1.636649
  C      -2.715531     -2.917036     -0.204910
  H      -1.800477     -3.183156      0.362834
  C      -2.311096     -2.788439     -1.684208
  H      -1.539027     -1.999252     -1.809613
  H      -1.896672     -3.746309     -2.061295
  H      -3.186450     -2.520048     -2.312265
  C      -3.732855     -4.049061      0.005622
  H      -4.654339     -3.888077     -0.590941
  H      -3.297649     -5.017319     -0.315079
  H      -4.026597     -4.138665      1.071084
  C      -1.908752      1.377595      2.489920
  H      -1.050531      0.753172      2.812751
  C      -1.337961      2.539293      1.657914
  H      -2.147490      3.213554      1.308714
  H      -0.623722      3.139942      2.257707
  H      -0.805202      2.151233      0.763670
  C      -2.607995      1.880478      3.762390
  H      -3.007319      1.040136      4.365873
```

H	-1.892017	2.447132	4.391886
H	-3.451282	2.561859	3.526463
C	0.014645	-0.816370	0.587716
C	-0.629921	-1.911917	2.500737
H	-1.336071	-2.279712	3.251345
C	0.730550	-2.037083	2.396141
H	1.464043	-2.535497	3.037086
C	2.450568	-1.196950	0.774421
C	2.991025	-2.142946	-0.130221
C	4.316608	-1.944762	-0.562321
H	4.765394	-2.656605	-1.271659
C	5.067815	-0.851374	-0.107780
H	6.103639	-0.715869	-0.455462
C	4.503968	0.072694	0.782557
H	5.099907	0.931840	1.127185
C	3.179858	-0.076425	1.240343
C	2.150153	-3.286409	-0.682802
H	1.251911	-3.379714	-0.038941
C	2.881780	-4.636176	-0.650246
H	3.225924	-4.885969	0.373876
H	2.205340	-5.445170	-0.993371
H	3.767683	-4.641886	-1.318492
C	1.662517	-2.935829	-2.101399
H	2.519250	-2.839189	-2.800862
H	0.984066	-3.723285	-2.489685
H	1.108427	-1.968836	-2.090722
C	2.571344	0.947825	2.190190
H	1.512286	0.666680	2.357913
C	2.567864	2.355129	1.570627
H	2.024775	2.360407	0.604185
H	2.064490	3.074712	2.248172
H	3.598163	2.726081	1.390436
C	3.276149	0.926029	3.557169
H	4.345563	1.208312	3.463824
H	2.796203	1.645295	4.252108
H	3.228675	-0.081830	4.017414
C	0.062626	3.587311	-1.642406
C	1.022794	2.683750	-2.106444
C	-1.317444	3.345080	-1.815428
C	0.606380	1.510601	-2.807965
H	2.093667	2.892493	-1.972767
C	-1.726571	2.167888	-2.447451
C	-0.787101	1.230107	-2.990085
H	1.363821	0.926385	-3.355979
H	-2.802715	1.974366	-2.582384
C	-1.262861	0.172735	-3.961227
H	-1.460859	0.625215	-4.957493
H	-0.512046	-0.628950	-4.096659
H	-2.207831	-0.289260	-3.613869
Cl	0.575254	5.010825	-0.748560

H	-2.054575	4.059915	-1.423844
Pd	-0.010906	0.261753	-1.114710

I1_Acetone.xyz
81
Coordinates from ORCA-job I1 E -2016.089467950355

N	-1.041515	-1.167974	1.395936
C	-2.389051	-0.747868	1.126636
N	1.098237	-1.368292	1.229925
C	-3.212491	-1.579455	0.330259
C	-4.499122	-1.103602	0.010661
H	-5.163074	-1.717444	-0.616684
C	-4.942128	0.142498	0.476237
H	-5.948844	0.500193	0.210114
C	-4.113598	0.932071	1.286206
H	-4.477477	1.905075	1.650070
C	-2.818789	0.502022	1.636006
C	-2.715560	-2.916776	-0.205310
H	-1.800494	-3.182884	0.362413
C	-2.311286	-2.788611	-1.684683
H	-1.539079	-1.999620	-1.810373
H	-1.897092	-3.746649	-2.061587
H	-3.186656	-2.520188	-2.312703
C	-3.733047	-4.048585	0.005605
H	-4.654593	-3.887545	-0.590847
H	-3.298032	-5.016972	-0.314964
H	-4.026610	-4.137917	1.071139
C	-1.908686	1.377771	2.489755
H	-1.049754	0.753728	2.811392
C	-1.339356	2.540981	1.658885
H	-2.149620	3.215010	1.310929
H	-0.625305	3.141469	2.259066
H	-0.806685	2.154482	0.763949
C	-2.607755	1.878444	3.763209
H	-3.005603	1.036915	4.366011
H	-1.892085	2.445299	4.392879
H	-3.452077	2.558968	3.528514
C	0.015003	-0.816226	0.587754
C	-0.630249	-1.911956	2.500445
H	-1.336663	-2.279756	3.250807
C	0.730231	-2.037265	2.396227
H	1.463477	-2.535851	3.037327
C	2.450921	-1.197338	0.775246
C	2.991489	-2.143524	-0.129173
C	4.317294	-1.945722	-0.560761
H	4.766146	-2.657688	-1.269924
C	5.068659	-0.852555	-0.105904
H	6.104655	-0.717354	-0.453193
C	4.504773	0.071615	0.784297
H	5.100845	0.930541	1.129250
C	3.180441	-0.077150	1.241616

```
  C       2.150332     -3.286568     -0.682199
  H       1.252745     -3.380786     -0.037544
  C       2.882100     -4.636310     -0.652129
  H       3.227707     -4.887209      0.371228
  H       2.205167     -5.444927     -0.995159
  H       3.767005     -4.641281     -1.321698
  C       1.661287     -2.934231     -2.099902
  H       2.517393     -2.836893     -2.800038
  H       0.982309     -3.721095     -2.488461
  H       1.107354     -1.967125     -2.087394
  C       2.571958      0.947434      2.191129
  H       1.513453      0.665279      2.360640
  C       2.566163      2.353940      1.569692
  H       2.021748      2.357366      0.603971
  H       2.063040      3.073956      2.246971
  H       3.595884      2.725548      1.387535
  C       3.278533      0.928294      3.557222
  H       4.347414      1.212021      3.462227
  H       2.798415      1.647739      4.251850
  H       3.233040     -0.079063      4.018762
  C       0.062593      3.586916     -1.643136
  C       1.022686      2.683350     -2.107354
  C      -1.317512      3.344675     -1.815818
  C       0.606161      1.510165     -2.808778
  H       2.093589      2.892157     -1.973972
  C      -1.726740      2.167404     -2.447663
  C      -0.787361      1.229622     -2.990449
  H       1.363473      0.926001     -3.357027
  H      -2.802911      1.973863     -2.582379
  C      -1.263326      0.172166     -3.961389
  H      -1.461424      0.624593     -4.957655
  H      -0.512569     -0.629562     -4.096882
  H      -2.208303     -0.289704     -3.613890
  Cl      0.575431      5.010469     -0.749417
  H      -2.054591      4.059506     -1.424122
  Pd     -0.010280      0.261646     -1.114954
I1_MeOH.xyz
81
Coordinates from ORCA-job I1 E -2016.089875051990
  N      -1.041565     -1.167286      1.396224
  C      -2.389160     -0.747596      1.126633
  N       1.098144     -1.367365      1.229741
  C      -3.211972     -1.579299      0.329693
  C      -4.498426     -1.103520      0.009136
  H      -5.161884     -1.717453     -0.618657
  C      -4.941866      0.142568      0.474396
  H      -5.948418      0.500187      0.207533
  C      -4.114001      0.932202      1.285044
  H      -4.478284      1.905130      1.648736
  C      -2.819383      0.502253      1.635764
```

C	-2.714926	-2.916937	-0.205019
H	-1.799279	-3.182204	0.362137
C	-2.312153	-2.790590	-1.684963
H	-1.539898	-2.001987	-1.812593
H	-1.898430	-3.749177	-2.060983
H	-3.188088	-2.522936	-2.312524
C	-3.731951	-4.048765	0.008206
H	-4.654227	-3.888402	-0.587297
H	-3.297064	-5.017361	-0.311900
H	-4.024128	-4.137051	1.074205
C	-1.910092	1.377972	2.490431
H	-1.050794	0.754316	2.811814
C	-1.341335	2.542380	1.660803
H	-2.151922	3.216212	1.313180
H	-0.627893	3.142762	2.261829
H	-0.808095	2.157017	0.765710
C	-2.609977	1.877056	3.764071
H	-3.007167	1.034643	4.366063
H	-1.894965	2.444022	4.394390
H	-3.454869	2.556960	3.529617
C	0.014755	-0.815272	0.587984
C	-0.630023	-1.911560	2.500418
H	-1.336249	-2.279683	3.250819
C	0.730467	-2.036749	2.395877
H	1.463871	-2.535535	3.036665
C	2.450420	-1.196476	0.773923
C	2.989702	-2.142029	-0.131892
C	4.314831	-1.943733	-0.565476
H	4.762707	-2.655302	-1.275700
C	5.066718	-0.850696	-0.111150
H	6.102144	-0.715088	-0.459990
C	4.504119	0.072798	0.780651
H	5.100694	0.931499	1.125313
C	3.180524	-0.076472	1.239948
C	2.148761	-3.285972	-0.683412
H	1.248903	-3.376629	-0.041482
C	2.879305	-4.636212	-0.644936
H	3.219614	-4.883525	0.381064
H	2.203459	-5.445602	-0.988306
H	3.767564	-4.644026	-1.310025
C	1.664872	-2.939538	-2.104274
H	2.523208	-2.845675	-2.802147
H	0.986889	-3.727863	-2.491605
H	1.111258	-1.972327	-2.098249
C	2.573400	0.947036	2.191508
H	1.514834	0.665265	2.361368
C	2.568154	2.354739	1.572741
H	2.023446	2.360513	0.607164
H	2.065671	3.073641	2.251689
H	3.598004	2.726161	1.390961

```
  C       3.280987      0.925016      3.557046
  H       4.350009      1.208082      3.461628
  H       2.801916      1.643642      4.253244
  H       3.235008     -0.083107      4.016851
  C       0.062132      3.588430     -1.642754
  C       1.022706      2.685057     -2.106388
  C      -1.317840      3.346067     -1.816381
  C       0.606784      1.511811     -2.808086
  H       2.093514      2.894086     -1.972469
  C      -1.726468      2.168754     -2.448603
  C      -0.786576      1.231031     -2.990660
  H       1.364523      0.927777     -3.355898
  H      -2.802527      1.975283     -2.584404
  C      -1.261747      0.173317     -3.961700
  H      -1.459893      0.625711     -4.957963
  H      -0.510487     -0.627947     -4.097127
  H      -2.206596     -0.289055     -3.614495
  Cl      0.574246      5.012051     -0.748575
  H      -2.055342      4.060846     -1.425351
  Pd     -0.010545      0.263382     -1.114307
I1_Toluene.xyz
81
Coordinates from ORCA-job I1 E -2016.082144203901
  N      -1.041681     -1.175110      1.394526
  C      -2.389352     -0.752949      1.128526
  N       1.098504     -1.378965      1.232539
  C      -3.218572     -1.587098      0.341458
  C      -4.509404     -1.115746      0.035407
  H      -5.178264     -1.732126     -0.583912
  C      -4.950607      0.129544      0.503378
  H      -5.961193      0.483873      0.247973
  C      -4.115026      0.923050      1.300940
  H      -4.476578      1.896605      1.665133
  C      -2.815916      0.497341      1.637953
  C      -2.717838     -2.918144     -0.205542
  H      -1.811399     -3.193825      0.371819
  C      -2.288830     -2.763056     -1.675389
  H      -1.520843     -1.965663     -1.772605
  H      -1.862841     -3.711665     -2.063002
  H      -3.155678     -2.488409     -2.312451
  C      -3.737994     -4.052943     -0.029739
  H      -4.647984     -3.886090     -0.642175
  H      -3.297303     -5.017144     -0.355145
  H      -4.054358     -4.157589      1.027840
  C      -1.896880      1.378849      2.475558
  H      -1.041976      0.752199      2.803572
  C      -1.321586      2.522870      1.623085
  H      -2.128927      3.193895      1.263516
  H      -0.604516      3.132148      2.210348
  H      -0.792235      2.117478      0.734389
```

C	-2.586300	1.905143	3.743765
H	-2.995078	1.078423	4.359653
H	-1.862837	2.472345	4.364080
H	-3.421854	2.594097	3.502520
C	0.017454	-0.822486	0.588544
C	-0.633734	-1.922214	2.498321
H	-1.342375	-2.289912	3.246227
C	0.726502	-2.049729	2.396678
H	1.457625	-2.550633	3.037929
C	2.454694	-1.208162	0.787786
C	3.003894	-2.156912	-0.108523
C	4.336549	-1.966141	-0.519525
H	4.792790	-2.680237	-1.221373
C	5.086034	-0.876346	-0.054845
H	6.127697	-0.746569	-0.386602
C	4.512390	0.052298	0.823121
H	5.105482	0.911254	1.172841
C	3.181236	-0.089779	1.261108
C	2.159630	-3.288255	-0.680300
H	1.272223	-3.403246	-0.024461
C	2.892837	-4.636981	-0.703941
H	3.269842	-4.913899	0.301463
H	2.207703	-5.438243	-1.047820
H	3.757166	-4.625974	-1.399762
C	1.646856	-2.891706	-2.078036
H	2.492794	-2.768012	-2.786275
H	0.964224	-3.667145	-2.482884
H	1.091523	-1.925965	-2.023835
C	2.560296	0.946306	2.189500
H	1.505021	0.655100	2.363009
C	2.535579	2.335372	1.531222
H	1.985703	2.302618	0.569593
H	2.027477	3.069439	2.189171
H	3.560123	2.713341	1.333201
C	3.264316	0.968256	3.556487
H	4.329496	1.264778	3.459458
H	2.773421	1.696386	4.234310
H	3.233222	-0.028634	4.041446
C	0.073224	3.582026	-1.639074
C	1.023934	2.671997	-2.109420
C	-1.309050	3.346926	-1.803998
C	0.596333	1.501192	-2.807559
H	2.096418	2.874365	-1.981409
C	-1.729705	2.171159	-2.429314
C	-0.800054	1.226973	-2.975620
H	1.346390	0.912406	-3.360615
H	-2.807668	1.979887	-2.550254
C	-1.289460	0.169056	-3.939423
H	-1.489853	0.617012	-4.937398
H	-0.546418	-0.640038	-4.073683

```
  H      -2.234666     -0.285062     -3.582827
  Cl      0.598993      5.001006     -0.749731
  H      -2.037932      4.067200     -1.407605
  Pd      0.000930      0.258261     -1.108845
I1_Cyclohexane.xyz
81
Coordinates from ORCA-job I1 E -2016.080859560967
  N      -1.042477     -1.175698      1.395818
  C      -2.390511     -0.754327      1.130279
  N       1.097729     -1.379937      1.233466
  C      -3.219911     -1.589705      0.344787
  C      -4.511874     -1.120334      0.041029
  H      -5.180974     -1.737842     -0.576862
  C      -4.953939      0.124417      0.509334
  H      -5.965553      0.477145      0.255856
  C      -4.117817      0.919534      1.304490
  H      -4.479831      1.892946      1.668542
  C      -2.817541      0.495869      1.639248
  C      -2.717445     -2.919232     -0.204233
  H      -1.811485     -3.195281      0.373775
  C      -2.286214     -2.759676     -1.672972
  H      -1.519730     -1.960343     -1.766600
  H      -1.857880     -3.706426     -2.062560
  H      -3.152594     -2.484954     -2.310633
  C      -3.736448     -4.055625     -0.032668
  H      -4.645583     -3.888864     -0.646400
  H      -3.294215     -5.018669     -0.359392
  H      -4.054897     -4.163019      1.024001
  C      -1.897636      1.379781      2.473286
  H      -1.041827      0.754235      2.801168
  C      -1.324603      2.521805      1.616719
  H      -2.133391      3.189464      1.254261
  H      -0.609313      3.135336      2.201659
  H      -0.794187      2.114250      0.729616
  C      -2.584980      1.908699      3.741500
  H      -2.993157      1.083511      4.359831
  H      -1.860660      2.477257      4.359551
  H      -3.420770      2.597385      3.500323
  C       0.016640     -0.823103      0.589546
  C      -0.634411     -1.922837      2.499544
  H      -1.342978     -2.290450      3.247494
  C       0.725750     -2.050593      2.397663
  H       1.456897     -2.551608      3.038718
  C       2.454116     -1.209439      0.789133
  C       3.002933     -2.157427     -0.108129
  C       4.336106     -1.967687     -0.517577
  H       4.792114     -2.681343     -1.219987
  C       5.086454     -0.879525     -0.050766
  H       6.128542     -0.750516     -0.381429
  C       4.513032      0.048626      0.827674
```

```
  H       5.106585      0.906758      1.178598
  C       3.181436     -0.092485      1.264360
  C       2.157659     -3.286517     -0.682814
  H       1.270023     -3.402123     -0.027344
  C       2.889329     -4.635992     -0.709768
  H       3.267057     -4.915568      0.294623
  H       2.203264     -5.435918     -1.054891
  H       3.753099     -4.624679     -1.406279
  C       1.645375     -2.885564     -2.079443
  H       2.491574     -2.759969     -2.787019
  H       0.962539     -3.659407     -2.486996
  H       1.090559     -1.919639     -2.022204
  C       2.560134      0.944669      2.191284
  H       1.506319      0.650640      2.368885
  C       2.528417      2.330534      1.526610
  H       1.974558      2.290746      0.567592
  H       2.020993      3.066615      2.182778
  H       3.551000      2.710492      1.322286
  C       3.267872      0.974669      3.556093
  H       4.331669      1.274910      3.455495
  H       2.776203      1.703564      4.232513
  H       3.242150     -0.020368      4.045144
  C       0.079019      3.582755     -1.638870
  C       1.028447      2.670897     -2.108074
  C      -1.303474      3.349327     -1.804542
  C       0.599508      1.499986     -2.805003
  H       2.101072      2.871678     -1.979185
  C      -1.725594      2.173742     -2.428881
  C      -0.797166      1.227655     -2.973762
  H       1.349052      0.909291     -3.356671
  H      -2.803749      1.983534     -2.549466
  C      -1.287654      0.169702     -3.936986
  H      -1.487238      0.616874     -4.935496
  H      -0.545769     -0.640639     -4.070173
  H      -2.233333     -0.283151     -3.580041
  Cl      0.606120      5.001188     -0.750268
  H      -2.031263      4.071139     -1.409035
  Pd      0.000482      0.258525     -1.106671
I1_Benzylalcohol.xyz
81
Coordinates from ORCA-job I1 E -2016.088774782738
  N      -1.041764     -1.168980      1.396189
  C      -2.389393     -0.748774      1.127429
  N       1.098023     -1.369416      1.230269
  C      -3.213566     -1.580739      0.332267
  C      -4.500811     -1.105534      0.014457
  H      -5.165377     -1.719715     -0.611876
  C      -4.943654      0.140438      0.480389
  H      -5.950906      0.497646      0.215675
  C      -4.114196      0.930606      1.288712
```

H	-4.477795	1.903696	1.652566
C	-2.818760	0.501192	1.636779
C	-2.716131	-2.917241	-0.204802
H	-1.801770	-3.184247	0.363678
C	-2.309643	-2.785817	-1.683296
H	-1.538120	-1.995636	-1.805961
H	-1.893999	-3.742666	-2.061646
H	-3.184360	-2.516859	-2.311994
C	-3.733644	-4.049644	0.002435
H	-4.654179	-3.888046	-0.595430
H	-3.297908	-5.017426	-0.318985
H	-4.029300	-4.140900	1.067229
C	-1.907351	1.377887	2.488114
H	-1.049058	0.753501	2.810867
C	-1.337034	2.538135	1.653837
H	-2.146784	3.212000	1.304441
H	-0.622092	3.139623	2.251924
H	-0.805239	2.148656	0.759632
C	-2.605072	1.882688	3.760651
H	-3.004458	1.043413	4.365585
H	-1.888223	2.449746	4.388788
H	-3.448132	2.564352	3.524749
C	0.014810	-0.817322	0.587904
C	-0.630524	-1.912762	2.500871
H	-1.336975	-2.280348	3.251264
C	0.729934	-2.038130	2.396718
H	1.463174	-2.536534	3.037923
C	2.450965	-1.198150	0.776408
C	2.992457	-2.144234	-0.127543
C	4.318809	-1.946674	-0.557350
H	4.768386	-2.658497	-1.266158
C	5.069828	-0.853780	-0.101385
H	6.106276	-0.718740	-0.447361
C	4.504928	0.070449	0.787993
H	5.100555	0.929451	1.133500
C	3.180024	-0.078083	1.243605
C	2.151089	-3.286105	-0.682626
H	1.254771	-3.382854	-0.036527
C	2.883296	-4.635693	-0.658766
H	3.232267	-4.889687	0.362689
H	2.205596	-5.443499	-1.002186
H	3.766094	-4.638662	-1.331122
C	1.659082	-2.928683	-2.098051
H	2.513934	-2.828215	-2.799274
H	0.979714	-3.714317	-2.488425
H	1.104851	-1.961721	-2.080524
C	2.570240	0.947201	2.191486
H	1.511975	0.664203	2.360982
C	2.562888	2.352441	1.567314
H	2.018131	2.353067	0.601840

```
  H       2.059203      3.073438      2.243107
  H       3.592187      2.724722      1.384105
  C       3.276328      0.931165      3.557838
  H       4.344939      1.215955      3.462985
  H       2.795191      1.651210      4.251141
  H       3.231957     -0.075443      4.021139
  C       0.064661      3.585729     -1.642905
  C       1.023832      2.681186     -2.107058
  C      -1.315645      3.344563     -1.815464
  C       0.606231      1.508286     -2.808251
  H       2.094884      2.888973     -1.973535
  C      -1.726009      2.167502     -2.446796
  C      -0.787563      1.228799     -2.989447
  H       1.362977      0.923333     -3.356421
  H      -2.802352      1.974474     -2.580676
  C      -1.264646      0.171430     -3.959952
  H      -1.462303      0.623448     -4.956508
  H      -0.514806     -0.631251     -4.094893
  H      -2.209975     -0.289383     -3.612030
  Cl      0.578707      5.008882     -0.749582
  H      -2.051921      4.060142     -1.423687
  Pd     -0.009996      0.260452     -1.114512
I1_THF.xyz
81
Coordinates from ORCA-job I1 E -2016.087615079234
  N      -1.042189     -1.171257      1.395939
  C      -2.389846     -0.750237      1.128425
  N       1.097742     -1.371580      1.230598
  C      -3.215821     -1.582333      0.335369
  C      -4.503887     -1.107502      0.020826
  H      -5.169919     -1.721845     -0.603740
  C      -4.945708      0.138470      0.487506
  H      -5.953702      0.495424      0.225339
  C      -4.114169      0.929095      1.293038
  H      -4.476727      1.902457      1.657113
  C      -2.817882      0.499990      1.637997
  C      -2.718293     -2.917648     -0.204450
  H      -1.805527     -3.186974      0.365578
  C      -2.307460     -2.780573     -1.681245
  H      -1.536135     -1.989268     -1.798291
  H      -1.890435     -3.735740     -2.062341
  H      -3.180570     -2.509343     -2.311198
  C      -3.736706     -4.050381     -0.004171
  H      -4.655089     -3.887142     -0.604902
  H      -3.300207     -5.017370     -0.326948
  H      -4.036540     -4.144867      1.059175
  C      -1.903954      1.377554      2.485643
  H      -1.046104      0.752630      2.808630
  C      -1.333285      2.534366      1.646960
  H      -2.142748      3.207968      1.296522
```

H	-0.616876	3.137109	2.241972
H	-0.803196	2.141420	0.753228
C	-2.598633	1.886866	3.758020
H	-2.999001	1.050121	4.365824
H	-1.879797	2.454228	4.383598
H	-3.440638	2.569747	3.521883
C	0.014662	-0.819170	0.587958
C	-0.631206	-1.915318	2.500569
H	-1.337867	-2.283027	3.250641
C	0.729244	-2.040610	2.396788
H	1.462318	-2.539128	3.038018
C	2.451302	-1.199671	0.778659
C	2.995228	-2.146486	-0.123076
C	4.322772	-1.949541	-0.549079
H	4.774294	-2.661787	-1.256130
C	5.072650	-0.856458	-0.091840
H	6.110112	-0.721913	-0.434909
C	4.505200	0.068711	0.794707
H	5.099684	0.928246	1.140809
C	3.179013	-0.079192	1.246615
C	2.153719	-3.286741	-0.681224
H	1.261145	-3.389729	-0.030797
C	2.888080	-4.635248	-0.671777
H	3.246514	-4.895255	0.344881
H	2.208708	-5.441964	-1.014424
H	3.764857	-4.633182	-1.351957
C	1.653063	-2.919162	-2.091091
H	2.504129	-2.811866	-2.795897
H	0.972631	-3.702619	-2.483991
H	1.097757	-1.952849	-2.062480
C	2.566379	0.948006	2.190513
H	1.508340	0.663867	2.359288
C	2.557209	2.350728	1.560906
H	2.012699	2.346129	0.595402
H	2.052102	3.073860	2.233311
H	3.586012	2.723858	1.376575
C	3.270475	0.937880	3.557913
H	4.338715	1.224356	3.464021
H	2.787067	1.659301	4.248202
H	3.227525	-0.067229	4.024617
C	0.067753	3.583953	-1.643679
C	1.025716	2.678226	-2.107863
C	-1.312829	3.343573	-1.815133
C	0.606711	1.505204	-2.807894
H	2.096951	2.885015	-1.974679
C	-1.724653	2.166469	-2.445163
C	-0.787431	1.226798	-2.988047
H	1.362656	0.919082	-3.355877
H	-2.801225	1.973625	-2.577137
C	-1.266099	0.169579	-3.957966

```
  H      -1.463301      0.621065     -4.954881
  H      -0.517500     -0.634373     -4.092284
  H      -2.211807     -0.289888     -3.609340
  Cl      0.583326      5.007163     -0.751916
  H      -2.048029      4.059982     -1.422960
  Pd     -0.009709      0.258015     -1.114327
I1_water.xyz
81
Coordinates from ORCA-job I1 E -2016.090282890268
  N      -1.041504     -1.166267      1.396460
  C      -2.389144     -0.747095      1.126323
  N       1.098173     -1.366228      1.229762
  C      -3.211133     -1.578920      0.328609
  C      -4.497355     -1.103229      0.006812
  H      -5.160158     -1.717239     -0.621615
  C      -4.941398      0.142800      0.471744
  H      -5.947737      0.500317      0.203925
  C      -4.114459      0.932437      1.283405
  H      -4.479309      1.905205      1.646998
  C      -2.820082      0.502611      1.635298
  C      -2.713875     -2.916909     -0.205071
  H      -1.797800     -3.181333      0.361762
  C      -2.312303     -2.792469     -1.685502
  H      -1.539965     -2.004265     -1.814934
  H      -1.898971     -3.751618     -2.060500
  H      -3.188675     -2.525599     -2.312789
  C      -3.730539     -4.048700      0.010272
  H      -4.653400     -3.888980     -0.584494
  H      -3.295720     -5.017518     -0.309250
  H      -4.021540     -4.135902      1.076680
  C      -1.911951      1.378206      2.491359
  H      -1.052065      0.755116      2.812222
  C      -1.344208      2.544487      1.663631
  H      -2.155331      3.218264      1.317092
  H      -0.631247      3.144405      2.265715
  H      -0.810599      2.161062      0.767945
  C      -2.612916      1.874780      3.765399
  H      -3.009085      1.030985      4.366118
  H      -1.898810      2.441806      4.396690
  H      -3.458655      2.553813      3.531469
  C       0.014687     -0.814352      0.588110
  C      -0.629794     -1.910362      2.500697
  H      -1.335902     -2.278477      3.251239
  C       0.730712     -2.035473      2.396018
  H       1.464220     -2.534128      3.036818
  C       2.450118     -1.195679      0.772901
  C       2.988160     -2.141007     -0.133886
  C       4.312610     -1.942371     -0.569540
  H       4.759522     -2.653821     -1.280523
  C       5.065000     -0.849255     -0.116183
```

```
  H       6.099846     -0.713356     -0.466648
  C       4.503722      0.073862      0.776926
  H       5.100824      0.932424      1.121037
  C       3.180871     -0.075779      1.238301
  C       2.147266     -3.285907     -0.683525
  H       1.246193     -3.374058     -0.042998
  C       2.877231     -4.636324     -0.639152
  H       3.214429     -4.880705      0.388573
  H       2.202055     -5.446461     -0.982088
  H       3.767414     -4.646120     -1.301636
  C       1.666193     -2.944217     -2.106467
  H       2.525727     -2.852882     -2.803198
  H       0.988873     -3.733845     -2.492286
  H       1.112569     -1.977088     -2.105015
  C       2.575285      0.946826      2.191838
  H       1.516673      0.665545      2.362325
  C       2.570664      2.355670      1.575585
  H       2.025925      2.363721      0.610006
  H       2.068648      3.073500      2.256026
  H       3.600681      2.726897      1.394394
  C       3.284155      0.922109      3.556682
  H       4.353316      1.204402      3.460516
  H       2.806341      1.640114      4.254382
  H       3.237682     -0.086668      4.014987
  C       0.061999      3.589168     -1.642418
  C       1.022847      2.685894     -2.105762
  C      -1.317899      3.347014     -1.816920
  C       0.607213      1.512889     -2.808056
  H       2.093624      2.894911     -1.971415
  C      -1.726214      2.169879     -2.449785
  C      -0.786050      1.232135     -2.991409
  H       1.365186      0.929020     -3.355739
  H      -2.802219      1.976758     -2.586638
  C      -1.260723      0.174366     -3.962607
  H      -1.458906      0.626904     -4.958785
  H      -0.509111     -0.626544     -4.098154
  H      -2.205530     -0.288334     -3.615698
  Cl      0.573748      5.012534     -0.747414
  H      -2.055670      4.061749     -1.426271
  Pd     -0.010693      0.264569     -1.114210
I1_Anisole.xyz
81
Coordinates from ORCA-job I1 E -2016.085554911299
  N      -1.041803     -1.172692      1.395447
  C      -2.389444     -0.751282      1.128338
  N       1.098208     -1.374563      1.231505
  C      -3.216579     -1.584213      0.337539
  C      -4.505723     -1.110781      0.026151
  H      -5.172761     -1.725916     -0.596486
  C      -4.947403      0.134910      0.493292
```

H	-5.956380	0.490831	0.233608
C	-4.114396	0.926692	1.295829
H	-4.476617	1.900143	1.659850
C	-2.817021	0.498954	1.637827
C	-2.717710	-2.917862	-0.205008
H	-1.807299	-3.189486	0.367811
C	-2.299964	-2.773830	-1.679204
H	-1.530123	-1.979945	-1.788650
H	-1.879229	-3.726396	-2.062777
H	-3.170778	-2.501574	-2.311881
C	-3.736696	-4.051533	-0.014066
H	-4.651927	-3.886997	-0.619254
H	-3.298499	-5.017404	-0.337896
H	-4.042816	-4.149973	1.047128
C	-1.901068	1.378133	2.481459
H	-1.045036	0.752183	2.807509
C	-1.327212	2.528770	1.636653
H	-2.135134	3.201869	1.281945
H	-0.610081	3.133790	2.228384
H	-0.797569	2.129783	0.745274
C	-2.594109	1.895830	3.751288
H	-2.998997	1.063902	4.362716
H	-1.873331	2.462986	4.374799
H	-3.432749	2.581716	3.511961
C	0.015963	-0.820687	0.588165
C	-0.632067	-1.917638	2.499997
H	-1.339548	-2.285090	3.249309
C	0.728300	-2.043882	2.397121
H	1.460590	-2.543133	3.038545
C	2.452825	-1.203049	0.782465
C	2.998904	-2.150528	-0.117209
C	4.328474	-1.955895	-0.537313
H	4.781867	-2.668679	-1.242509
C	5.078176	-0.864132	-0.076992
H	6.117295	-0.731362	-0.415622
C	4.508220	0.062288	0.806286
H	5.102090	0.921609	1.153894
C	3.180025	-0.083395	1.252625
C	2.156225	-3.287130	-0.680934
H	1.265903	-3.395134	-0.028094
C	2.890120	-4.635822	-0.685394
H	3.256142	-4.903020	0.326688
H	2.208293	-5.440162	-1.028723
H	3.761848	-4.630047	-1.372002
C	1.650322	-2.907335	-2.085739
H	2.499171	-2.793334	-2.792150
H	0.968767	-3.687352	-2.483528
H	1.095137	-1.941109	-2.046204
C	2.564031	0.947205	2.190503
H	1.506863	0.660663	2.360444

```
  C       2.549696      2.344949      1.550234
  H       2.003746      2.330002      0.585769
  H       2.043176      3.072221      2.217017
  H       3.577112      2.719769      1.361499
  C       3.267476      0.948815      3.558162
  H       4.334743      1.238636      3.463604
  H       2.781228      1.672977      4.243565
  H       3.228367     -0.053250      4.031731
  C       0.069248      3.582531     -1.641477
  C       1.024188      2.675020     -2.108330
  C      -1.312055      3.344626     -1.810408
  C       0.601555      1.503205     -2.808085
  H       2.095973      2.879823     -1.977200
  C      -1.727612      2.168194     -2.438729
  C      -0.793569      1.226601     -2.983047
  H       1.355021      0.916160     -3.358437
  H      -2.804787      1.976152     -2.566283
  C      -1.276793      0.169033     -3.950335
  H      -1.475237      0.619013     -4.947727
  H      -0.530553     -0.637121     -4.084538
  H      -2.222384     -0.288034     -3.598384
  Cl      0.589205      5.003798     -0.750299
  H      -2.044626      4.062632     -1.416422
  Pd     -0.005048      0.258029     -1.112123
I1_2-pentanol.xyz
81
Coordinates from ORCA-job I1 E -2016.088857953035
  N      -1.041634     -1.168951      1.395942
  C      -2.389189     -0.748579      1.127087
  N       1.098164     -1.369400      1.230192
  C      -3.213340     -1.580350      0.331692
  C      -4.500410     -1.104858      0.013576
  H      -5.164945     -1.718872     -0.612959
  C      -4.943122      0.141180      0.479479
  H      -5.950230      0.498614      0.214521
  C      -4.113728      0.931108      1.288116
  H      -4.477246      1.904221      1.651997
  C      -2.818460      0.501407      1.636476
  C      -2.716136     -2.917027     -0.205162
  H      -1.801755     -3.184048      0.363274
  C      -2.309820     -2.786090     -1.683741
  H      -1.538037     -1.996226     -1.806742
  H      -1.894554     -3.743178     -2.061897
  H      -3.184527     -2.517003     -2.312399
  C      -3.733826     -4.049201      0.002497
  H      -4.654431     -3.887544     -0.595244
  H      -3.298311     -5.017132     -0.318776
  H      -4.029296     -4.140153      1.067368
  C      -1.907172      1.377797      2.488258
  H      -1.048765      0.753396      2.810664
```

C	-1.337048	2.538630	1.654670
H	-2.146878	3.212610	1.305679
H	-0.622169	3.139836	2.253119
H	-0.805213	2.149744	0.760241
C	-2.605010	1.881730	3.761081
H	-3.004081	1.041996	4.365586
H	-1.888318	2.448687	4.389488
H	-3.448315	2.563220	3.525546
C	0.015022	-0.817211	0.587806
C	-0.630517	-1.912908	2.500546
H	-1.337044	-2.280590	3.250825
C	0.729949	-2.038296	2.396496
H	1.463106	-2.536853	3.037680
C	2.451137	-1.198193	0.776405
C	2.992701	-2.144483	-0.127297
C	4.319083	-1.947006	-0.557067
H	4.768721	-2.658992	-1.265674
C	5.070064	-0.853999	-0.101287
H	6.106543	-0.719042	-0.447204
C	4.505094	0.070428	0.787849
H	5.100709	0.929481	1.133253
C	3.180149	-0.078006	1.243396
C	2.151388	-3.286517	-0.682134
H	1.255055	-3.383154	-0.036042
C	2.883650	-4.636068	-0.657920
H	3.232557	-4.889797	0.363623
H	2.205993	-5.443975	-1.001185
H	3.766488	-4.639144	-1.330223
C	1.659427	-2.929498	-2.097679
H	2.514299	-2.829247	-2.798909
H	0.980077	-3.715255	-2.487836
H	1.105187	-1.962544	-2.080507
C	2.570337	0.947437	2.191091
H	1.511977	0.664654	2.360355
C	2.563440	2.352696	1.566948
H	2.018991	2.353459	0.601292
H	2.059656	3.073749	2.242609
H	3.592861	2.724800	1.384076
C	3.276107	0.931254	3.557612
H	4.344796	1.215818	3.462975
H	2.794955	1.651399	4.250800
H	3.231399	-0.075352	4.020881
C	0.063824	3.585940	-1.642836
C	1.023120	2.681684	-2.107300
C	-1.316466	3.344437	-1.815057
C	0.605647	1.508733	-2.808497
H	2.094158	2.889767	-1.974094
C	-1.726678	2.167316	-2.446392
C	-0.788119	1.228899	-2.989351
H	1.362401	0.924049	-3.356945

```
  H      -2.803006      1.974049     -2.580073
  C      -1.265170      0.171504     -3.959841
  H      -1.463115      0.623591     -4.956307
  H      -0.515178     -0.630992     -4.095024
  H      -2.210345     -0.289527     -3.611794
  Cl      0.577740      5.009184     -0.749546
  H      -2.052844      4.059786     -1.423047
  Pd     -0.009765      0.260637     -1.114607
I1_MIBK.xyz
81
Coordinates from ORCA-job I1 E -2016.088832978082
  N      -1.041673     -1.168903      1.396072
  C      -2.389269     -0.748666      1.127208
  N       1.098123     -1.369327      1.230268
  C      -3.213359     -1.580559      0.331879
  C      -4.500498     -1.105227      0.013811
  H      -5.164994     -1.719338     -0.612669
  C      -4.943331      0.140774      0.479691
  H      -5.950495      0.498080      0.214772
  C      -4.113989      0.930829      1.288252
  H      -4.477600      1.903915      1.652109
  C      -2.818656      0.501292      1.636567
  C      -2.715990     -2.917171     -0.204985
  H      -1.801728     -3.184206      0.363640
  C      -2.309330     -2.786049     -1.683454
  H      -1.537613     -1.996072     -1.806189
  H      -1.893861     -3.743051     -2.061606
  H      -3.183925     -2.517003     -2.312286
  C      -3.733662     -4.049425      0.002308
  H      -4.654113     -3.887796     -0.595679
  H      -3.297995     -5.017302     -0.318923
  H      -4.029429     -4.140486      1.067088
  C      -1.907394      1.377825      2.488230
  H      -1.049108      0.753411      2.810942
  C      -1.337020      2.538334      1.654356
  H      -2.146740      3.212283      1.305050
  H      -0.622165      3.139660      2.252712
  H      -0.805109      2.149116      0.760111
  C      -2.605328      1.882247      3.760803
  H      -3.004673      1.042770      4.365485
  H      -1.888618      2.449235      4.389162
  H      -3.448450      2.563854      3.524956
  C       0.014951     -0.817202      0.587876
  C      -0.630501     -1.912760      2.500727
  H      -1.336997     -2.280395      3.251058
  C       0.729963     -2.038127      2.396644
  H       1.463154     -2.536602      3.037852
  C       2.451068     -1.198149      0.776394
  C       2.992503     -2.144350     -0.127473
  C       4.318859     -1.946892     -0.557328
```

```
  H       4.768396     -2.658814     -1.266065
  C       5.069937     -0.853985     -0.101479
  H       6.106391     -0.719034     -0.447473
  C       4.505089      0.070363      0.787816
  H       5.100769      0.929357      1.133254
  C       3.180179     -0.078058      1.243457
  C       2.151112     -3.286290     -0.682386
  H       1.254657     -3.382761     -0.036440
  C       2.883200     -4.635935     -0.657948
  H       3.231847     -4.889662      0.363684
  H       2.205527     -5.443779     -1.001331
  H       3.766194     -4.639146     -1.330045
  C       1.659433     -2.929297     -2.098030
  H       2.514428     -2.829131     -2.799122
  H       0.980091     -3.715017     -2.488273
  H       1.105258     -1.962304     -2.080978
  C       2.570477      0.947338      2.191274
  H       1.512156      0.664504      2.360705
  C       2.563408      2.352589      1.567113
  H       2.018751      2.353316      0.601578
  H       2.059768      3.073650      2.242873
  H       3.592784      2.724710      1.384010
  C       3.276470      0.931192      3.557679
  H       4.345129      1.215811      3.462876
  H       2.795397      1.651314      4.250945
  H       3.231892     -0.075413      4.020963
  C       0.064142      3.585904     -1.642762
  C       1.023326      2.681510     -2.107185
  C      -1.316173      3.344611     -1.815070
  C       0.605716      1.508623     -2.808406
  H       2.094386      2.889434     -1.973921
  C      -1.726529      2.167543     -2.446402
  C      -0.788085      1.228973     -2.989287
  H       1.362408      0.923836     -3.356830
  H      -2.802879      1.974424     -2.580117
  C      -1.265256      0.171602     -3.959745
  H      -1.463191      0.623677     -4.956219
  H      -0.515344     -0.630970     -4.094922
  H      -2.210459     -0.289341     -3.611655
  Cl      0.578215      5.009056     -0.749427
  H      -2.052459      4.060088     -1.423123
  Pd     -0.009802      0.260617     -1.114542
I1_n-BuOAc.xyz
81
Coordinates from ORCA-job I1 E -2016.086275177759
  N      -1.041863     -1.172183      1.395599
  C      -2.389509     -0.750947      1.128296
  N       1.098116     -1.373540      1.231168
  C      -3.216158     -1.583566      0.336606
  C      -4.504928     -1.109679      0.024069
```

H	-5.171547	-1.724515	-0.599337
C	-4.946749	0.136043	0.491140
H	-5.955387	0.492298	0.230563
C	-4.114337	0.927397	1.294836
H	-4.476741	1.900774	1.658922
C	-2.817327	0.499232	1.637877
C	-2.717687	-2.917759	-0.205020
H	-1.806381	-3.188490	0.366753
C	-2.302490	-2.776142	-1.680164
H	-1.532206	-1.983080	-1.792316
H	-1.882988	-3.729585	-2.062892
H	-3.174181	-2.504340	-2.311828
C	-3.736374	-4.051184	-0.010701
H	-4.652795	-3.887157	-0.614217
H	-3.298744	-5.017415	-0.334225
H	-4.040164	-4.148273	1.051287
C	-1.902101	1.377880	2.482902
H	-1.045553	0.752241	2.808112
C	-1.329116	2.530456	1.640088
H	-2.137466	3.203779	1.286703
H	-0.612204	3.134683	2.232926
H	-0.799311	2.133385	0.747970
C	-2.595822	1.892990	3.753428
H	-2.999415	1.059561	4.363662
H	-1.875707	2.460170	4.377688
H	-3.435447	2.577976	3.514961
C	0.015564	-0.820312	0.587977
C	-0.631670	-1.916599	2.500319
H	-1.338851	-2.284001	3.249977
C	0.728725	-2.042526	2.397128
H	1.461303	-2.541393	3.038569
C	2.452362	-1.201856	0.781121
C	2.997756	-2.149131	-0.119205
C	4.326615	-1.953641	-0.541368
H	4.779399	-2.666241	-1.247180
C	5.076320	-0.861359	-0.082164
H	6.114862	-0.727935	-0.422333
C	4.507171	0.064652	0.802186
H	5.101202	0.924101	1.149226
C	3.179663	-0.081831	1.250429
C	2.155532	-3.287074	-0.680921
H	1.264480	-3.393377	-0.028847
C	2.889667	-4.635675	-0.680575
H	3.253126	-4.900344	0.333094
H	2.208713	-5.440880	-1.023619
H	3.763123	-4.631172	-1.364998
C	1.651325	-2.911548	-2.087462
H	2.500892	-2.799816	-2.793377
H	0.970183	-3.692806	-2.483512
H	1.096032	-1.945315	-2.051659

```
  C       2.564759      0.947605      2.190334
  H       1.507156      0.662133      2.359443
  C       2.552771      2.347209      1.554033
  H       2.007663      2.336178      0.589002
  H       2.046649      3.073018      2.222733
  H       3.580814      2.721281      1.367290
  C       3.267965      0.944694      3.558156
  H       4.335704      1.232995      3.464223
  H       2.782719      1.667964      4.245211
  H       3.227022     -0.058498      4.029168
  C       0.068807      3.582786     -1.641951
  C       1.024595      2.675821     -2.108155
  C      -1.312308      3.344250     -1.811576
  C       0.602940      1.503781     -2.808166
  H       2.096240      2.881142     -1.976458
  C      -1.726840      2.167728     -2.440579
  C      -0.791914      1.226744     -2.984674
  H       1.357083      0.917059     -3.357955
  H      -2.803857      1.975562     -2.569493
  C      -1.273899      0.169467     -3.952896
  H      -1.471989      0.620097     -4.950051
  H      -0.527013     -0.636051     -4.087333
  H      -2.219560     -0.288285     -3.601982
  Cl      0.587577      5.004548     -0.750464
  H      -2.045622      4.061742     -1.417976
  Pd     -0.006618      0.257768     -1.113066
I1_2-methyl-1-butanol.xyz
81
Coordinates from ORCA-job I1 E -2016.089042255462
  N      -1.041587     -1.168644      1.395882
  C      -2.389092     -0.748296      1.126778
  N       1.098201     -1.369080      1.230198
  C      -3.212995     -1.579993      0.331031
  C      -4.499866     -1.104323      0.012307
  H      -5.164198     -1.718270     -0.614518
  C      -4.942633      0.141789      0.478000
  H      -5.949569      0.499358      0.212560
  C      -4.113531      0.931598      1.287081
  H      -4.477122      1.904733      1.650854
  C      -2.818466      0.501722      1.636032
  C      -2.715910     -2.916927     -0.205311
  H      -1.801279     -3.183606      0.362869
  C      -2.310316     -2.787029     -1.684178
  H      -1.538374     -1.997491     -1.808154
  H      -1.895447     -3.744475     -2.061861
  H      -3.185249     -2.518182     -2.312622
  C      -3.733538     -4.048951      0.003566
  H      -4.654492     -3.887519     -0.593697
  H      -3.298234     -5.017066     -0.317434
  H      -4.028315     -4.139285      1.068681
```

C	-1.907613	1.377908	2.488497
H	-1.048706	0.753822	2.810147
C	-1.338451	2.540056	1.656080
H	-2.148785	3.213895	1.307962
H	-0.623874	3.141058	2.255101
H	-0.806478	2.152522	0.761166
C	-2.605699	1.879991	3.761927
H	-3.003771	1.039258	4.365699
H	-1.889431	2.447048	4.390731
H	-3.449747	2.560821	3.527148
C	0.015097	-0.816836	0.587852
C	-0.630532	-1.912737	2.500406
H	-1.337089	-2.280519	3.250618
C	0.729945	-2.038129	2.396391
H	1.463067	-2.536795	3.037544
C	2.451098	-1.198034	0.776156
C	2.992223	-2.144208	-0.127930
C	4.318422	-1.946685	-0.558305
H	4.767719	-2.658612	-1.267203
C	5.069635	-0.853752	-0.102708
H	6.105954	-0.718750	-0.449096
C	4.505126	0.070517	0.786921
H	5.100963	0.929446	1.132258
C	3.180390	-0.077980	1.243080
C	2.150909	-3.286492	-0.682265
H	1.253890	-3.381998	-0.036978
C	2.882740	-4.636245	-0.655481
H	3.229998	-4.888890	0.366890
H	2.205354	-5.444340	-0.998844
H	3.766611	-4.640241	-1.326422
C	1.660532	-2.931275	-2.098797
H	2.516095	-2.832218	-2.799354
H	0.981396	-3.717424	-2.488535
H	1.106479	-1.964223	-2.083627
C	2.571086	0.947210	2.191388
H	1.512890	0.664236	2.361392
C	2.563603	2.352631	1.567575
H	2.018579	2.353625	0.602224
H	2.060153	3.073429	2.243765
H	3.592874	2.724882	1.384154
C	3.277830	0.930889	3.557404
H	4.346395	1.215678	3.462038
H	2.797015	1.650819	4.251047
H	3.233594	-0.075818	4.020494
C	0.063384	3.586450	-1.642814
C	1.022932	2.682443	-2.107256
C	-1.316850	3.344661	-1.815099
C	0.605757	1.509398	-2.808485
H	2.093927	2.890788	-1.974047
C	-1.726749	2.167474	-2.446555

```
  C      -0.787934      1.229275     -2.989487
  H       1.362672      0.924897     -3.356912
  H      -2.803030      1.974068     -2.580469
  C      -1.264672      0.171806     -3.960041
  H      -1.462800      0.623958     -4.956437
  H      -0.514402     -0.630405     -4.095376
  H      -2.209713     -0.289547     -3.612050
  Cl      0.576961      5.009796     -0.749405
  H      -2.053448      4.059817     -1.423131
  Pd     -0.009747      0.261187     -1.114533
I1_t-BuOH.xyz
81
Coordinates from ORCA-job I1 E -2016.089159087103
  N      -1.041547     -1.168667      1.395928
  C      -2.389095     -0.748341      1.126986
  N       1.098226     -1.368963      1.229874
  C      -3.213001     -1.579956      0.331153
  C      -4.499835     -1.104195      0.012384
  H      -5.164160     -1.718068     -0.614521
  C      -4.942572      0.141899      0.478160
  H      -5.949474      0.499540      0.212687
  C      -4.113496      0.931588      1.287399
  H      -4.477095      1.904679      1.651277
  C      -2.818461      0.501636      1.636380
  C      -2.716032     -2.916970     -0.205111
  H      -1.801445     -3.183717      0.363107
  C      -2.310435     -2.787307     -1.683998
  H      -1.538340     -1.997937     -1.808119
  H      -1.895745     -3.744879     -2.061556
  H      -3.185332     -2.518398     -2.312468
  C      -3.733792     -4.048863      0.003865
  H      -4.654720     -3.887374     -0.593422
  H      -3.298578     -5.017051     -0.317040
  H      -4.028570     -4.139061      1.068991
  C      -1.907576      1.377699      2.488950
  H      -1.049149      0.753293      2.811274
  C      -1.337361      2.539225      1.656358
  H      -2.147133      3.213402      1.307586
  H      -0.622715      3.140026      2.255502
  H      -0.805214      2.151012      0.761825
  C      -2.605971      1.880692      3.761845
  H      -3.004883      1.040438      4.365727
  H      -1.889631      2.447502      4.390789
  H      -3.449453      2.562002      3.526420
  C       0.014974     -0.816835      0.587712
  C      -0.630241     -1.912707      2.500397
  H      -1.336637     -2.280531      3.250745
  C       0.730231     -2.038013      2.396143
  H       1.463493     -2.536640      3.037170
  C       2.450977     -1.197678      0.775504
```

```
  C       2.992140     -2.143884     -0.128513
  C       4.318186     -1.946073     -0.559259
  H       4.767517     -2.658033     -1.268112
  C       5.069195     -0.852827     -0.104070
  H       6.105404     -0.717619     -0.450706
  C       4.504625      0.071499      0.785482
  H       5.100335      0.930637      1.130517
  C       3.180023     -0.077249      1.241946
  C       2.151107     -3.286631     -0.682314
  H       1.253813     -3.381687     -0.037355
  C       2.883087     -4.636276     -0.654031
  H       3.229585     -4.888126      0.368794
  H       2.206069     -5.444712     -0.997323
  H       3.767484     -4.640597     -1.324277
  C       1.661318     -2.932731     -2.099370
  H       2.517129     -2.834244     -2.799704
  H       0.982419     -3.719311     -2.488651
  H       1.107160     -1.965749     -2.085409
  C       2.570691      0.947705      2.190494
  H       1.511971      0.665710      2.358871
  C       2.565532      2.353901      1.568443
  H       2.021813      2.356764      0.602362
  H       2.061908      3.074313      2.244916
  H       3.595402      2.725410      1.386927
  C       3.275781      0.928921      3.557353
  H       4.344833      1.212387      3.463488
  H       2.795094      1.648691      4.251256
  H       3.229662     -0.078258      4.019228
  C       0.062718      3.586646     -1.642555
  C       1.022496      2.682864     -2.106966
  C      -1.317454      3.344632     -1.815037
  C       0.605591      1.509811     -2.808331
  H       2.093445      2.891406     -1.973679
  C      -1.727067      2.167440     -2.446684
  C      -0.788021      1.229447     -2.989602
  H       1.362664      0.925484     -3.356731
  H      -2.803300      1.973915     -2.580834
  C      -1.264440      0.172005     -3.960341
  H      -1.462539      0.624255     -4.956697
  H      -0.513990     -0.630032     -4.095702
  H      -2.209444     -0.289565     -3.612533
  Cl      0.575931      5.010044     -0.748987
  H      -2.054242      4.059626     -1.423122
  Pd     -0.010082      0.261283     -1.114611
I1_Heptane.xyz
81
Coordinates from ORCA-job I1 E -2016.080402476668
  N      -1.042347     -1.176026      1.395148
  C      -2.390344     -0.754263      1.129994
  N       1.097899     -1.380591      1.233332
```

C	-3.220322	-1.589587	0.345089
C	-4.512510	-1.120271	0.042411
H	-5.182085	-1.737772	-0.574950
C	-4.954246	0.124389	0.511168
H	-5.966086	0.477077	0.258557
C	-4.117572	0.919413	1.305758
H	-4.479386	1.892710	1.670274
C	-2.817057	0.495785	1.639507
C	-2.717884	-2.918795	-0.204706
H	-1.812891	-3.195961	0.374314
C	-2.284264	-2.757454	-1.672547
H	-1.518162	-1.957461	-1.763944
H	-1.854764	-3.703507	-2.062545
H	-3.149787	-2.482517	-2.311277
C	-3.737553	-4.054990	-0.036137
H	-4.645451	-3.887514	-0.651509
H	-3.295068	-5.017897	-0.362917
H	-4.058210	-4.163308	1.019766
C	-1.896614	1.379389	2.473252
H	-1.041683	0.753094	2.802061
C	-1.321915	2.520105	1.616079
H	-2.129765	3.188964	1.253794
H	-0.605448	3.132716	2.200528
H	-0.792455	2.111501	0.728889
C	-2.583842	1.910142	3.740752
H	-2.993962	1.086101	4.359320
H	-1.858984	2.477839	4.358956
H	-3.418206	2.600247	3.498712
C	0.017111	-0.823063	0.589368
C	-0.634746	-1.924039	2.498467
H	-1.343612	-2.291909	3.245981
C	0.725398	-2.051999	2.396926
H	1.456255	-2.553536	3.037873
C	2.454620	-1.210147	0.789969
C	3.004279	-2.158500	-0.106377
C	4.338048	-1.969362	-0.514012
H	4.794790	-2.683389	-1.215542
C	5.088100	-0.881302	-0.046578
H	6.130677	-0.752758	-0.375857
C	4.513751	0.047380	0.830614
H	5.107021	0.905568	1.181863
C	3.181596	-0.093217	1.265671
C	2.159024	-3.286935	-0.682364
H	1.271686	-3.403672	-0.026655
C	2.890766	-4.636293	-0.711949
H	3.269990	-4.917179	0.291509
H	2.204374	-5.435875	-1.057201
H	3.753531	-4.624104	-1.409688
C	1.646007	-2.883695	-2.078076
H	2.491924	-2.756670	-2.785728

```
  H       0.963177     -3.656960     -2.486740
  H       1.091072     -1.917900     -2.018985
  C       2.559177      0.944836      2.190823
  H       1.505672      0.649953      2.368832
  C       2.525783      2.329182      1.523096
  H       1.971568      2.286345      0.564434
  H       2.017783      3.066340      2.177590
  H       3.547895      2.709719      1.317485
  C       3.266694      0.978395      3.555629
  H       4.330102      1.279917      3.454800
  H       2.773974      1.707903      4.230617
  H       3.242340     -0.015718      4.046620
  C       0.078986      3.582031     -1.638746
  C       1.027629      2.669947     -2.109116
  C      -1.303701      3.349015     -1.803324
  C       0.597735      1.499399     -2.806052
  H       2.100405      2.870430     -1.981169
  C      -1.726780      2.173628     -2.427287
  C      -0.799182      1.227354     -2.973066
  H       1.346539      0.908716     -3.358730
  H      -2.805087      1.983505     -2.546492
  C      -1.290931      0.169443     -3.935684
  H      -1.491054      0.616411     -4.934183
  H      -0.549605     -0.641360     -4.069163
  H      -2.236487     -0.282940     -3.577856
  Cl      0.607296      4.999904     -0.750281
  H      -2.030825      4.071063     -1.407067
  Pd      0.001958      0.258250     -1.106855
I1_n-BuOH.xyz
81
Coordinates from ORCA-job I1 E -2016.089270135082
  N      -1.041709     -1.168168      1.396245
  C      -2.389325     -0.748253      1.127034
  N       1.098052     -1.368381      1.230139
  C      -3.212894     -1.580212      0.331192
  C      -4.499781     -1.104774      0.012092
  H      -5.163868     -1.718917     -0.614805
  C      -4.942879      0.141323      0.477556
  H      -5.949818      0.498682      0.211836
  C      -4.114135      0.931352      1.286826
  H      -4.478038      1.904411      1.650500
  C      -2.819071      0.501720      1.636143
  C      -2.715564     -2.917251     -0.204693
  H      -1.801016     -3.183704      0.363723
  C      -2.309815     -2.787951     -1.683574
  H      -1.537817     -1.998513     -1.807892
  H      -1.894879     -3.745566     -2.060747
  H      -3.184682     -2.519419     -2.312248
  C      -3.733152     -4.049280      0.004418
  H      -4.654032     -3.888079     -0.593019
```

H	-3.297687	-5.017444	-0.316211
H	-4.027994	-4.139281	1.069543
C	-1.908651	1.377993	2.489009
H	-1.049832	0.753983	2.811031
C	-1.339206	2.540265	1.656931
H	-2.149464	3.213937	1.308299
H	-0.625133	3.141399	2.256432
H	-0.806575	2.152816	0.762360
C	-2.607359	1.880024	3.762118
H	-3.005668	1.039226	4.365640
H	-1.891366	2.447024	4.391285
H	-3.451311	2.560844	3.526956
C	0.014749	-0.816422	0.587953
C	-0.630319	-1.911951	2.500851
H	-1.336665	-2.279669	3.251306
C	0.730161	-2.037180	2.396577
H	1.463491	-2.535574	3.037719
C	2.450692	-1.197343	0.775367
C	2.991082	-2.143133	-0.129547
C	4.316847	-1.945240	-0.561175
H	4.765563	-2.656914	-1.270718
C	5.068334	-0.852334	-0.105946
H	6.104282	-0.717024	-0.453328
C	4.504590	0.071495	0.784674
H	5.100696	0.930318	1.129817
C	3.180324	-0.077392	1.242089
C	2.149854	-3.285909	-0.683010
H	1.251991	-3.379933	-0.038710
C	2.881314	-4.635816	-0.652679
H	3.226479	-4.886837	0.370799
H	2.204395	-5.444310	-0.996033
H	3.766515	-4.640971	-1.321858
C	1.661422	-2.933335	-2.100852
H	2.517832	-2.835701	-2.800570
H	0.982737	-3.720197	-2.489928
H	1.107371	-1.966297	-2.088335
C	2.571889	0.947028	2.191796
H	1.513535	0.664554	2.361693
C	2.565280	2.353400	1.570088
H	2.020470	2.356331	0.604603
H	2.062090	3.073376	2.247353
H	3.594773	2.725407	1.387439
C	3.278953	0.928327	3.557627
H	4.347715	1.212392	3.462288
H	2.798861	1.647708	4.252340
H	3.234019	-0.078971	4.019357
C	0.063751	3.586777	-1.643078
C	1.023411	2.682665	-2.107096
C	-1.316455	3.345152	-1.815815
C	0.606400	1.509580	-2.808420

```
  H       2.094390      2.890930     -1.973542
  C      -1.726220      2.167985     -2.447437
  C      -0.787261      1.229676     -2.989978
  H       1.363494      0.924986     -3.356503
  H      -2.802469      1.974800     -2.581977
  C      -1.263737      0.172174     -3.960634
  H      -1.461923      0.624402     -4.956978
  H      -0.513275     -0.629843     -4.096058
  H      -2.208738     -0.289385     -3.612784
  Cl      0.577196      5.010175     -0.749557
  H      -2.053169      4.060412     -1.424233
  Pd     -0.010291      0.261499     -1.114605
I1_MEK.xyz
81
Coordinates from ORCA-job I1 E -2016.089334057872
  N      -1.041531     -1.168252      1.396043
  C      -2.389108     -0.748146      1.126935
  N       1.098212     -1.368558      1.229797
  C      -3.212691     -1.579795      0.330788
  C      -4.499457     -1.104084      0.011588
  H      -5.163530     -1.717984     -0.615566
  C      -4.942443      0.141958      0.477296
  H      -5.949279      0.499548      0.211492
  C      -4.113722      0.931639      1.286939
  H      -4.477557      1.904643      1.650826
  C      -2.818770      0.501743      1.636341
  C      -2.715644     -2.916931     -0.205120
  H      -1.800736     -3.183240      0.362775
  C      -2.310863     -2.788079     -1.684297
  H      -1.538747     -1.998882     -1.809329
  H      -1.896414     -3.745890     -2.061506
  H      -3.186068     -2.519497     -2.312479
  C      -3.733139     -4.048870      0.004999
  H      -4.654461     -3.887714     -0.591768
  H      -3.297973     -5.017133     -0.315742
  H      -4.027174     -4.138608      1.070369
  C      -1.908316      1.377711      2.489483
  H      -1.049847      0.753397      2.811859
  C      -1.338093      2.539713      1.657535
  H      -2.147898      3.213867      1.308776
  H      -0.623774      3.140400      2.257191
  H      -0.805561      2.151982      0.763027
  C      -2.607235      1.880108      3.762328
  H      -3.006089      1.039517      4.365777
  H      -1.891207      2.446850      4.391689
  H      -3.450820      2.561277      3.526857
  C       0.014896     -0.816539      0.587694
  C      -0.630115     -1.912127      2.500575
  H      -1.336436     -2.279872      3.251042
  C       0.730353     -2.037427      2.396207
```

```
  H       1.463686     -2.535941      3.037252
  C       2.450860     -1.197389      0.775097
  C       2.991656     -2.143548     -0.129191
  C       4.317500     -1.945634     -0.560569
  H       4.766543     -2.657575     -1.269632
  C       5.068664     -0.852358     -0.105679
  H       6.104701     -0.717070     -0.452805
  C       4.504496      0.071862      0.784270
  H       5.100375      0.930946      1.129155
  C       3.180111     -0.076994      1.241357
  C       2.150631     -3.286599     -0.682385
  H       1.253064     -3.380994     -0.037723
  C       2.882509     -4.636279     -0.652543
  H       3.228255     -4.887300      0.370739
  H       2.205653     -5.444934     -0.995637
  H       3.767371     -4.641126     -1.322171
  C       1.661486     -2.934027     -2.099989
  H       2.517565     -2.836340     -2.800109
  H       0.982663     -3.720930     -2.488743
  H       1.107392     -1.967017     -2.087253
  C       2.571252      0.947644      2.190558
  H       1.512464      0.665909      2.358980
  C       2.566526      2.354284      1.569485
  H       2.022908      2.358095      0.603335
  H       2.063055      3.074318      2.246477
  H       3.596511      2.725640      1.388315
  C       3.276583      0.927765      3.557285
  H       4.345721      1.210883      3.463336
  H       2.796277      1.647297      4.251696
  H       3.230143     -0.079683      4.018536
  C       0.062719      3.586897     -1.642658
  C       1.022648      2.683195     -2.106927
  C      -1.317419      3.344835     -1.815341
  C       0.605930      1.510116     -2.808389
  H       2.093574      2.891819     -1.973511
  C      -1.726851      2.167651     -2.447167
  C      -0.787638      1.229743     -2.989987
  H       1.363146      0.925854     -3.356662
  H      -2.803051      1.974183     -2.581705
  C      -1.263802      0.172344     -3.960899
  H      -1.461871      0.624724     -4.957196
  H      -0.513196     -0.629535     -4.096338
  H      -2.208803     -0.289397     -3.613297
  Cl      0.575750      5.010336     -0.748958
  H      -2.054346      4.059777     -1.423577
  Pd     -0.010415      0.261500     -1.114783
I1_EtOH.xyz
81
Coordinates from ORCA-job I1 E -2016.089659267689
  N      -1.041475     -1.167538      1.396058
```

C	-2.389024	-0.747658	1.126505
N	1.098250	-1.367811	1.229863
C	-3.212053	-1.579289	0.329729
C	-4.498545	-1.103451	0.009479
H	-5.162172	-1.717320	-0.618192
C	-4.941824	0.142636	0.474871
H	-5.948417	0.500308	0.208247
C	-4.113741	0.932203	1.285334
H	-4.477876	1.905152	1.649106
C	-2.819073	0.502189	1.635752
C	-2.715029	-2.916803	-0.205294
H	-1.799718	-3.182454	0.362234
C	-2.311396	-2.789670	-1.684931
H	-1.539140	-2.000894	-1.811584
H	-1.897422	-3.748018	-2.061285
H	-3.186995	-2.521666	-2.312812
C	-3.732314	-4.048595	0.006796
H	-4.654198	-3.887905	-0.589226
H	-3.297364	-5.017110	-0.313471
H	-4.025224	-4.137340	1.072558
C	-1.909496	1.377879	2.490135
H	-1.050392	0.754034	2.811685
C	-1.340406	2.541788	1.660062
H	-2.150814	3.215731	1.312255
H	-0.626736	3.142181	2.260799
H	-0.807353	2.155979	0.765058
C	-2.609138	1.877619	3.763648
H	-3.006697	1.035575	4.365917
H	-1.893887	2.444490	4.393779
H	-3.453739	2.557825	3.529030
C	0.014964	-0.815777	0.587819
C	-0.630108	-1.911552	2.500498
H	-1.336450	-2.279412	3.250910
C	0.730375	-2.036846	2.396154
H	1.463680	-2.535473	3.037167
C	2.450743	-1.196992	0.774618
C	2.990624	-2.142941	-0.130443
C	4.316114	-1.944999	-0.563013
H	4.764446	-2.656834	-1.272660
C	5.067799	-0.851919	-0.108464
H	6.103527	-0.716598	-0.456515
C	4.504596	0.071999	0.782475
H	5.100964	0.930798	1.127243
C	3.180615	-0.076915	1.240770
C	2.149509	-3.286399	-0.682689
H	1.250730	-3.378711	-0.039452
C	2.880544	-4.636459	-0.648266
H	3.223374	-4.885535	0.376469
H	2.204111	-5.445419	-0.991484
H	3.767197	-4.642975	-1.315507

```
  C       1.663182     -2.937063     -2.102038
  H       2.520471     -2.841583     -2.800979
  H       0.984666     -3.724624     -2.489989
  H       1.109484     -1.969855     -2.092873
  C       2.572847      0.947235      2.191220
  H       1.514328      0.665272      2.361013
  C       2.567262      2.354257      1.570925
  H       2.022637      2.358719      0.605309
  H       2.064494      3.073804      2.248972
  H       3.597032      2.725754      1.388833
  C       3.280014      0.926860      3.556994
  H       4.348948      1.210283      3.461681
  H       2.800436      1.645973      4.252338
  H       3.234341     -0.080820      4.017805
  C       0.062124      3.587673     -1.642666
  C       1.022433      2.684313     -2.106854
  C      -1.317930      3.345314     -1.815620
  C       0.606162      1.511157     -2.808490
  H       2.093299      2.893283     -1.973370
  C      -1.726895      2.168080     -2.447735
  C      -0.787293      1.230445     -2.990420
  H       1.363629      0.927179     -3.356732
  H      -2.803022      1.974532     -2.582846
  C      -1.262962      0.172966     -3.961478
  H      -1.461192      0.625494     -4.957667
  H      -0.511965     -0.628511     -4.097116
  H      -2.207840     -0.289193     -3.614080
  Cl      0.574668      5.011188     -0.748634
  H      -2.055199      4.060015     -1.424023
  Pd     -0.010345      0.262415     -1.114742
I1_ethyleneglycol.xyz
81
Coordinates from ORCA-job I1 E -2016.090006999274
  N      -1.041363     -1.166474      1.396375
  C      -2.388976     -0.747221      1.126230
  N       1.098313     -1.366732      1.229932
  C      -3.211008     -1.578997      0.328541
  C      -4.497282     -1.103361      0.006952
  H      -5.160124     -1.717330     -0.621467
  C      -4.941328      0.142585      0.472057
  H      -5.947711      0.500085      0.204393
  C      -4.114338      0.932172      1.283688
  H      -4.479180      1.904889      1.647395
  C      -2.819919      0.502384      1.635397
  C      -2.713587     -2.916787     -0.205472
  H      -1.797712     -3.181383      0.361614
  C      -2.311355     -2.791549     -1.685645
  H      -1.539189     -2.003022     -1.814191
  H      -1.897628     -3.750394     -2.060992
  H      -3.187504     -2.524515     -2.313169
```

C	-3.730269	-4.048720	0.008954
H	-4.652880	-3.888854	-0.586162
H	-3.295292	-5.017399	-0.310780
H	-4.021809	-4.136402	1.075177
C	-1.911567	1.377937	2.491254
H	-1.052509	0.754292	2.813300
C	-1.342090	2.542727	1.662636
H	-2.152357	3.216688	1.314497
H	-0.629232	3.142854	2.264622
H	-0.807983	2.157756	0.767901
C	-2.612792	1.876553	3.764338
H	-3.010440	1.033867	4.365643
H	-1.898447	2.443364	4.395549
H	-3.457537	2.556418	3.529239
C	0.014966	-0.814751	0.588060
C	-0.629872	-1.910512	2.500739
H	-1.336122	-2.278443	3.251225
C	0.730617	-2.035794	2.396236
H	1.463997	-2.534444	3.037172
C	2.450509	-1.196331	0.773724
C	2.989097	-2.142016	-0.132377
C	4.314058	-1.944016	-0.566674
H	4.761396	-2.655720	-1.277115
C	5.066435	-0.851141	-0.112734
H	6.101706	-0.715756	-0.462130
C	4.504555	0.072439	0.779470
H	5.101561	0.930938	1.123900
C	3.181171	-0.076562	1.239500
C	2.147835	-3.286050	-0.683239
H	1.247407	-3.375438	-0.041947
C	2.877681	-4.636618	-0.642466
H	3.216600	-4.882951	0.384228
H	2.201825	-5.446036	-0.985752
H	3.766735	-4.645485	-1.306479
C	1.665324	-2.941211	-2.104953
H	2.524244	-2.848693	-2.802286
H	0.987275	-3.729721	-2.491785
H	1.112123	-1.973784	-2.100680
C	2.574860	0.946912	2.191634
H	1.516377	0.665284	2.362287
C	2.569494	2.354761	1.573136
H	2.024289	2.360930	0.607823
H	2.067556	3.073597	2.252565
H	3.599323	2.725987	1.390852
C	3.283480	0.924634	3.556636
H	4.352448	1.207612	3.460381
H	2.804995	1.643243	4.253252
H	3.237708	-0.083535	4.016356
C	0.061717	3.588653	-1.642074
C	1.022170	2.685419	-2.106278

```
  C      -1.318311      3.346511     -1.815525
  C       0.606040      1.512479     -2.808419
  H       2.093033      2.894423     -1.972669
  C      -1.727129      2.169432     -2.448102
  C      -0.787386      1.231812     -2.990606
  H       1.363599      0.928718     -3.356778
  H      -2.803231      1.976163     -2.583909
  C      -1.262863      0.174319     -3.961731
  H      -1.461444      0.627040     -4.957752
  H      -0.511583     -0.626819     -4.097766
  H      -2.207585     -0.288180     -3.614342
  Cl      0.574155      5.011891     -0.747358
  H      -2.055724      4.061214     -1.424167
  Pd     -0.010256      0.263837     -1.114417
start.xyz
81
Coordinates from ORCA-job I1 E -2016.080402476668
  N      -1.042347     -1.176026      1.395148
  C      -2.390344     -0.754263      1.129994
  N       1.097899     -1.380591      1.233332
  C      -3.220322     -1.589587      0.345089
  C      -4.512510     -1.120271      0.042411
  H      -5.182085     -1.737772     -0.574950
  C      -4.954246      0.124389      0.511168
  H      -5.966086      0.477077      0.258557
  C      -4.117572      0.919413      1.305758
  H      -4.479386      1.892710      1.670274
  C      -2.817057      0.495785      1.639507
  C      -2.717884     -2.918795     -0.204706
  H      -1.812891     -3.195961      0.374314
  C      -2.284264     -2.757454     -1.672547
  H      -1.518162     -1.957461     -1.763944
  H      -1.854764     -3.703507     -2.062545
  H      -3.149787     -2.482517     -2.311277
  C      -3.737553     -4.054990     -0.036137
  H      -4.645451     -3.887514     -0.651509
  H      -3.295068     -5.017897     -0.362917
  H      -4.058210     -4.163308      1.019766
  C      -1.896614      1.379389      2.473252
  H      -1.041683      0.753094      2.802061
  C      -1.321915      2.520105      1.616079
  H      -2.129765      3.188964      1.253794
  H      -0.605448      3.132716      2.200528
  H      -0.792455      2.111501      0.728889
  C      -2.583842      1.910142      3.740752
  H      -2.993962      1.086101      4.359320
  H      -1.858984      2.477839      4.358956
  H      -3.418206      2.600247      3.498712
  C       0.017111     -0.823063      0.589368
  C      -0.634746     -1.924039      2.498467
```

H	-1.343612	-2.291909	3.245981
C	0.725398	-2.051999	2.396926
H	1.456255	-2.553536	3.037873
C	2.454620	-1.210147	0.789969
C	3.004279	-2.158500	-0.106377
C	4.338048	-1.969362	-0.514012
H	4.794790	-2.683389	-1.215542
C	5.088100	-0.881302	-0.046578
H	6.130677	-0.752758	-0.375857
C	4.513751	0.047380	0.830614
H	5.107021	0.905568	1.181863
C	3.181596	-0.093217	1.265671
C	2.159024	-3.286935	-0.682364
H	1.271686	-3.403672	-0.026655
C	2.890766	-4.636293	-0.711949
H	3.269990	-4.917179	0.291509
H	2.204374	-5.435875	-1.057201
H	3.753531	-4.624104	-1.409688
C	1.646007	-2.883695	-2.078076
H	2.491924	-2.756670	-2.785728
H	0.963177	-3.656960	-2.486740
H	1.091072	-1.917900	-2.018985
C	2.559177	0.944836	2.190823
H	1.505672	0.649953	2.368832
C	2.525783	2.329182	1.523096
H	1.971568	2.286345	0.564434
H	2.017783	3.066340	2.177590
H	3.547895	2.709719	1.317485
C	3.266694	0.978395	3.555629
H	4.330102	1.279917	3.454800
H	2.773974	1.707903	4.230617
H	3.242340	-0.015718	4.046620
C	0.078986	3.582031	-1.638746
C	1.027629	2.669947	-2.109116
C	-1.303701	3.349015	-1.803324
C	0.597735	1.499399	-2.806052
H	2.100405	2.870430	-1.981169
C	-1.726780	2.173628	-2.427287
C	-0.799182	1.227354	-2.973066
H	1.346539	0.908716	-3.358730
H	-2.805087	1.983505	-2.546492
C	-1.290931	0.169443	-3.935684
H	-1.491054	0.616411	-4.934183
H	-0.549605	-0.641360	-4.069163
H	-2.236487	-0.282940	-3.577856
Cl	0.607296	4.999904	-0.750281
H	-2.030825	4.071063	-1.407067
Pd	0.001958	0.258250	-1.106855

I1_IsoamylAlcohol.xyz
81

Coordinates from ORCA-job I1 E -2016.089042403433

N	-1.041647	-1.168677	1.396135
C	-2.389260	-0.748521	1.127227
N	1.098122	-1.369074	1.230109
C	-3.213187	-1.580451	0.331740
C	-4.500228	-1.105066	0.013277
H	-5.164591	-1.719205	-0.613321
C	-4.943128	0.141000	0.478955
H	-5.950200	0.498342	0.213725
C	-4.113992	0.931048	1.287765
H	-4.477699	1.904131	1.651544
C	-2.818771	0.501455	1.636484
C	-2.715893	-2.917286	-0.204670
H	-1.801574	-3.184144	0.363935
C	-2.309491	-2.786991	-1.683286
H	-1.537526	-1.997367	-1.806666
H	-1.894363	-3.744318	-2.060988
H	-3.184116	-2.518032	-2.312114
C	-3.733621	-4.049376	0.003311
H	-4.654161	-3.887913	-0.594581
H	-3.298041	-5.017410	-0.317558
H	-4.029142	-4.139920	1.068203
C	-1.907835	1.377863	2.488626
H	-1.049625	0.753424	2.811491
C	-1.337207	2.538660	1.655301
H	-2.146763	3.212883	1.306115
H	-0.622371	3.139615	2.254060
H	-0.805161	2.149690	0.761024
C	-2.606221	1.881911	3.761102
H	-3.005616	1.042211	4.365437
H	-1.889737	2.448791	4.389817
H	-3.449358	2.563470	3.525157
C	0.014922	-0.816726	0.588022
C	-0.630420	-1.912990	2.500458
H	-1.336867	-2.280919	3.250703
C	0.730043	-2.038338	2.396236
H	1.463267	-2.537087	3.037207
C	2.450960	-1.197861	0.775949
C	2.992056	-2.143860	-0.128335
C	4.318249	-1.946268	-0.558696
H	4.767524	-2.658062	-1.267746
C	5.069488	-0.853444	-0.102887
H	6.105814	-0.718405	-0.449238
C	4.504991	0.070681	0.786900
H	5.100847	0.929542	1.132376
C	3.180243	-0.077853	1.243009
C	2.150752	-3.286053	-0.682873
H	1.253566	-3.381470	-0.037808
C	2.882463	-4.635868	-0.655884
H	3.229348	-4.888570	0.366600

```
  H       2.205135     -5.443904     -0.999504
  H       3.766571     -4.639908     -1.326512
  C       1.660743     -2.930827     -2.099524
  H       2.516462     -2.831814     -2.799895
  H       0.981658     -3.716949     -2.489404
  H       1.106722     -1.963758     -2.084510
  C       2.570916      0.947203      2.191446
  H       1.512601      0.664412      2.361017
  C       2.563996      2.352829      1.568096
  H       2.019232      2.354317      0.602605
  H       2.060630      3.073551      2.244428
  H       3.593423      2.724843      1.385080
  C       3.277256      0.930249      3.557666
  H       4.345933      1.214747      3.462685
  H       2.796458      1.650113      4.251391
  H       3.232568     -0.076578      4.020450
  C       0.063649      3.586488     -1.642828
  C       1.023056      2.682190     -2.107003
  C      -1.316606      3.345134     -1.815543
  C       0.605702      1.509248     -2.808279
  H       2.094079      2.890263     -1.973608
  C      -1.726695      2.167986     -2.446947
  C      -0.788025      1.229406     -2.989472
  H       1.362566      0.924538     -3.356556
  H      -2.802998      1.974923     -2.581174
  C      -1.264849      0.171884     -3.959929
  H      -1.462808      0.623923     -4.956409
  H      -0.514712     -0.630483     -4.095072
  H      -2.210006     -0.289256     -3.611962
  Cl      0.577408      5.009690     -0.749297
  H      -2.053090      4.060649     -1.424013
  Pd     -0.010059      0.261368     -1.114305
I1_Ethylacetate.xyz
81
Coordinates from ORCA-job I1 E -2016.086946152313
  N      -1.041475     -1.171308      1.395335
  C      -2.388988     -0.750062      1.127461
  N       1.098459     -1.372716      1.230943
  C      -3.215150     -1.582481      0.334990
  C      -4.503328     -1.107925      0.020764
  H      -5.169526     -1.722539     -0.603331
  C      -4.945080      0.138149      0.487112
  H      -5.953188      0.494921      0.225167
  C      -4.113339      0.929104      1.292000
  H      -4.475817      1.902592      1.655773
  C      -2.816951      0.500255      1.636718
  C      -2.717248     -2.917597     -0.204943
  H      -1.805809     -3.187840      0.366812
  C      -2.302901     -2.778978     -1.680608
  H      -1.531659     -1.987204     -1.794917
```

H	-1.884739	-3.733625	-2.061782
H	-3.174656	-2.507349	-2.312261
C	-3.736430	-4.050200	-0.008152
H	-4.653175	-3.886484	-0.611255
H	-3.299419	-5.017113	-0.330464
H	-4.039328	-4.145417	1.054261
C	-1.902757	1.378126	2.483703
H	-1.045791	0.752690	2.808126
C	-1.330357	2.532972	1.643565
H	-2.138986	3.206627	1.291386
H	-0.613944	3.136210	2.238038
H	-0.800000	2.138114	0.750813
C	-2.597644	1.890106	3.754881
H	-3.000048	1.054919	4.363488
H	-1.878479	2.457181	4.380338
H	-3.438257	2.574168	3.517232
C	0.015983	-0.819336	0.587927
C	-0.631397	-1.916042	2.499866
H	-1.338630	-2.283644	3.249413
C	0.729013	-2.042011	2.396697
H	1.461525	-2.541131	3.038060
C	2.452549	-1.201338	0.780368
C	2.997211	-2.148585	-0.120452
C	4.325710	-1.952951	-0.543878
H	4.777891	-2.665544	-1.250120
C	5.075767	-0.860612	-0.085264
H	6.114002	-0.727096	-0.426362
C	4.507423	0.065255	0.799865
H	5.101854	0.924531	1.146675
C	3.180289	-0.081365	1.249292
C	2.155035	-3.287184	-0.680936
H	1.262912	-3.391557	-0.030064
C	2.888614	-4.636107	-0.676037
H	3.249042	-4.898822	0.339221
H	2.208173	-5.441658	-1.019293
H	3.764002	-4.633158	-1.357999
C	1.653363	-2.915062	-2.089269
H	2.504047	-2.805739	-2.794221
H	0.972430	-3.697042	-2.484243
H	1.098457	-1.948565	-2.057103
C	2.566380	0.947414	2.190600
H	1.508543	0.662678	2.359553
C	2.555858	2.348165	1.556701
H	2.011257	2.339607	0.591303
H	2.050072	3.073093	2.226636
H	3.584319	2.721653	1.371128
C	3.269850	0.941776	3.558305
H	4.337844	1.229140	3.464388
H	2.785402	1.664540	4.246455
H	3.227840	-0.062088	4.027775

```
  C       0.065678      3.583751     -1.642163
  C       1.022390      2.677717     -2.108394
  C      -1.315224      3.344233     -1.812065
  C       0.601830      1.505457     -2.808771
  H       2.093884      2.884047     -1.976798
  C      -1.728626      2.167424     -2.441447
  C      -0.792774      1.227214     -2.985384
  H       1.356523      0.919606     -3.358756
  H      -2.805470      1.974683     -2.571115
  C      -1.273656      0.169383     -3.953530
  H      -1.472231      0.619922     -4.950614
  H      -0.525826     -0.635230     -4.088097
  H      -2.218922     -0.289299     -3.602756
  Cl      0.583284      5.005829     -0.750147
  H      -2.049336      4.061042     -1.418649
  Pd     -0.006384      0.259380     -1.113018
TS1_THF.xyz
81
Coordinates from ORCA-job TS1 E -2016.072517915772
  Pd      0.757845      1.230894     -0.815203
  N      -0.737404     -0.597530      1.154546
  C      -2.028665     -0.113398      0.749107
  N       1.397086     -0.876443      1.296334
  C      -2.813492     -0.920887     -0.114121
  C      -4.060384     -0.410692     -0.521080
  H      -4.693260     -1.003395     -1.197141
  C      -4.506338      0.843975     -0.080325
  H      -5.482390      1.226422     -0.416629
  C      -3.720466      1.607388      0.790741
  H      -4.088098      2.584177      1.141145
  C      -2.464750      1.143313      1.231933
  C      -2.303326     -2.269417     -0.613827
  H      -1.663527     -2.696674      0.186150
  C      -1.413604     -2.089275     -1.857294
  H      -0.581001     -1.382863     -1.664340
  H      -0.979739     -3.061600     -2.169934
  H      -2.002973     -1.683414     -2.704379
  C      -3.424564     -3.281379     -0.882994
  H      -4.051178     -2.982405     -1.748745
  H      -2.988688     -4.271724     -1.124092
  H      -4.090152     -3.401911     -0.004427
  C      -1.641813      1.972571      2.210421
  H      -0.716811      1.406181      2.440125
  C      -1.202382      3.307954      1.589129
  H      -2.073538      3.930557      1.297074
  H      -0.592718      3.888834      2.311272
  H      -0.581991      3.120068      0.685685
  C      -2.398871      2.180945      3.533414
  H      -2.687953      1.212592      3.990032
  H      -1.762967      2.728057      4.259280
```

H	-3.323225	2.776038	3.381915
C	0.448110	-0.153107	0.611681
C	-0.528543	-1.554913	2.146202
H	-1.356618	-2.012699	2.695748
C	0.826268	-1.732887	2.235738
H	1.431612	-2.379735	2.877736
C	2.811161	-0.716061	1.095307
C	3.444006	-1.469959	0.078355
C	4.828246	-1.282016	-0.102413
H	5.350752	-1.846059	-0.890171
C	5.545407	-0.384152	0.700150
H	6.626637	-0.250444	0.541552
C	4.891844	0.345892	1.702784
H	5.464524	1.052484	2.322647
C	3.509356	0.194981	1.924846
C	2.661621	-2.407178	-0.831843
H	1.641273	-2.508734	-0.409929
C	3.279773	-3.812306	-0.894931
H	3.370881	-4.260119	0.115394
H	2.647941	-4.481961	-1.513362
H	4.290468	-3.794338	-1.352426
C	2.512108	-1.784502	-2.231532
H	3.500941	-1.649924	-2.717791
H	1.890356	-2.431128	-2.884454
H	2.020270	-0.788959	-2.157520
C	2.793763	1.031888	2.978130
H	1.774232	0.614920	3.104986
C	2.629383	2.482094	2.487505
H	2.083850	2.507043	1.519731
H	2.056333	3.080976	3.225320
H	3.616944	2.967702	2.341769
C	3.487834	0.970431	4.347192
H	4.500496	1.422577	4.313987
H	2.899474	1.532560	5.100714
H	3.592811	-0.075050	4.701670
C	-0.158820	2.009865	-2.645072
C	-0.034681	1.052944	-3.678024
C	-1.422017	2.551765	-2.311807
C	-1.200254	0.542453	-4.266175
H	0.957568	0.699202	-3.991129
C	-2.565204	2.014257	-2.915728
C	-2.483863	0.993304	-3.889215
H	-1.105380	-0.236541	-5.039593
H	-3.552051	2.399012	-2.614366
C	-3.730442	0.379004	-4.470629
H	-3.538969	-0.081841	-5.459597
H	-4.117283	-0.423661	-3.803351
H	-4.543913	1.123764	-4.580524
Cl	1.376739	3.076919	-2.284389
H	-1.503610	3.355280	-1.566944

```
TS1_IsoamylAlcohol.xyz
81
Coordinates from ORCA-job TS1 E -2016.074058041071
  Pd      0.754989      1.233833     -0.817548
  N      -0.737293     -0.596918      1.153849
  C      -2.029152     -0.113896      0.748689
  N       1.397507     -0.872976      1.295126
  C      -2.812682     -0.920983     -0.116192
  C      -4.059049     -0.410352     -0.524678
  H      -4.690644     -1.002206     -1.202726
  C      -4.506099      0.843642     -0.082849
  H      -5.481564      1.226394     -0.420505
  C      -3.722269      1.605827      0.791322
  H      -4.091184      2.581644      1.143130
  C      -2.466801      1.141685      1.233390
  C      -2.303193     -2.270296     -0.614683
  H      -1.660895     -2.695612      0.184231
  C      -1.417513     -2.093481     -1.861540
  H      -0.581767     -1.389931     -1.671738
  H      -0.987367     -3.067256     -2.174698
  H      -2.008819     -1.686988     -2.707092
  C      -3.425276     -3.282967     -0.878016
  H      -4.054724     -2.985930     -1.742342
  H      -2.989823     -4.273783     -1.117921
  H      -4.087706     -3.401295      0.003215
  C      -1.645771      1.969585      2.214672
  H      -0.719495      1.404633      2.442896
  C      -1.210162      3.308626      1.598339
  H      -2.083247      3.930561      1.310754
  H      -0.600501      3.887529      2.322105
  H      -0.591411      3.126824      0.692549
  C      -2.403620      2.171532      3.538244
  H      -2.689617      1.200705      3.991513
  H      -1.769251      2.718087      4.265860
  H      -3.329635      2.764384      3.388127
  C       0.447331     -0.151508      0.610615
  C      -0.526890     -1.553441      2.145924
  H      -1.354243     -2.012310      2.695759
  C       0.828225     -1.729648      2.235126
  H       1.434601     -2.375540      2.877193
  C       2.811007     -0.711100      1.091633
  C       3.442933     -1.466098      0.074799
  C       4.826134     -1.274609     -0.110932
  H       5.347953     -1.839970     -0.898270
  C       5.543105     -0.372064      0.686758
  H       6.623429     -0.235490      0.524323
  C       4.890646      0.358216      1.690092
  H       5.463332      1.067494      2.306962
  C       3.509305      0.203377      1.917375
  C       2.661461     -2.411175     -0.828115
```

```
  H       1.639268     -2.505636     -0.409205
  C       3.277263     -3.818243     -0.871414
  H       3.361383     -4.254238      0.144653
  H       2.647685     -4.493816     -1.485695
  H       4.290683     -3.806557     -1.323044
  C       2.519535     -1.806372     -2.236326
  H       3.510458     -1.683174     -2.721332
  H       1.897215     -2.459188     -2.882480
  H       2.031900     -0.808008     -2.178531
  C       2.796499      1.036909      2.975352
  H       1.774892      0.624110      3.098759
  C       2.639919      2.491978      2.496822
  H       2.092074      2.528464      1.530831
  H       2.071837      3.087943      3.240761
  H       3.629909      2.973228      2.353223
  C       3.490083      0.961114      4.344141
  H       4.505757      1.406641      4.313563
  H       2.905219      1.521628      5.101577
  H       3.587735     -0.087667      4.690823
  C      -0.158279      2.010770     -2.647083
  C      -0.034783      1.052908     -3.679173
  C      -1.419913      2.557919     -2.316989
  C      -1.201010      0.545635     -4.269056
  H       0.956780      0.695290     -3.990234
  C      -2.563924      2.023535     -2.922808
  C      -2.483991      1.001051     -3.894860
  H      -1.107194     -0.234540     -5.041398
  H      -3.550116      2.412069     -2.624031
  C      -3.731123      0.389396     -4.477728
  H      -3.541698     -0.062588     -5.471164
  H      -4.113345     -0.420142     -3.816161
  H      -4.546471      1.133358     -4.577808
  Cl      1.382109      3.077089     -2.285798
  H      -1.500650      3.362378     -1.572910
TS1_water.xyz
81
Coordinates from ORCA-job TS1 E -2016.075413139180
  Pd      0.752201      1.236395     -0.819886
  N      -0.737008     -0.596333      1.153255
  C      -2.029452     -0.114111      0.748646
  N       1.398131     -0.869906      1.293756
  C      -2.812275     -0.920744     -0.117413
  C      -4.058176     -0.409456     -0.526910
  H      -4.689007     -1.000501     -1.206406
  C      -4.505788      0.844090     -0.084157
  H      -5.480774      1.227260     -0.422720
  C      -3.723340      1.605087      0.792438
  H      -4.093080      2.580144      1.145527
  C      -2.468110      1.140596      1.235032
  C      -2.303975     -2.271016     -0.614755
```

H	-1.660730	-2.695462	0.183794
C	-1.420372	-2.097573	-1.863600
H	-0.581350	-1.397483	-1.675513
H	-0.994174	-3.072946	-2.177079
H	-2.012050	-1.689614	-2.708274
C	-3.427359	-3.283239	-0.874597
H	-4.057895	-2.987064	-1.738393
H	-2.992843	-4.274772	-1.113192
H	-4.088236	-3.399289	0.008081
C	-1.648474	1.967144	2.218662
H	-0.721295	1.403146	2.445674
C	-1.215866	3.309181	1.606527
H	-2.090509	3.930768	1.323048
H	-0.605805	3.886189	2.331493
H	-0.598862	3.132388	0.698562
C	-2.406882	2.163830	3.542738
H	-2.690422	1.190997	3.993205
H	-1.773647	2.709798	4.271775
H	-3.334183	2.754968	3.393828
C	0.446792	-0.149857	0.609738
C	-0.525106	-1.552453	2.145317
H	-1.351736	-2.012448	2.695394
C	0.830286	-1.727123	2.234020
H	1.437625	-2.372377	2.875890
C	2.811112	-0.706380	1.088129
C	3.442354	-1.461472	0.070811
C	4.824470	-1.266049	-0.119531
H	5.345784	-1.831781	-0.906991
C	5.541048	-0.359671	0.674348
H	6.620454	-0.219991	0.508359
C	4.889536	0.369665	1.679110
H	5.462089	1.080967	2.293838
C	3.509347	0.210658	1.911171
C	2.662023	-2.413576	-0.825775
H	1.638748	-2.503512	-0.408645
C	3.277321	-3.821404	-0.853387
H	3.357079	-4.247565	0.167178
H	2.649717	-4.502443	-1.463633
H	4.292528	-3.813781	-1.301079
C	2.524649	-1.823648	-2.240686
H	3.516784	-1.708492	-2.725174
H	1.902268	-2.482264	-2.880829
H	2.039191	-0.823775	-2.195907
C	2.798952	1.040003	2.974180
H	1.776000	0.629876	3.095082
C	2.647629	2.499366	2.507269
H	2.097287	2.546158	1.543228
H	2.083702	3.091710	3.257188
H	3.639200	2.977803	2.365294
C	3.492947	0.951488	4.342173

```
  H       4.510749      1.392276      4.313649
  H       2.910959      1.509425      5.103712
  H       3.585429     -0.100223      4.681251
  C      -0.158714      2.010613     -2.649439
  C      -0.035377      1.051984     -3.680731
  C      -1.419193      2.561743     -2.321996
  C      -1.201976      0.547023     -4.272133
  H       0.955710      0.691432     -3.990086
  C      -2.563673      2.029641     -2.929492
  C      -2.484553      1.005904     -3.900365
  H      -1.108780     -0.234109     -5.043566
  H      -3.549429      2.421081     -2.632980
  C      -3.732000      0.396354     -4.484609
  H      -3.543042     -0.052254     -5.479642
  H      -4.113077     -0.415663     -3.825480
  H      -4.547473      1.140589     -4.580927
  Cl      1.385502      3.077094     -2.287792
  H      -1.499535      3.366989     -1.578597
TS1_Acetone.xyz
81
Coordinates from ORCA-job TS1 E -2016.074520956961
  Pd      0.754010      1.234748     -0.818359
  N      -0.737224     -0.596745      1.153593
  C      -2.029293     -0.114021      0.748630
  N       1.397693     -0.871905      1.294607
  C      -2.812564     -0.920968     -0.116654
  C      -4.058787     -0.410141     -0.525476
  H      -4.690111     -1.001731     -1.204020
  C      -4.506055      0.843688     -0.083323
  H      -5.481364      1.226565     -0.421285
  C      -3.722711      1.605482      0.791679
  H      -4.091925      2.581032      1.143928
  C      -2.467310      1.141248      1.233918
  C      -2.303441     -2.270591     -0.614759
  H      -1.660777     -2.695575      0.184014
  C      -1.418512     -2.094899     -1.862321
  H      -0.581683     -1.392474     -1.673139
  H      -0.989662     -3.069202     -2.175580
  H      -2.009993     -1.687960     -2.707568
  C      -3.425934     -3.283161     -0.876852
  H      -4.055808     -2.986451     -1.740969
  H      -2.990766     -4.274203     -1.116326
  H      -4.087790     -3.400740      0.004904
  C      -1.646758      1.968704      2.215989
  H      -0.720186      1.404068      2.443830
  C      -1.212130      3.308738      1.601049
  H      -2.085726      3.930564      1.314817
  H      -0.602340      3.887007      2.325222
  H      -0.593954      3.128598      0.694535
  C      -2.404814      2.168917      3.539716
```

H	-2.690006	1.197426	3.992057
H	-1.770828	2.715279	4.267809
H	-3.331250	2.761207	3.389979
C	0.447107	-0.150950	0.610256
C	-0.526287	-1.553113	2.145677
H	-1.353384	-2.012378	2.695598
C	0.828926	-1.728760	2.234712
H	1.435650	-2.374405	2.876723
C	2.811010	-0.709410	1.090407
C	3.442775	-1.464536	0.073523
C	4.825615	-1.271702	-0.113720
H	5.347317	-1.837273	-0.901003
C	5.542389	-0.367728	0.682598
H	6.622408	-0.230090	0.518996
C	4.890171	0.362356	1.686273
H	5.462751	1.072436	2.302345
C	3.509210	0.206083	1.915128
C	2.661736	-2.412075	-0.827212
H	1.639141	-2.504934	-0.408970
C	3.277353	-3.819431	-0.865026
H	3.359894	-4.252017	0.152621
H	2.648495	-4.496901	-1.477952
H	4.291422	-3.809166	-1.315232
C	2.521476	-1.812431	-2.237778
H	3.512850	-1.692085	-2.722573
H	1.899128	-2.467239	-2.881874
H	2.034655	-0.813497	-2.184531
C	2.797186	1.038261	2.974739
H	1.774886	0.626812	3.096797
C	2.643236	2.495005	2.500516
H	2.094878	2.535531	1.535013
H	2.076588	3.089791	3.246472
H	3.634026	2.974902	2.357948
C	3.490393	0.957412	4.343496
H	4.507077	1.400709	4.314050
H	2.906591	1.517228	5.102266
H	3.585535	-0.092515	4.687375
C	-0.158423	2.010778	-2.647874
C	-0.034978	1.052628	-3.679669
C	-1.419665	2.559313	-2.318723
C	-1.201330	0.546147	-4.270079
H	0.956425	0.693983	-3.990116
C	-2.563835	2.025710	-2.925122
C	-2.484175	1.002773	-3.896745
H	-1.107724	-0.234379	-5.042086
H	-3.549880	2.415256	-2.627142
C	-3.731415	0.391838	-4.480088
H	-3.542134	-0.059047	-5.474044
H	-4.113288	-0.418503	-3.819323
H	-4.546785	1.135912	-4.578944

```
  Cl      1.383267      3.077148     -2.286457
  H      -1.500267      3.364053     -1.574889
TS1_PrOAc.xyz
81
Coordinates from ORCA-job TS1 E -2016.071498210046
  Pd      0.760167      1.229591     -0.812586
  N      -0.737615     -0.597387      1.155520
  C      -2.028379     -0.112634      0.749426
  N       1.396623     -0.878722      1.297590
  C      -2.813486     -0.920424     -0.113181
  C      -4.060621     -0.410731     -0.519703
  H      -4.693857     -1.004006     -1.194901
  C      -4.506329      0.844209     -0.079685
  H      -5.482640      1.226349     -0.415581
  C      -3.719712      1.608446      0.789865
  H      -4.086903      2.585724      1.139341
  C      -2.463918      1.144661      1.230970
  C      -2.302368     -2.268235     -0.613652
  H      -1.662694     -2.695764      0.186318
  C      -1.411926     -2.085695     -1.856214
  H      -0.581802     -1.376531     -1.662658
  H      -0.975121     -3.056797     -2.168639
  H      -2.001621     -1.681281     -2.703700
  C      -3.422554     -3.280872     -0.884430
  H      -4.048896     -2.981749     -1.750350
  H      -2.985892     -4.270677     -1.126352
  H      -4.088779     -3.402901     -0.006541
  C      -1.640361      1.974833      2.208110
  H      -0.715945      1.407898      2.438736
  C      -1.198929      3.308307      1.584291
  H      -2.068989      3.931034      1.289175
  H      -0.589938      3.890538      2.305895
  H      -0.576978      3.117337      0.682557
  C      -2.397330      2.186487      3.530609
  H      -2.688172      1.219418      3.988840
  H      -1.760860      2.733858      4.255788
  H      -3.320787      2.782742      3.378214
  C       0.448563     -0.153984      0.612835
  C      -0.529960     -1.555267      2.147001
  H      -1.358609     -2.012142      2.696368
  C       0.824618     -1.734733      2.236732
  H       1.429129     -2.382331      2.878705
  C       2.811056     -0.719713      1.097696
  C       3.443931     -1.473239      0.080564
  C       4.828731     -1.287656     -0.097826
  H       5.351280     -1.851151     -0.885905
  C       5.546406     -0.392427      0.707056
  H       6.628107     -0.260566      0.550233
  C       4.892678      0.337855      1.709305
  H       5.465661      1.043014      2.330456
```

```
  C       3.509627      0.189435      1.928943
  C       2.660586     -2.405783     -0.833548
  H       1.641293     -2.510949     -0.409843
  C       3.279419     -3.809970     -0.907762
  H       3.374561     -4.264331      0.099274
  H       2.645868     -4.476106     -1.528233
  H       4.288453     -3.789012     -1.368794
  C       2.506843     -1.773001     -2.228300
  H       3.494485     -1.632160     -2.715200
  H       1.885209     -2.415901     -2.885012
  H       2.012926     -0.779056     -2.144963
  C       2.792853      1.028817      2.979377
  H       1.774872      0.608984      3.109474
  C       2.622692      2.475531      2.480401
  H       2.077643      2.492313      1.512077
  H       2.047050      3.076520      3.214511
  H       3.608412      2.964064      2.331977
  C       3.488279      0.977366      4.348038
  H       4.498893      1.433862      4.312073
  H       2.898203      1.540846      5.099205
  H       3.598371     -0.065724      4.707992
  C      -0.158670      2.009985     -2.642641
  C      -0.033587      1.054042     -3.676438
  C      -1.422987      2.548339     -2.307733
  C      -1.198403      0.541537     -4.264075
  H       0.959265      0.703035     -3.990587
  C      -2.565369      2.008835     -2.911028
  C      -2.482631      0.989164     -3.885655
  H      -1.102435     -0.236423     -5.038410
  H      -3.552794      2.390934     -2.608263
  C      -3.728394      0.372875     -4.466788
  H      -3.536864     -0.086359     -5.456520
  H      -4.113481     -0.431360     -3.800335
  H      -4.543448      1.116092     -4.575619
  Cl      1.373752      3.077733     -2.281593
  H      -1.505469      3.351142     -1.562293
TS1_t-BuOH.xyz
81
Coordinates from ORCA-job TS1 E -2016.074184813662
  Pd      0.754762      1.234044     -0.817770
  N      -0.737274     -0.596890      1.153755
  C      -2.029177     -0.113925      0.748636
  N       1.397554     -0.872714      1.295014
  C      -2.812655     -0.920965     -0.116348
  C      -4.058976     -0.410260     -0.524922
  H      -4.690511     -1.002034     -1.203099
  C      -4.506064      0.843697     -0.083008
  H      -5.481482      1.226497     -0.420744
  C      -3.722349      1.605766      0.791382
  H      -4.091327      2.581518      1.143310
```

C	-2.466902	1.141583	1.233488
C	-2.303296	-2.270379	-0.614722
H	-1.661002	-2.695664	0.184207
C	-1.417688	-2.093894	-1.861681
H	-0.581615	-1.390715	-1.671948
H	-0.987935	-3.067834	-2.174856
H	-2.008931	-1.687210	-2.707193
C	-3.425522	-3.282951	-0.877859
H	-4.054975	-2.985941	-1.742189
H	-2.990179	-4.273847	-1.117627
H	-4.087898	-3.401058	0.003440
C	-1.645993	1.969353	2.214985
H	-0.719593	1.404527	2.443027
C	-1.210751	3.308721	1.599085
H	-2.084018	3.930597	1.311932
H	-0.601063	3.887447	2.322971
H	-0.592180	3.127460	0.693067
C	-2.403848	2.170712	3.538646
H	-2.689546	1.199662	3.991622
H	-1.769581	2.717212	4.266393
H	-3.330020	2.763363	3.388706
C	0.447287	-0.151377	0.610526
C	-0.526757	-1.553382	2.145828
H	-1.354056	-2.012356	2.695665
C	0.828381	-1.729446	2.235018
H	1.434831	-2.375283	2.877077
C	2.811013	-0.710700	1.091356
C	3.442888	-1.465713	0.074489
C	4.825999	-1.273889	-0.111629
H	5.347780	-1.839294	-0.898966
C	5.542930	-0.371005	0.685730
H	6.623177	-0.234163	0.522996
C	4.890546	0.359197	1.689182
H	5.463219	1.068647	2.305870
C	3.509302	0.203995	1.916874
C	2.661527	-2.411422	-0.827868
H	1.639201	-2.505397	-0.409186
C	3.277217	-3.818599	-0.869643
H	3.360835	-4.253670	0.146860
H	2.647837	-4.494659	-1.483589
H	4.290838	-3.807344	-1.320833
C	2.520148	-1.808019	-2.236729
H	3.511222	-1.685643	-2.721634
H	1.897822	-2.461352	-2.882352
H	2.032775	-0.809477	-2.180196
C	2.796688	1.037158	2.975284
H	1.775033	0.624456	3.098603
C	2.640317	2.492539	2.497640
H	2.092138	2.529750	1.531872
H	2.072601	3.088190	3.242107

```
  H       3.630368      2.973656      2.354028
  C       3.490442      0.960494      4.343953
  H       4.506215      1.405798      4.313434
  H       2.905787      1.520743      5.101746
  H       3.587865     -0.088497      4.690056
  C      -0.158247      2.010783     -2.647313
  C      -0.034821      1.052815     -3.679305
  C      -1.419749      2.558365     -2.317467
  C      -1.201112      0.545788     -4.269297
  H       0.956679      0.694873     -3.990210
  C      -2.563836      2.024222     -2.923409
  C      -2.484033      1.001589     -3.895321
  H      -1.107397     -0.234502     -5.041534
  H      -3.549967      2.413076     -2.624841
  C      -3.731226      0.390160     -4.478283
  H      -3.541902     -0.061442     -5.471911
  H      -4.113296     -0.419658     -3.816976
  H      -4.546596      1.134150     -4.577913
  Cl      1.382529      3.077038     -2.286013
  H      -1.500404      3.362926     -1.573476
TS1_n-BuOAc.xyz
81
Coordinates from ORCA-job TS1 E -2016.071090852364
  Pd      0.761083      1.229127     -0.811660
  N      -0.737661     -0.597261      1.155820
  C      -2.028241     -0.112299      0.749457
  N       1.396485     -0.879476      1.298071
  C      -2.813483     -0.920267     -0.112830
  C      -4.060748     -0.410814     -0.519128
  H      -4.694168     -1.004373     -1.193891
  C      -4.506354      0.844255     -0.079454
  H      -5.482802      1.226227     -0.415142
  C      -3.719406      1.608883      0.789402
  H      -4.086421      2.586367      1.138477
  C      -2.463564      1.145242      1.230455
  C      -2.302019     -2.267849     -0.613506
  H      -1.662752     -2.695630      0.186673
  C      -1.410819     -2.084483     -1.855391
  H      -0.581608     -1.374397     -1.661311
  H      -0.972929     -3.055175     -2.167597
  H      -2.000263     -1.680506     -2.703233
  C      -3.421843     -3.280565     -0.885383
  H      -4.047691     -2.981250     -1.751604
  H      -2.984930     -4.270213     -1.127502
  H      -4.088682     -3.403120     -0.008029
  C      -1.639750      1.975818      2.207017
  H      -0.715639      1.408605      2.438141
  C      -1.197368      3.308417      1.582059
  H      -2.066930      3.931222      1.285611
  H      -0.588620      3.891220      2.303397
```

H	-0.574774	3.116031	0.681061
C	-2.396735	2.189004	3.529252
H	-2.688393	1.222532	3.988226
H	-1.760021	2.736502	4.254121
H	-3.319769	2.785792	3.376388
C	0.448782	-0.154190	0.613268
C	-0.530483	-1.555375	2.147193
H	-1.359358	-2.011930	2.696457
C	0.824004	-1.735389	2.237040
H	1.428185	-2.383292	2.878996
C	2.811067	-0.721027	1.098648
C	3.443935	-1.474281	0.081340
C	4.828959	-1.289636	-0.096088
H	5.351511	-1.852826	-0.884365
C	5.546864	-0.395612	0.709859
H	6.628755	-0.264494	0.553761
C	4.893104	0.334589	1.712106
H	5.466242	1.039023	2.333909
C	3.509829	0.187172	1.930758
C	2.660220	-2.404933	-0.834365
H	1.641341	-2.511550	-0.409978
C	3.279325	-3.808727	-0.912980
H	3.376079	-4.265684	0.092731
H	2.645097	-4.473468	-1.534254
H	4.287692	-3.786559	-1.375419
C	2.504845	-1.768194	-2.227156
H	3.492023	-1.624923	-2.714283
H	1.883250	-2.409620	-2.885350
H	2.010122	-0.774890	-2.140189
C	2.792619	1.027325	2.980252
H	1.775386	0.606105	3.111844
C	2.619731	2.472628	2.478102
H	2.074692	2.486159	1.509682
H	2.043082	3.074268	3.210900
H	3.604583	2.962536	2.328436
C	3.488849	0.979947	4.348600
H	4.498486	1.438503	4.311414
H	2.898038	1.543688	5.098991
H	3.601378	-0.062177	4.710604
C	-0.158785	2.009929	-2.641864
C	-0.033421	1.054252	-3.675882
C	-1.423513	2.546925	-2.306254
C	-1.197998	0.540904	-4.263177
H	0.959635	0.704276	-3.990488
C	-2.565621	2.006592	-2.909158
C	-2.482423	0.987323	-3.884143
H	-1.101673	-0.236730	-5.037800
H	-3.553241	2.387702	-2.605817
C	-3.727956	0.370319	-4.465036
H	-3.536091	-0.090004	-5.454202

```
  H      -4.113495     -0.433117     -3.797865
  H      -4.542966      1.113411     -4.575212
  Cl      1.372400      3.077951     -2.280858
  H      -1.506274      3.349539     -1.560679
TS1_MIBK.xyz
81
Coordinates from ORCA-job TS1 E -2016.073830581824
  Pd      0.755429      1.233559     -0.816986
  N      -0.737270     -0.597241      1.153837
  C      -2.029073     -0.114141      0.748642
  N       1.397503     -0.873523      1.295255
  C      -2.812673     -0.921276     -0.116115
  C      -4.059179     -0.410824     -0.524332
  H      -4.690873     -1.002798     -1.202176
  C      -4.506230      0.843161     -0.082518
  H      -5.481811      1.225800     -0.419966
  C      -3.722216      1.605514      0.791314
  H      -4.091072      2.581384      1.143029
  C      -2.466660      1.141487      1.233219
  C      -2.302885     -2.270337     -0.614946
  H      -1.660874     -2.695920      0.184070
  C      -1.416671     -2.092748     -1.861307
  H      -0.581551     -1.388586     -1.671021
  H      -0.985752     -3.066190     -2.174461
  H      -2.007819     -1.686446     -2.707046
  C      -3.424684     -3.283060     -0.879221
  H      -4.053786     -2.985759     -1.743714
  H      -2.989012     -4.273712     -1.119409
  H      -4.087542     -3.401911      0.001620
  C      -1.645489      1.969550      2.214237
  H      -0.719259      1.404548      2.442507
  C      -1.209645      3.308300      1.597479
  H      -2.082593      3.930199      1.309386
  H      -0.600128      3.887471      2.321146
  H      -0.590608      3.125982      0.691990
  C      -2.403224      2.171955      3.537800
  H      -2.689437      1.201316      3.991339
  H      -1.768734      2.718566      4.265271
  H      -3.329138      2.764957      3.387645
  C       0.447462     -0.151812      0.610747
  C      -0.527040     -1.553951      2.145783
  H      -1.354479     -2.012788      2.695499
  C       0.828043     -1.730292      2.235077
  H       1.434307     -2.376321      2.877100
  C       2.811076     -0.711734      1.092148
  C       3.443223     -1.466581      0.075357
  C       4.826565     -1.275506     -0.109631
  H       5.348551     -1.840676     -0.896987
  C       5.543463     -0.373505      0.688705
  H       6.623910     -0.237257      0.526837
```

```
  C       4.890750      0.356781      1.691847
  H       5.463350      1.065791      2.309092
  C       3.509253      0.202393      1.918365
  C       2.661702     -2.410577     -0.828630
  H       1.639747     -2.506067     -0.409348
  C       3.277880     -3.817366     -0.874720
  H       3.362917     -4.255051      0.140546
  H       2.648047     -4.492108     -1.489651
  H       4.290954     -3.804772     -1.327101
  C       2.518747     -1.803188     -2.235635
  H       3.509399     -1.678269     -2.720757
  H       1.896601     -2.455148     -2.882825
  H       2.030445     -0.805247     -2.175512
  C       2.795929      1.036463      2.975547
  H       1.774661      0.622968      3.099473
  C       2.638064      2.490751      2.495049
  H       2.090607      2.525319      1.528748
  H       2.069127      3.087172      3.237977
  H       3.627650      2.972744      2.351146
  C       3.489570      0.963007      4.344404
  H       4.504767      1.409586      4.313387
  H       2.904142      1.523822      5.101182
  H       3.588378     -0.085240      4.692382
  C      -0.158398      2.011090     -2.646431
  C      -0.034747      1.053525     -3.678789
  C      -1.420301      2.557332     -2.315789
  C      -1.200847      0.545758     -4.268457
  H       0.956943      0.696599     -3.990213
  C      -2.564164      2.022464     -2.921364
  C      -2.483955      1.000331     -3.893753
  H      -1.106824     -0.234132     -5.041063
  H      -3.550481      2.410306     -2.622121
  C      -3.730958      0.388211     -4.476431
  H      -3.541293     -0.064652     -5.469423
  H      -4.113542     -0.420677     -3.814269
  H      -4.546232      1.132135     -4.577522
  Cl      1.381196      3.077538     -2.285051
  H      -1.501211      3.361553     -1.571492
TS1_2-pentanol.xyz
81
Coordinates from ORCA-job TS1 E -2016.073857627403
  Pd      0.755245      1.233480     -0.817220
  N      -0.737246     -0.597320      1.153809
  C      -2.029084     -0.114272      0.748658
  N       1.397548     -0.873467      1.295135
  C      -2.812659     -0.921411     -0.116117
  C      -4.059138     -0.410941     -0.524400
  H      -4.690796     -1.002904     -1.202290
  C      -4.506206      0.843040     -0.082591
  H      -5.481770      1.225686     -0.420077
```

C	-3.722235	1.605378	0.791300
H	-4.091110	2.581241	1.143017
C	-2.466692	1.141349	1.233245
C	-2.302890	-2.270492	-0.614912
H	-1.660623	-2.695925	0.183974
C	-1.417025	-2.092988	-1.861531
H	-0.581836	-1.388841	-1.671487
H	-0.986205	-3.066453	-2.174744
H	-2.008388	-1.686721	-2.707139
C	-3.424695	-3.283331	-0.878734
H	-4.054101	-2.986172	-1.743054
H	-2.989017	-4.273974	-1.118950
H	-4.087256	-3.402136	0.002337
C	-1.645533	1.969419	2.214269
H	-0.719394	1.404331	2.442702
C	-1.209485	3.308064	1.597416
H	-2.082350	3.930029	1.309215
H	-0.599941	3.887228	2.321068
H	-0.590425	3.125593	0.691969
C	-2.403371	2.172057	3.537737
H	-2.689757	1.201501	3.991344
H	-1.768870	2.718651	4.265211
H	-3.329185	2.765176	3.387434
C	0.447429	-0.151847	0.610649
C	-0.526912	-1.553979	2.145779
H	-1.354298	-2.012859	2.695540
C	0.828189	-1.730229	2.235020
H	1.434523	-2.376188	2.877048
C	2.811096	-0.711454	1.092043
C	3.443398	-1.466223	0.075286
C	4.826698	-1.274863	-0.109719
H	5.348803	-1.839971	-0.897041
C	5.543413	-0.372663	0.688567
H	6.623830	-0.236196	0.526684
C	4.890551	0.357529	1.691680
H	5.462998	1.066690	2.308900
C	3.509084	0.202858	1.918210
C	2.662066	-2.410447	-0.828627
H	1.640142	-2.506138	-0.409316
C	3.278560	-3.817102	-0.874629
H	3.363713	-4.254700	0.140664
H	2.648870	-4.492025	-1.489507
H	4.291623	-3.804294	-1.327032
C	2.518968	-1.803217	-2.235687
H	3.509585	-1.678224	-2.720862
H	1.896889	-2.455334	-2.882784
H	2.030532	-0.805336	-2.175675
C	2.795691	1.036700	2.975527
H	1.774122	0.623730	3.098693
C	2.638916	2.491415	2.495990

```
  H       2.091826      2.527045      1.529533
  H       2.070065      3.087649      3.239132
  H       3.628834      2.972937      2.352805
  C       3.488667      0.961854      4.344659
  H       4.504246      1.407614      4.314332
  H       2.903357      1.522662      5.101535
  H       3.586469     -0.086694      4.692010
  C      -0.158541      2.010850     -2.646627
  C      -0.034954      1.053227     -3.678940
  C      -1.420385      2.557256     -2.316047
  C      -1.201091      0.545582     -4.268641
  H       0.956703      0.696165     -3.990313
  C      -2.564291      2.022508     -2.921665
  C      -2.484163      1.000335     -3.894016
  H      -1.107130     -0.234352     -5.041211
  H      -3.550575      2.410486     -2.622489
  C      -3.731203      0.388337     -4.476742
  H      -3.541711     -0.063903     -5.470052
  H      -4.113450     -0.421031     -3.814972
  H      -4.546634      1.132174     -4.577164
  Cl      1.381216      3.077295     -2.285359
  H      -1.501244      3.361496     -1.571764
TS1_Heptane.xyz
81
Coordinates from ORCA-job TS1 E -2016.065025176847
  Pd      0.768279      1.245315     -0.783817
  N      -0.737213     -0.577917      1.171811
  C      -2.026524     -0.104833      0.747331
  N       1.396936     -0.858703      1.331924
  C      -2.792568     -0.921326     -0.123605
  C      -4.042353     -0.428260     -0.540847
  H      -4.662242     -1.030637     -1.220235
  C      -4.507815      0.820773     -0.106187
  H      -5.486433      1.189655     -0.450064
  C      -3.737458      1.596240      0.767185
  H      -4.118710      2.570010      1.111121
  C      -2.481642      1.147504      1.222088
  C      -2.256311     -2.258783     -0.624733
  H      -1.615133     -2.679042      0.178306
  C      -1.359547     -2.053875     -1.858927
  H      -0.549155     -1.324340     -1.657659
  H      -0.899114     -3.014460     -2.170299
  H      -1.950577     -1.660017     -2.710020
  C      -3.357140     -3.288860     -0.908392
  H      -3.980276     -2.997464     -1.779331
  H      -2.903409     -4.270805     -1.151019
  H      -4.030349     -3.425651     -0.038005
  C      -1.676402      1.988198      2.204944
  H      -0.765990      1.414341      2.470099
  C      -1.200951      3.301935      1.564831
```

H	-2.054754	3.925820	1.227097
H	-0.608605	3.896080	2.290450
H	-0.552290	3.080874	0.688764
C	-2.462592	2.235293	3.503705
H	-2.782503	1.282045	3.971397
H	-1.835459	2.784464	4.235566
H	-3.371803	2.844800	3.321244
C	0.453465	-0.137486	0.633992
C	-0.537252	-1.530422	2.170219
H	-1.370039	-1.983230	2.716232
C	0.816272	-1.710120	2.270548
H	1.415163	-2.357237	2.917971
C	2.813873	-0.731849	1.122367
C	3.415410	-1.478993	0.082605
C	4.804283	-1.334172	-0.100879
H	5.302355	-1.894265	-0.906882
C	5.556146	-0.486257	0.721693
H	6.640651	-0.386415	0.561291
C	4.933426	0.238307	1.747396
H	5.534068	0.906214	2.382267
C	3.548275	0.131803	1.972175
C	2.600600	-2.367597	-0.847629
H	1.576694	-2.441736	-0.428330
C	3.167478	-3.792478	-0.941762
H	3.245082	-4.265776	0.058037
H	2.511436	-4.426065	-1.573000
H	4.177235	-3.803014	-1.401524
C	2.472450	-1.706646	-2.231285
H	3.465643	-1.585495	-2.711914
H	1.834415	-2.318189	-2.901902
H	2.006841	-0.700665	-2.128220
C	2.854701	0.977576	3.033246
H	1.888488	0.485989	3.270329
C	2.529789	2.370795	2.460912
H	1.935559	2.282982	1.524988
H	1.948450	2.970156	3.192049
H	3.462628	2.923510	2.223143
C	3.648508	1.076029	4.343476
H	4.596559	1.636999	4.211161
H	3.055571	1.617024	5.108501
H	3.899810	0.074129	4.747008
C	-0.168625	2.029252	-2.616167
C	-0.026177	1.087810	-3.660923
C	-1.444897	2.532702	-2.271937
C	-1.179684	0.558124	-4.254198
H	0.973994	0.764261	-3.980513
C	-2.575213	1.976595	-2.880311
C	-2.472214	0.973087	-3.869204
H	-1.067783	-0.206519	-5.039639
H	-3.570035	2.332563	-2.570723

```
  C      -3.706007      0.340733     -4.458693
  H      -3.501779     -0.119258     -5.445604
  H      -4.090793     -0.464847     -3.793485
  H      -4.527962      1.074684     -4.579922
  Cl      1.335665      3.114101     -2.244050
  H      -1.542424      3.324930     -1.517626
TS1_Cyclohexane.xyz
81
Coordinates from ORCA-job TS1 E -2016.065488244023
  Pd      0.767703      1.245473     -0.784601
  N      -0.737079     -0.578194      1.171810
  C      -2.026574     -0.105524      0.747365
  N       1.397140     -0.858126      1.331805
  C      -2.792245     -0.922031     -0.123927
  C      -4.041822     -0.428849     -0.541808
  H      -4.661302     -1.031069     -1.221722
  C      -4.507541      0.820132     -0.107176
  H      -5.485904      1.189128     -0.451655
  C      -3.737800      1.595356      0.767007
  H      -4.119339      2.568973      1.111064
  C      -2.482133      1.146595      1.222374
  C      -2.256161     -2.259763     -0.624562
  H      -1.614738     -2.679642      0.178458
  C      -1.359884     -2.055800     -1.859272
  H      -0.548551     -1.327206     -1.658412
  H      -0.900493     -3.016850     -2.170728
  H      -1.950998     -1.661502     -2.710139
  C      -3.357270     -3.289839     -0.907258
  H      -3.980685     -2.998738     -1.778083
  H      -2.903700     -4.271967     -1.149449
  H      -4.030080     -3.425987     -0.036463
  C      -1.677490      1.987065      2.205917
  H      -0.766939      1.413440      2.471101
  C      -1.202582      3.301476      1.566741
  H      -2.056686      3.925222      1.229537
  H      -0.610431      3.895262      2.292823
  H      -0.553981      3.081490      0.690359
  C      -2.464243      2.233126      3.504547
  H      -2.783706      1.279438      3.971651
  H      -1.837591      2.782276      4.236835
  H      -3.373691      2.842228      3.321925
  C       0.453358     -0.137416      0.633917
  C      -0.536703     -1.530511      2.170306
  H      -1.369300     -1.983641      2.716375
  C       0.816903     -1.709683      2.270562
  H       1.416101     -2.356501      2.918025
  C       2.813972     -0.730933      1.121833
  C       3.415383     -1.478248      0.082089
  C       4.804131     -1.332853     -0.102150
  H       5.302086     -1.893135     -0.908109
```

```
  C       5.555985     -0.484179      0.719719
  H       6.640366     -0.383848      0.558735
  C       4.933418      0.240402      1.745557
  H       5.534064      0.908774      2.379956
  C       3.548406      0.133227      1.971118
  C       2.600700     -2.368116     -0.847057
  H       1.576470     -2.440975     -0.428363
  C       3.167098     -3.793433     -0.937860
  H       3.243175     -4.264823      0.062959
  H       2.511526     -4.427898     -1.568707
  H       4.177450     -3.805078     -1.396289
  C       2.473961     -1.710057     -2.232205
  H       3.467535     -1.590807     -2.712529
  H       1.835922     -2.322639     -2.901872
  H       2.009044     -0.703534     -2.131847
  C       2.855148      0.978572      3.032740
  H       1.888686      0.487333      3.269430
  C       2.530975      2.372426      2.461537
  H       1.936514      2.285578      1.525691
  H       1.949972      2.971469      3.193193
  H       3.464092      2.924864      2.224165
  C       3.649057      1.075612      4.343029
  H       4.597617      1.635774      4.210954
  H       3.056515      1.616652      5.108331
  H       3.899371      0.073210      4.745932
  C      -0.167737      2.029083     -2.616828
  C      -0.026341      1.086951     -3.661112
  C      -1.443241      2.535034     -2.273451
  C      -1.180540      0.558736     -4.254438
  H       0.973386      0.761508     -3.980212
  C      -2.574313      1.980382     -2.881935
  C      -2.472577      0.975973     -3.870070
  H      -1.069611     -0.206620     -5.039320
  H      -3.568682      2.338193     -2.572973
  C      -3.707107      0.344916     -4.459404
  H      -3.503687     -0.114331     -5.446826
  H      -4.091743     -0.461083     -3.794633
  H      -4.528721      1.079450     -4.579267
  Cl      1.338563      3.112844     -2.245226
  H      -1.539850      3.327783     -1.519532
TS1_i-PrOH.xyz
81
Coordinates from ORCA-job TS1 E -2016.074522673340
  Pd      0.753960      1.234747     -0.818417
  N      -0.737219     -0.596805      1.153541
  C      -2.029304     -0.114086      0.748622
  N       1.397706     -0.871925      1.294494
  C      -2.812581     -0.921015     -0.116672
  C      -4.058800     -0.410172     -0.525487
  H      -4.690124     -1.001745     -1.204045
```

C	-4.506058	0.843653	-0.083311
H	-5.481363	1.226545	-0.421271
C	-3.722713	1.605422	0.791712
H	-4.091923	2.580968	1.143979
C	-2.467318	1.141172	1.233946
C	-2.303468	-2.270634	-0.614797
H	-1.660760	-2.695609	0.183946
C	-1.418603	-2.094942	-1.862404
H	-0.581765	-1.392516	-1.673265
H	-0.989767	-3.069245	-2.175684
H	-2.010127	-1.688005	-2.707621
C	-3.425966	-3.283217	-0.876823
H	-4.055896	-2.986518	-1.740904
H	-2.990802	-4.274255	-1.116318
H	-4.087768	-3.400799	0.004973
C	-1.646756	1.968602	2.216033
H	-0.720261	1.403880	2.443980
C	-1.211945	3.308554	1.601042
H	-2.085464	3.930452	1.314726
H	-0.602133	3.886800	2.325217
H	-0.593737	3.128290	0.694572
C	-2.404882	2.168979	3.539695
H	-2.690224	1.197549	3.992072
H	-1.770878	2.715304	4.267800
H	-3.331233	2.761376	3.389865
C	0.447088	-0.150972	0.610187
C	-0.526236	-1.553189	2.145599
H	-1.353309	-2.012488	2.695529
C	0.828985	-1.728805	2.234601
H	1.435740	-2.374442	2.876591
C	2.811013	-0.709307	1.090322
C	3.442886	-1.464427	0.073499
C	4.825711	-1.271464	-0.113710
H	5.347498	-1.837025	-0.900944
C	5.542369	-0.367362	0.682573
H	6.622379	-0.229625	0.518994
C	4.890042	0.362728	1.686169
H	5.462520	1.072920	2.302212
C	3.509088	0.206327	1.914987
C	2.661948	-2.412050	-0.827236
H	1.639399	-2.505108	-0.408923
C	3.277794	-3.819299	-0.865201
H	3.360509	-4.251941	0.152407
H	2.648989	-4.496831	-1.478111
H	4.291816	-3.808837	-1.315511
C	2.521492	-1.812329	-2.237752
H	3.512816	-1.691813	-2.722607
H	1.899189	-2.467181	-2.881846
H	2.034538	-0.813463	-2.184407
C	2.797008	1.038444	2.974606

```
  H       1.774441      0.627482      3.096044
  C       2.644025      2.495482      2.500990
  H       2.096079      2.536755      1.535293
  H       2.077372      3.090210      3.246989
  H       3.635108      2.974957      2.359058
  C       3.489610      0.956578      4.343617
  H       4.506624      1.399154      4.314726
  H       2.905904      1.516498      5.102387
  H       3.583873     -0.093556      4.687106
  C      -0.158550      2.010739     -2.647882
  C      -0.035107      1.052613     -3.679698
  C      -1.419792      2.559254     -2.318701
  C      -1.201461      0.546140     -4.270111
  H       0.956295      0.693980     -3.990162
  C      -2.563964      2.025660     -2.925105
  C      -2.484306      1.002750     -3.896756
  H      -1.107857     -0.234367     -5.042138
  H      -3.550009      2.415189     -2.627104
  C      -3.731546      0.391815     -4.480098
  H      -3.542298     -0.058946     -5.474117
  H      -4.113332     -0.418626     -3.819405
  H      -4.546962      1.135855     -4.578819
  Cl      1.383152      3.077155     -2.286499
  H      -1.500397      3.363963     -1.574834
TS1_n-BuOH.xyz
81
Coordinates from ORCA-job TS1 E -2016.074305435780
  Pd      0.754493      1.234297     -0.817922
  N      -0.737264     -0.596864      1.153713
  C      -2.029220     -0.113979      0.748635
  N       1.397596     -0.872431      1.294930
  C      -2.812606     -0.920960     -0.116499
  C      -4.058886     -0.410202     -0.525167
  H      -4.690332     -1.001889     -1.203504
  C      -4.506052      0.843693     -0.083135
  H      -5.481428      1.226526     -0.420957
  C      -3.722490      1.605637      0.791513
  H      -4.091563      2.581303      1.143582
  C      -2.467065      1.141426      1.233673
  C      -2.303323     -2.270437     -0.614802
  H      -1.661008     -2.695687      0.184124
  C      -1.417809     -2.094198     -1.861866
  H      -0.581455     -1.391338     -1.672197
  H      -0.988388     -3.068268     -2.175084
  H      -2.009024     -1.687341     -2.707322
  C      -3.425653     -3.282947     -0.877769
  H      -4.055121     -2.985962     -1.742092
  H      -2.990382     -4.273900     -1.117431
  H      -4.087968     -3.400887      0.003597
  C      -1.646306      1.969060      2.215414
```

H	-0.719835	1.404313	2.443368
C	-1.211311	3.308700	1.599911
H	-2.084709	3.930541	1.313090
H	-0.601621	3.887257	2.323932
H	-0.592856	3.127889	0.693724
C	-2.404247	2.169939	3.539102
H	-2.689746	1.198706	3.991809
H	-1.770099	2.716375	4.267000
H	-3.330526	2.762446	3.389251
C	0.447223	-0.151235	0.610477
C	-0.526610	-1.553321	2.145784
H	-1.353845	-2.012411	2.695631
C	0.828554	-1.729226	2.234946
H	1.435092	-2.375000	2.876990
C	2.811007	-0.710285	1.091063
C	3.442803	-1.465357	0.074181
C	4.825821	-1.273218	-0.112362
H	5.347543	-1.838700	-0.899688
C	5.542738	-0.369970	0.684614
H	6.622905	-0.232876	0.521552
C	4.890453	0.360207	1.688161
H	5.463130	1.069858	2.304621
C	3.509310	0.204666	1.916294
C	2.661527	-2.411719	-0.827573
H	1.639074	-2.505180	-0.409099
C	3.277081	-3.819014	-0.867781
H	3.360196	-4.253126	0.149171
H	2.647881	-4.495574	-1.481361
H	4.290901	-3.808228	-1.318538
C	2.520671	-1.809758	-2.237097
H	3.511887	-1.688197	-2.721915
H	1.898341	-2.463633	-2.882164
H	2.033546	-0.811046	-2.181843
C	2.796926	1.037524	2.975109
H	1.775165	0.625044	3.098268
C	2.640972	2.493247	2.498375
H	2.092559	2.531272	1.532778
H	2.073619	3.088625	3.243331
H	3.631151	2.974127	2.354855
C	3.490761	0.959869	4.343695
H	4.506705	1.404789	4.313291
H	2.906364	1.519934	5.101824
H	3.587776	-0.089352	4.689211
C	-0.158268	2.010838	-2.647458
C	-0.034836	1.052827	-3.679401
C	-1.419673	2.558772	-2.317865
C	-1.201149	0.546020	-4.269564
H	0.956628	0.694627	-3.990139
C	-2.563791	2.024847	-2.923993
C	-2.484042	1.002123	-3.895820

```
  H      -1.107473     -0.234338     -5.041735
  H      -3.549890      2.413957     -2.625640
  C      -3.731250      0.390899     -4.478949
  H      -3.541950     -0.060364     -5.472734
  H      -4.113230     -0.419167     -3.817899
  H      -4.546632      1.134915     -4.578231
  Cl      1.382843      3.077102     -2.286076
  H      -1.500306      3.363389     -1.573921
TS1_Toluene.xyz
81
Coordinates from ORCA-job TS1 E -2016.066786886503
  Pd      0.766610      1.234389     -0.797385
  N      -0.738141     -0.589255      1.161683
  C      -2.027270     -0.105938      0.749213
  N       1.395357     -0.878125      1.307726
  C      -2.808978     -0.917849     -0.112324
  C      -4.057885     -0.414294     -0.519664
  H      -4.689597     -1.012438     -1.191842
  C      -4.507541      0.840477     -0.084210
  H      -5.485678      1.217763     -0.420268
  C      -3.722093      1.611082      0.780153
  H      -4.091380      2.589239      1.124797
  C      -2.465790      1.152363      1.224232
  C      -2.289385     -2.262098     -0.613191
  H      -1.655023     -2.690479      0.190798
  C      -1.388085     -2.069663     -1.846269
  H      -0.568853     -1.350032     -1.645096
  H      -0.938202     -3.035993     -2.155181
  H      -1.973250     -1.670493     -2.699017
  C      -3.402551     -3.278355     -0.898055
  H      -4.021767     -2.979260     -1.769199
  H      -2.960279     -4.265623     -1.140185
  H      -4.077554     -3.406990     -0.027811
  C      -1.644787      1.988605      2.198107
  H      -0.725597      1.418352      2.440518
  C      -1.188867      3.310960      1.561697
  H      -2.051660      3.934241      1.246706
  H      -0.585586      3.900558      2.282041
  H      -0.555887      3.102357      0.671425
  C      -2.408711      2.219948      3.513147
  H      -2.712045      1.260606      3.979441
  H      -1.772771      2.769115      4.237426
  H      -3.325979      2.822890      3.350140
  C       0.451044     -0.148394      0.621332
  C      -0.535673     -1.549423      2.152155
  H      -1.366819     -2.003516      2.699729
  C       0.817952     -1.733741      2.244207
  H       1.418779     -2.384676      2.886023
  C       2.811393     -0.730873      1.108550
  C       3.436223     -1.479933      0.083590
```

C	4.823511	-1.310595	-0.089574
H	5.339951	-1.870486	-0.884062
C	5.551361	-0.435789	0.727564
H	6.634999	-0.316577	0.574507
C	4.905314	0.291572	1.736506
H	5.486318	0.982369	2.365878
C	3.520289	0.159731	1.951090
C	2.642340	-2.387923	-0.845916
H	1.624267	-2.494793	-0.419269
C	3.249515	-3.794713	-0.955070
H	3.351213	-4.271714	0.040819
H	2.605505	-4.443010	-1.583654
H	4.254265	-3.773418	-1.425378
C	2.484903	-1.720993	-2.224245
H	3.471194	-1.568761	-2.710338
H	1.859520	-2.345689	-2.894786
H	1.992842	-0.728504	-2.111612
C	2.806862	1.004741	2.999088
H	1.802640	0.562339	3.160094
C	2.589664	2.432889	2.465309
H	2.033305	2.408798	1.503140
H	2.007930	3.038152	3.191215
H	3.560114	2.942497	2.289636
C	3.527592	1.006314	4.354938
H	4.521817	1.495059	4.293658
H	2.932834	1.567444	5.104105
H	3.679024	-0.023809	4.736640
C	-0.165433	2.016636	-2.630271
C	-0.030729	1.065988	-3.667761
C	-1.437439	2.532151	-2.288026
C	-1.189185	0.538787	-4.254018
H	0.966556	0.732608	-3.986468
C	-2.572880	1.978395	-2.889564
C	-2.478320	0.965502	-3.869863
H	-1.084044	-0.233577	-5.032787
H	-3.564771	2.343648	-2.581164
C	-3.717346	0.335810	-4.451190
H	-3.518220	-0.132836	-5.435037
H	-4.104296	-0.462628	-3.778708
H	-4.535520	1.073499	-4.574962
Cl	1.347316	3.094414	-2.265881
H	-1.528451	3.330969	-1.539720

TS1_2-methyl-1-butanol.xyz
81
Coordinates from ORCA-job TS1 E -2016.074057863492

Pd	0.755082	1.233775	-0.817625
N	-0.737259	-0.596949	1.153766
C	-2.029120	-0.113959	0.748572
N	1.397540	-0.872968	1.295115
C	-2.812629	-0.921088	-0.116292

C	-4.059002	-0.410489	-0.524802
H	-4.690587	-1.002376	-1.202829
C	-4.506074	0.843513	-0.083021
H	-5.481539	1.226242	-0.420704
C	-3.722270	1.605734	0.791142
H	-4.091204	2.581556	1.142917
C	-2.466804	1.141620	1.233247
C	-2.303127	-2.270428	-0.614706
H	-1.660956	-2.695760	0.184303
C	-1.417271	-2.093680	-1.861448
H	-0.581513	-1.390171	-1.671553
H	-0.987134	-3.067485	-2.174529
H	-2.008441	-1.687169	-2.707086
C	-3.425215	-3.283056	-0.878181
H	-4.054516	-2.986007	-1.742611
H	-2.989768	-4.273896	-1.118000
H	-4.087784	-3.401336	0.002951
C	-1.645804	1.969558	2.214523
H	-0.719610	1.404538	2.442919
C	-1.209995	3.308477	1.598065
H	-2.082994	3.930444	1.310286
H	-0.600368	3.887424	2.321824
H	-0.591152	3.126480	0.692375
C	-2.403767	2.171747	3.537991
H	-2.689928	1.201009	3.991346
H	-1.769403	2.718310	4.265607
H	-3.329693	2.764698	3.387717
C	0.447376	-0.151519	0.610568
C	-0.526872	-1.553466	2.145851
H	-1.354235	-2.012343	2.695664
C	0.828241	-1.729650	2.235098
H	1.434609	-2.375530	2.877185
C	2.811047	-0.711094	1.091669
C	3.443002	-1.466083	0.074845
C	4.826213	-1.274613	-0.110825
H	5.348057	-1.839969	-0.898150
C	5.543167	-0.372099	0.686917
H	6.623500	-0.235542	0.524531
C	4.890681	0.358165	1.690245
H	5.463356	1.067413	2.307158
C	3.509328	0.203346	1.917466
C	2.661552	-2.411140	-0.828111
H	1.639337	-2.505584	-0.409246
C	3.277334	-3.818218	-0.871395
H	3.361401	-4.254229	0.144669
H	2.647776	-4.493773	-1.485716
H	4.290774	-3.806535	-1.322980
C	2.519707	-1.806329	-2.236326
H	3.510657	-1.683174	-2.721287
H	1.897389	-2.459119	-2.882507

```
  H       2.032107     -0.807945     -2.178548
  C       2.796481      1.036860      2.975429
  H       1.774908      0.623984      3.098870
  C       2.639772      2.491897      2.496846
  H       2.091888      2.528306      1.530873
  H       2.071675      3.087851      3.240783
  H       3.629721      2.973214      2.353187
  C       3.490087      0.961177      4.344211
  H       4.505715      1.406809      4.313620
  H       2.905170      1.521654      5.101634
  H       3.587848     -0.087583      4.690925
  C      -0.158214      2.010708     -2.647192
  C      -0.035039      1.052692     -3.679177
  C      -1.419695      2.558160     -2.317019
  C      -1.201440      0.545585     -4.268855
  H       0.956413      0.694829     -3.990311
  C      -2.563891      2.023929     -2.922627
  C      -2.484284      1.001304     -3.894556
  H      -1.107876     -0.234700     -5.041116
  H      -3.549967      2.412703     -2.623783
  C      -3.731606      0.389824     -4.477200
  H      -3.542446     -0.062109     -5.470711
  H      -4.113771     -0.419723     -3.815611
  H      -4.546896      1.133880     -4.577049
  Cl      1.382421      3.076724     -2.286178
  H      -1.500172      3.362745     -1.573049
start.xyz
81
Coordinates from ORCA-job TS1 E -2016.065025176847
  Pd      0.768279      1.245315     -0.783817
  N      -0.737213     -0.577917      1.171811
  C      -2.026524     -0.104833      0.747331
  N       1.396936     -0.858703      1.331924
  C      -2.792568     -0.921326     -0.123605
  C      -4.042353     -0.428260     -0.540847
  H      -4.662242     -1.030637     -1.220235
  C      -4.507815      0.820773     -0.106187
  H      -5.486433      1.189655     -0.450064
  C      -3.737458      1.596240      0.767185
  H      -4.118710      2.570010      1.111121
  C      -2.481642      1.147504      1.222088
  C      -2.256311     -2.258783     -0.624733
  H      -1.615133     -2.679042      0.178306
  C      -1.359547     -2.053875     -1.858927
  H      -0.549155     -1.324340     -1.657659
  H      -0.899114     -3.014460     -2.170299
  H      -1.950577     -1.660017     -2.710020
  C      -3.357140     -3.288860     -0.908392
  H      -3.980276     -2.997464     -1.779331
  H      -2.903409     -4.270805     -1.151019
```

H	-4.030349	-3.425651	-0.038005
C	-1.676402	1.988198	2.204944
H	-0.765990	1.414341	2.470099
C	-1.200951	3.301935	1.564831
H	-2.054754	3.925820	1.227097
H	-0.608605	3.896080	2.290450
H	-0.552290	3.080874	0.688764
C	-2.462592	2.235293	3.503705
H	-2.782503	1.282045	3.971397
H	-1.835459	2.784464	4.235566
H	-3.371803	2.844800	3.321244
C	0.453465	-0.137486	0.633992
C	-0.537252	-1.530422	2.170219
H	-1.370039	-1.983230	2.716232
C	0.816272	-1.710120	2.270548
H	1.415163	-2.357237	2.917971
C	2.813873	-0.731849	1.122367
C	3.415410	-1.478993	0.082605
C	4.804283	-1.334172	-0.100879
H	5.302355	-1.894265	-0.906882
C	5.556146	-0.486257	0.721693
H	6.640651	-0.386415	0.561291
C	4.933426	0.238307	1.747396
H	5.534068	0.906214	2.382267
C	3.548275	0.131803	1.972175
C	2.600600	-2.367597	-0.847629
H	1.576694	-2.441736	-0.428330
C	3.167478	-3.792478	-0.941762
H	3.245082	-4.265776	0.058037
H	2.511436	-4.426065	-1.573000
H	4.177235	-3.803014	-1.401524
C	2.472450	-1.706646	-2.231285
H	3.465643	-1.585495	-2.711914
H	1.834415	-2.318189	-2.901902
H	2.006841	-0.700665	-2.128220
C	2.854701	0.977576	3.033246
H	1.888488	0.485989	3.270329
C	2.529789	2.370795	2.460912
H	1.935559	2.282982	1.524988
H	1.948450	2.970156	3.192049
H	3.462628	2.923510	2.223143
C	3.648508	1.076029	4.343476
H	4.596559	1.636999	4.211161
H	3.055571	1.617024	5.108501
H	3.899810	0.074129	4.747008
C	-0.168625	2.029252	-2.616167
C	-0.026177	1.087810	-3.660923
C	-1.444897	2.532702	-2.271937
C	-1.179684	0.558124	-4.254198
H	0.973994	0.764261	-3.980513

```
  C      -2.575213      1.976595     -2.880311
  C      -2.472214      0.973087     -3.869204
  H      -1.067783     -0.206519     -5.039639
  H      -3.570035      2.332563     -2.570723
  C      -3.706007      0.340733     -4.458693
  H      -3.501779     -0.119258     -5.445604
  H      -4.090793     -0.464847     -3.793485
  H      -4.527962      1.074684     -4.579922
  Cl      1.335665      3.114101     -2.244050
  H      -1.542424      3.324930     -1.517626
TS1_MEK.xyz
81
Coordinates from ORCA-job TS1 E -2016.074375307766
  Pd      0.754302      1.234496     -0.818107
  N      -0.737233     -0.596673      1.153766
  C      -2.029230     -0.113929      0.748647
  N       1.397639     -0.872133      1.294874
  C      -2.812477     -0.920993     -0.116532
  C      -4.058735     -0.410300     -0.525372
  H      -4.690058     -1.002026     -1.203796
  C      -4.506020      0.843581     -0.083407
  H      -5.481366      1.226366     -0.421368
  C      -3.722621      1.605574      0.791358
  H      -4.091809      2.581211      1.143390
  C      -2.467210      1.141448      1.233654
  C      -2.303128     -2.270498     -0.614696
  H      -1.660203     -2.695314      0.183959
  C      -1.418437     -2.094478     -1.862372
  H      -0.582002     -1.391527     -1.673385
  H      -0.989117     -3.068587     -2.175604
  H      -2.010229     -1.687914     -2.707573
  C      -3.425381     -3.283383     -0.876571
  H      -4.055554     -2.986864     -1.740540
  H      -2.990009     -4.274302     -1.116192
  H      -4.087028     -3.401196      0.005314
  C      -1.646590      1.969122      2.215481
  H      -0.720107      1.404424      2.443525
  C      -1.211644      3.308823      1.600062
  H      -2.085074      3.930663      1.313338
  H      -0.601936      3.887331      2.324110
  H      -0.593245      3.128137      0.693810
  C      -2.404664      2.169934      3.539103
  H      -2.690113      1.198671      3.991774
  H      -1.770614      2.716413      4.267053
  H      -3.330973      2.762370      3.389157
  C       0.447189     -0.151037      0.610439
  C      -0.526479     -1.553043      2.145894
  H      -1.353665     -2.012158      2.695796
  C       0.828701     -1.728882      2.234986
  H       1.435310     -2.374591      2.877033
```

```
  C       2.811016     -0.709917      1.090848
  C       3.442711     -1.464874      0.073811
  C       4.825670     -1.272544     -0.113000
  H       5.347312     -1.837959     -0.900430
  C       5.542628     -0.369233      0.683881
  H       6.622747     -0.231994      0.520614
  C       4.890455      0.360775      1.687628
  H       5.463171      1.070430      2.304053
  C       3.509372      0.205039      1.916031
  C       2.661431     -2.411484     -0.827685
  H       1.638920     -2.504686     -0.409303
  C       3.276886     -3.818851     -0.867183
  H       3.359766     -4.252533      0.149972
  H       2.647753     -4.495620     -1.480600
  H       4.290796     -3.808288     -1.317742
  C       2.520836     -1.810204     -2.237523
  H       3.512111     -1.689154     -2.722348
  H       1.898409     -2.464281     -2.882290
  H       2.033932     -0.811360     -2.182888
  C       2.797198      1.037535      2.975280
  H       1.775221      0.625469      3.098008
  C       2.642045      2.493709      2.499681
  H       2.093573      2.532853      1.534175
  H       2.075075      3.088818      3.245143
  H       3.632474      2.974167      2.356483
  C       3.490842      0.958467      4.343896
  H       4.507070      1.402767      4.313894
  H       2.906700      1.518324      5.102374
  H       3.587156     -0.091076      4.688628
  C      -0.158389      2.010777     -2.647611
  C      -0.034871      1.052741     -3.679516
  C      -1.419780      2.558828     -2.318185
  C      -1.201151      0.545991     -4.269806
  H       0.956602      0.694461     -3.990140
  C      -2.563866      2.024960     -2.924448
  C      -2.484063      1.002193     -3.896228
  H      -1.107434     -0.234408     -5.041929
  H      -3.549978      2.414143     -2.626230
  C      -3.731234      0.390992     -4.479455
  H      -3.541943     -0.059953     -5.473385
  H      -4.113017     -0.419334     -3.818611
  H      -4.546721      1.134931     -4.578414
  Cl      1.382860      3.077203     -2.286218
  H      -1.500480      3.363444     -1.574241
TS1_Benzylalcohol.xyz
81
Coordinates from ORCA-job TS1 E -2016.073767224277
  Pd      0.756457      1.232258     -0.817480
  N      -0.737041     -0.597501      1.153713
  C      -2.028663     -0.114608      0.747779
```

N	1.397646	-0.874018	1.295694
C	-2.811726	-0.922157	-0.117058
C	-4.058160	-0.412105	-0.525963
H	-4.689481	-1.004434	-1.203847
C	-4.505553	0.841993	-0.084824
H	-5.481022	1.224396	-0.422859
C	-3.722023	1.604802	0.789051
H	-4.091170	2.580769	1.140198
C	-2.466628	1.141111	1.231747
C	-2.301320	-2.271230	-0.615191
H	-1.658871	-2.695910	0.183941
C	-1.415481	-2.093953	-1.861849
H	-0.581191	-1.388541	-1.672516
H	-0.983464	-3.067231	-2.174004
H	-2.007264	-1.689334	-2.707965
C	-3.422570	-3.284839	-0.878373
H	-4.052313	-2.988660	-1.742793
H	-2.986360	-4.275386	-1.118042
H	-4.084974	-3.403536	0.002835
C	-1.645993	1.969537	2.212916
H	-0.720328	1.404173	2.442612
C	-1.208563	3.307467	1.595493
H	-2.080738	3.929293	1.304897
H	-0.600325	3.887299	2.319705
H	-0.587740	3.123909	0.691453
C	-2.404975	2.173577	3.535507
H	-2.692600	1.203535	3.989429
H	-1.770776	2.720121	4.263280
H	-3.330166	2.767345	3.383951
C	0.447854	-0.152447	0.610694
C	-0.527165	-1.553846	2.146097
H	-1.354793	-2.012370	2.695785
C	0.827873	-1.730340	2.235741
H	1.433913	-2.376210	2.878131
C	2.811270	-0.712515	1.092781
C	3.443472	-1.467778	0.076341
C	4.826893	-1.277042	-0.108376
H	5.348951	-1.842524	-0.895457
C	5.543777	-0.374927	0.689840
H	6.624287	-0.238945	0.528177
C	4.890973	0.355855	1.692562
H	5.463567	1.064991	2.309661
C	3.509403	0.201795	1.918822
C	2.661872	-2.411653	-0.827709
H	1.639951	-2.507180	-0.408339
C	3.277994	-3.818446	-0.874154
H	3.363247	-4.256315	0.141015
H	2.648011	-4.493069	-1.489064
H	4.290968	-3.805841	-1.326762
C	2.518764	-1.803948	-2.234568

```
  H       3.509389     -1.678720     -2.719663
  H       1.896708     -2.455849     -2.881904
  H       2.030280     -0.806095     -2.174123
  C       2.795828      1.036475      2.975351
  H       1.774856      0.622445      3.099943
  C       2.636820      2.490129      2.493294
  H       2.088792      2.523276      1.527244
  H       2.067954      3.087051      3.235878
  H       3.626048      2.972530      2.348283
  C       3.489821      0.964984      4.344120
  H       4.504629      1.412399      4.312461
  H       2.904096      1.526085      5.100455
  H       3.589606     -0.082818      4.693158
  C      -0.157323      2.010665     -2.646683
  C      -0.036602      1.052065     -3.678458
  C      -1.417786      2.559716     -2.315301
  C      -1.204253      0.546319     -4.266702
  H       0.954073      0.692861     -3.990488
  C      -2.563319      2.026789     -2.919476
  C      -2.486113      1.003841     -3.891195
  H      -1.112517     -0.234244     -5.038907
  H      -3.548563      2.416908     -2.619667
  C      -3.734784      0.393871     -4.472566
  H      -3.548538     -0.053259     -5.468821
  H      -4.114148     -0.419173     -3.813643
  H      -4.551014      1.137662     -4.566376
  Cl      1.384699      3.074037     -2.287320
  H      -1.496343      3.364660     -1.571546
TS1_EtOH.xyz
81
Coordinates from ORCA-job TS1 E -2016.074729707437
  Pd      0.753629      1.234983     -0.818897
  N      -0.737128     -0.596766      1.153343
  C      -2.029271     -0.114051      0.748552
  N       1.397844     -0.871521      1.294273
  C      -2.812615     -0.920882     -0.116801
  C      -4.058755     -0.409834     -0.525660
  H      -4.690101     -1.001255     -1.204330
  C      -4.505932      0.843990     -0.083362
  H      -5.481164      1.227018     -0.421375
  C      -3.722632      1.605545      0.791905
  H      -4.091839      2.581022      1.144370
  C      -2.467264      1.141147      1.234099
  C      -2.303892     -2.270760     -0.614677
  H      -1.661543     -2.695892      0.184267
  C      -1.418720     -2.095773     -1.862176
  H      -0.581219     -1.394185     -1.672880
  H      -0.990693     -3.070442     -2.175402
  H      -2.009783     -1.688324     -2.707481
  C      -3.426784     -3.282887     -0.876815
```

H	-4.056340	-2.986018	-1.741104
H	-2.991960	-4.274148	-1.115994
H	-4.088833	-3.399997	0.004854
C	-1.646782	1.968332	2.216466
H	-0.720005	1.403869	2.443906
C	-1.212763	3.308912	1.602255
H	-2.086653	3.930651	1.316742
H	-0.602927	3.886880	2.326633
H	-0.594880	3.129683	0.695362
C	-2.404725	2.167562	3.540412
H	-2.689442	1.195701	3.992250
H	-1.770816	2.713764	4.268691
H	-3.331398	2.759578	3.391067
C	0.447085	-0.150761	0.610015
C	-0.525970	-1.553128	2.145373
H	-1.352957	-2.012592	2.695309
C	0.829287	-1.728526	2.234355
H	1.436153	-2.374098	2.876315
C	2.811082	-0.708692	1.089817
C	3.442824	-1.463722	0.072831
C	4.825492	-1.270185	-0.115061
H	5.347178	-1.835728	-0.902383
C	5.542121	-0.365634	0.680761
H	6.621996	-0.227437	0.516660
C	4.889970	0.364186	1.684693
H	5.462469	1.074563	2.300507
C	3.509188	0.207172	1.914224
C	2.662058	-2.412358	-0.827000
H	1.639263	-2.504539	-0.409119
C	3.277636	-3.819818	-0.862333
H	3.359413	-4.250885	0.156018
H	2.649164	-4.498148	-1.474701
H	4.292027	-3.810151	-1.311828
C	2.522605	-1.815020	-2.238615
H	3.514201	-1.695893	-2.723254
H	1.900317	-2.470742	-2.881832
H	2.036091	-0.815862	-2.187421
C	2.797424	1.038597	2.974622
H	1.775053	0.627269	3.096461
C	2.643789	2.495910	2.502054
H	2.094871	2.537771	1.536936
H	2.077742	3.090061	3.248965
H	3.634668	2.975628	2.359519
C	3.490874	0.956158	4.343185
H	4.507700	1.399140	4.313930
H	2.907365	1.515393	5.102608
H	3.585682	-0.094158	4.685959
C	-0.158546	2.010587	-2.648426
C	-0.035376	1.052187	-3.680008
C	-1.419505	2.559902	-2.319560

```
  C      -1.201925      0.546116     -4.270421
  H       0.955871      0.692955     -3.990297
  C      -2.563893      2.026696     -2.925985
  C      -2.484608      1.003449     -3.897321
  H      -1.108606     -0.234646     -5.042222
  H      -3.549787      2.416821     -2.628251
  C      -3.732037      0.392891     -4.480632
  H      -3.543138     -0.057124     -5.475053
  H      -4.113402     -0.418105     -3.820384
  H      -4.547542      1.136948     -4.578364
  Cl      1.383870      3.076789     -2.287229
  H      -1.499853      3.364858     -1.575913
TS1_2-methoxyethanol.xyz
81
Coordinates from ORCA-job TS1 E -2016.074190017068
  Pd      0.754828      1.233859     -0.817921
  N      -0.737229     -0.597073      1.153558
  C      -2.029108     -0.114027      0.748453
  N       1.397599     -0.872850      1.294917
  C      -2.812675     -0.921029     -0.116493
  C      -4.058964     -0.410232     -0.525048
  H      -4.690561     -1.001966     -1.203198
  C      -4.505950      0.843764     -0.083141
  H      -5.481344      1.226634     -0.420867
  C      -3.722168      1.605777      0.791234
  H      -4.091076      2.581551      1.143177
  C      -2.466738      1.141516      1.233308
  C      -2.303461     -2.270533     -0.614786
  H      -1.661521     -2.696001      0.184333
  C      -1.417419     -2.094221     -1.861464
  H      -0.581242     -1.391241     -1.671464
  H      -0.987798     -3.068255     -2.174528
  H      -2.008306     -1.687377     -2.707147
  C      -3.425828     -3.282838     -0.878341
  H      -4.054909     -2.985639     -1.742876
  H      -2.990627     -4.273822     -1.118002
  H      -4.088536     -3.400830      0.002724
  C      -1.645787      1.969231      2.214816
  H      -0.719356      1.404420      2.442762
  C      -1.210595      3.308665      1.599025
  H      -2.083866      3.930508      1.311810
  H      -0.601022      3.887397      2.323002
  H      -0.591926      3.127525      0.693047
  C      -2.403590      2.170461      3.538527
  H      -2.689288      1.199364      3.991403
  H      -1.769287      2.716868      4.266313
  H      -3.329756      2.763145      3.388680
  C       0.447351     -0.151513      0.610410
  C      -0.526738     -1.553602      2.145603
  H      -1.354050     -2.012609      2.695394
```

C	0.828400	-1.729634	2.234857
H	1.434833	-2.375479	2.876924
C	2.811057	-0.710713	1.091361
C	3.443085	-1.465725	0.074592
C	4.826181	-1.273733	-0.111468
H	5.348085	-1.839138	-0.898723
C	5.542942	-0.370666	0.685839
H	6.623177	-0.233684	0.523145
C	4.890402	0.359541	1.689188
H	5.462943	1.069133	2.305837
C	3.509172	0.204154	1.916834
C	2.661892	-2.411603	-0.827734
H	1.639522	-2.505586	-0.409161
C	3.277683	-3.818744	-0.869241
H	3.361180	-4.253678	0.147330
H	2.648439	-4.494927	-1.483192
H	4.291370	-3.807473	-1.320283
C	2.520647	-1.808403	-2.236696
H	3.511770	-1.686064	-2.721508
H	1.898416	-2.461851	-2.882294
H	2.033239	-0.809869	-2.180362
C	2.796393	1.037272	2.975169
H	1.774777	0.624459	3.098435
C	2.639865	2.492623	2.497483
H	2.091610	2.529741	1.531752
H	2.072137	3.088243	3.241964
H	3.629860	2.973833	2.353794
C	3.490075	0.960717	4.343881
H	4.505809	1.406111	4.313401
H	2.905326	1.520936	5.101623
H	3.587574	-0.088255	4.690022
C	-0.158095	2.010626	-2.647519
C	-0.035171	1.052427	-3.679361
C	-1.419349	2.558688	-2.317543
C	-1.201734	0.545691	-4.269055
H	0.956154	0.694093	-3.990372
C	-2.563731	2.024811	-2.923182
C	-2.484444	1.001985	-3.894923
H	-1.108413	-0.234751	-5.041185
H	-3.549681	2.414042	-2.624506
C	-3.731915	0.390831	-4.477577
H	-3.543247	-0.059633	-5.471851
H	-4.113210	-0.419851	-3.816879
H	-4.547572	1.134692	-4.575738
Cl	1.383096	3.076366	-2.286611
H	-1.499603	3.363414	-1.573688

TS1_MeOH.xyz
81
Coordinates from ORCA-job TS1 E -2016.074965870864

Pd	0.753007	1.235696	-0.819180

N	-0.737151	-0.596530	1.153275
C	-2.029403	-0.113940	0.748611
N	1.397883	-0.870877	1.294033
C	-2.812688	-0.920710	-0.116874
C	-4.058749	-0.409527	-0.525878
H	-4.690014	-1.000826	-1.204735
C	-4.505965	0.844257	-0.083468
H	-5.481127	1.227361	-0.421600
C	-3.722837	1.605632	0.792135
H	-4.092138	2.581009	1.144783
C	-2.467511	1.141148	1.234399
C	-2.304237	-2.270794	-0.614516
H	-1.661882	-2.695865	0.184445
C	-1.419233	-2.096448	-1.862232
H	-0.581095	-1.395582	-1.673095
H	-0.991976	-3.071436	-2.175501
H	-2.010194	-1.688631	-2.707444
C	-3.427414	-3.282724	-0.876248
H	-4.056990	-2.985917	-1.740539
H	-2.992812	-4.274146	-1.115159
H	-4.089347	-3.399399	0.005562
C	-1.647213	1.968119	2.217108
H	-0.720232	1.403858	2.444232
C	-1.213865	3.309253	1.603600
H	-2.088103	3.930983	1.319156
H	-0.603687	3.886804	2.328028
H	-0.596600	3.130921	0.696115
C	-2.405122	2.166354	3.541230
H	-2.689236	1.194114	3.992626
H	-1.771391	2.712542	4.269674
H	-3.332113	2.757951	3.392199
C	0.446922	-0.150308	0.609917
C	-0.525730	-1.552884	2.145239
H	-1.352588	-2.012568	2.695203
C	0.829573	-1.728030	2.234112
H	1.436606	-2.373534	2.875997
C	2.811041	-0.707806	1.089222
C	3.442658	-1.462897	0.072179
C	4.825151	-1.268742	-0.116480
H	5.346743	-1.834382	-0.903804
C	5.541737	-0.363558	0.678695
H	6.621464	-0.224879	0.514005
C	4.889762	0.366130	1.682856
H	5.462257	1.076824	2.298321
C	3.509164	0.208464	1.913178
C	2.662056	-2.412697	-0.826580
H	1.639154	-2.504293	-0.408850
C	3.277694	-3.820212	-0.859570
H	3.359004	-4.249746	0.159462
H	2.649494	-4.499450	-1.471210

```
  H       4.292284     -3.811064     -1.308626
  C       2.523100     -1.817698     -2.239226
  H       3.514822     -1.699813     -2.723910
  H       1.900741     -2.474357     -2.881411
  H       2.036898     -0.818324     -2.190033
  C       2.797865      1.039171      2.974473
  H       1.775140      0.628543      3.095655
  C       2.645601      2.497345      2.504148
  H       2.096478      2.541294      1.539261
  H       2.080256      3.090874      3.252075
  H       3.636899      2.976362      2.362194
  C       3.491166      0.954098      4.342984
  H       4.508517      1.395912      4.314278
  H       2.908242      1.512974      5.103119
  H       3.584672     -0.096806      4.684297
  C      -0.158652      2.010495     -2.648878
  C      -0.035178      1.051946     -3.680263
  C      -1.419568      2.560264     -2.320668
  C      -1.201613      0.546017     -4.271080
  H       0.956099      0.692389     -3.990110
  C      -2.563844      2.027208     -2.927524
  C      -2.484364      1.003714     -3.898601
  H      -1.108144     -0.234931     -5.042672
  H      -3.549779      2.417666     -2.630344
  C      -3.731666      0.393272     -4.482278
  H      -3.542364     -0.057233     -5.476393
  H      -4.113473     -0.417342     -3.821827
  H      -4.546930      1.137502     -4.580628
  Cl      1.384179      3.077072     -2.287465
  H      -1.500114      3.365369     -1.577180
TS1_Anisole.xyz
81
Coordinates from ORCA-job TS1 E -2016.070330345586
  Pd      0.762265      1.228787     -0.809763
  N      -0.737791     -0.596853      1.156487
  C      -2.028074     -0.111541      0.749712
  N       1.396220     -0.880598      1.298851
  C      -2.813555     -0.919799     -0.112034
  C      -4.061043     -0.410752     -0.517921
  H      -4.694791     -1.004796     -1.191926
  C      -4.506465      0.844532     -0.078828
  H      -5.483164      1.226205     -0.414123
  C      -3.718925      1.609835      0.788800
  H      -4.085621      2.587678      1.137183
  C      -2.463012      1.146426      1.229766
  C      -2.301470     -2.266948     -0.613137
  H      -1.663029     -2.695280      0.187446
  C      -1.408775     -2.082053     -1.853702
  H      -0.581166     -1.370395     -1.658544
  H      -0.968993     -3.052016     -2.165559
```

H	-1.997643	-1.678731	-2.702198
C	-3.420664	-3.279730	-0.887185
H	-4.045501	-2.979962	-1.753996
H	-2.983327	-4.269097	-1.129693
H	-4.088716	-3.403265	-0.010885
C	-1.638742	1.977689	2.205330
H	-0.715306	1.409834	2.437514
C	-1.194370	3.308583	1.578257
H	-2.062913	3.931661	1.279353
H	-0.585958	3.892353	2.299084
H	-0.570635	3.113444	0.678619
C	-2.395863	2.193857	3.526984
H	-2.689197	1.228554	3.987350
H	-1.758703	2.741548	4.251318
H	-3.318024	2.791743	3.373147
C	0.449081	-0.154270	0.614127
C	-0.531353	-1.555498	2.147530
H	-1.360569	-2.011567	2.696631
C	0.822985	-1.736460	2.237451
H	1.426644	-2.384964	2.879250
C	2.811045	-0.723145	1.100130
C	3.443795	-1.475867	0.082405
C	4.829202	-1.292939	-0.093405
H	5.351679	-1.855575	-0.882093
C	5.547614	-0.401130	0.714420
H	6.629831	-0.271372	0.559533
C	4.893903	0.328898	1.716744
H	5.467381	1.031999	2.339696
C	3.510245	0.183301	1.933749
C	2.659331	-2.403088	-0.836108
H	1.641184	-2.512202	-0.410507
C	3.278797	-3.806207	-0.922521
H	3.378448	-4.267752	0.080817
H	2.643288	-4.468444	-1.545159
H	4.285945	-3.781960	-1.387512
C	2.501113	-1.759376	-2.225406
H	3.487474	-1.611978	-2.712947
H	1.879472	-2.398113	-2.886169
H	2.005125	-0.767163	-2.132062
C	2.792461	1.024728	2.981781
H	1.776257	0.601631	3.115499
C	2.615821	2.467908	2.474803
H	2.071090	2.476623	1.506098
H	2.037566	3.070630	3.205468
H	3.599467	2.959729	2.323491
C	3.489686	0.983298	4.349735
H	4.497973	1.444686	4.310896
H	2.897825	1.547571	5.098896
H	3.605592	-0.057380	4.714833
C	-0.159573	2.010034	-2.640305

```
  C      -0.033066      1.055059     -3.674849
  C      -1.425374      2.543803     -2.303462
  C      -1.196854      0.539672     -4.261772
  H       0.960585      0.707540     -3.990210
  C      -2.566609      2.001485     -2.905918
  C      -2.481913      0.983182     -3.881741
  H      -1.099374     -0.237190     -5.037030
  H      -3.554817      2.380221     -2.601592
  C      -3.726635      0.364358     -4.462478
  H      -3.533674     -0.098127     -5.450429
  H      -4.112990     -0.437566     -3.793932
  H      -4.541752      1.106941     -4.575575
  Cl      1.368774      3.079271     -2.279047
  H      -1.509179      3.345852     -1.557462
TS1_ethyleneglycol.xyz
81
Coordinates from ORCA-job TS1 E -2016.075110321636
  Pd      0.752825      1.235748     -0.819489
  N      -0.737077     -0.596405      1.153398
  C      -2.029386     -0.114068      0.748584
  N       1.397980     -0.870531      1.294094
  C      -2.812270     -0.920858     -0.117241
  C      -4.058246     -0.409759     -0.526657
  H      -4.689163     -1.001024     -1.205878
  C      -4.505801      0.843891     -0.084193
  H      -5.480869      1.226937     -0.422659
  C      -3.723141      1.605206      0.791905
  H      -4.092748      2.580442      1.144631
  C      -2.467885      1.140843      1.234509
  C      -2.303621     -2.270912     -0.614758
  H      -1.660298     -2.695323      0.183758
  C      -1.419914     -2.096755     -1.863418
  H      -0.581688     -1.395727     -1.675256
  H      -0.992756     -3.071748     -2.176799
  H      -2.011794     -1.689336     -2.708194
  C      -3.426633     -3.283454     -0.874892
  H      -4.057246     -2.987324     -1.738655
  H      -2.991825     -4.274797     -1.113761
  H      -4.087563     -3.399982      0.007688
  C      -1.648007      1.967759      2.217619
  H      -0.721112      1.403494      2.445109
  C      -1.214518      3.309000      1.604412
  H      -2.088721      3.930712      1.319826
  H      -0.604572      3.886469      2.329104
  H      -0.597034      3.130893      0.697028
  C      -2.406451      2.165882      3.541452
  H      -2.690692      1.193589      3.992652
  H      -1.773009      2.712030      4.270175
  H      -3.333389      2.757470      3.392066
  C       0.446905     -0.150224      0.609897
```

C	-0.525518	-1.552523	2.145552
H	-1.352314	-2.012220	2.695606
C	0.829812	-1.727533	2.234383
H	1.436936	-2.372868	2.876358
C	2.811082	-0.707391	1.088975
C	3.442514	-1.462545	0.071849
C	4.824898	-1.268081	-0.117367
H	5.346355	-1.833803	-0.904728
C	5.541554	-0.362549	0.677368
H	6.621187	-0.223632	0.512251
C	4.889786	0.367101	1.681706
H	5.462351	1.077971	2.296908
C	3.509309	0.209110	1.912597
C	2.661932	-2.413060	-0.826183
H	1.638880	-2.503974	-0.408689
C	3.277306	-3.820749	-0.857302
H	3.357999	-4.249133	0.162262
H	2.649264	-4.500541	-1.468487
H	4.292127	-3.812235	-1.305850
C	2.523623	-1.819783	-2.239612
H	3.515516	-1.702908	-2.724188
H	1.901230	-2.477061	-2.881126
H	2.037752	-0.820197	-2.191939
C	2.798359	1.039478	2.974404
H	1.775507	0.629140	3.095466
C	2.646589	2.498048	2.505155
H	2.097137	2.542978	1.540506
H	2.081722	3.091265	3.253687
H	3.638036	2.976770	2.363249
C	3.491828	0.953233	4.342773
H	4.509384	1.394581	4.314148
H	2.909256	1.511895	5.103334
H	3.584857	-0.097939	4.683386
C	-0.158615	2.010549	-2.648993
C	-0.035362	1.052036	-3.680429
C	-1.419290	2.560935	-2.320959
C	-1.201943	0.546666	-4.271458
H	0.955788	0.692015	-3.990160
C	-2.563733	2.028429	-2.928047
C	-2.484556	1.004927	-3.899146
H	-1.108706	-0.234295	-5.043063
H	-3.549541	2.419337	-2.631031
C	-3.732010	0.395014	-4.483033
H	-3.543038	-0.054337	-5.477734
H	-4.113366	-0.416440	-3.823359
H	-4.547407	1.139252	-4.580126
Cl	1.384867	3.076903	-2.287545
H	-1.499626	3.366027	-1.577423

TS1_Ethylacetate.xyz
81

Coordinates from ORCA-job TS1 E -2016.071803383416

Pd	0.759584	1.229898	-0.813257
N	-0.737592	-0.597559	1.155241
C	-2.028503	-0.112945	0.749389
N	1.396720	-0.878195	1.297203
C	-2.813536	-0.920571	-0.113466
C	-4.060609	-0.410707	-0.520053
H	-4.693739	-1.003748	-1.195561
C	-4.506391	0.844112	-0.079702
H	-5.482619	1.226380	-0.415693
C	-3.720000	1.608019	0.790378
H	-4.087326	2.585116	1.140226
C	-2.464215	1.144120	1.231446
C	-2.302698	-2.268556	-0.613809
H	-1.663060	-2.696093	0.186175
C	-1.412382	-2.086627	-1.856560
H	-0.581523	-1.378310	-1.663066
H	-0.976446	-3.058069	-2.169117
H	-2.001925	-1.681695	-2.703926
C	-3.423197	-3.280941	-0.884298
H	-4.049533	-2.981767	-1.750199
H	-2.986770	-4.270895	-1.126026
H	-4.089317	-3.402589	-0.006280
C	-1.640828	1.973963	2.209021
H	-0.716295	1.407121	2.439435
C	-1.199856	3.307957	1.585940
H	-2.070184	3.930648	1.291560
H	-0.590776	3.889816	2.307774
H	-0.578225	3.117850	0.683803
C	-2.397876	2.184748	3.531621
H	-2.688379	1.217338	3.989342
H	-1.761535	2.731934	4.257050
H	-3.321493	2.780810	3.379439
C	0.448386	-0.153863	0.612498
C	-0.529572	-1.555295	2.146772
H	-1.358047	-2.012439	2.696198
C	0.825078	-1.734323	2.236436
H	1.429839	-2.381699	2.878414
C	2.811035	-0.718702	1.096946
C	3.443954	-1.472462	0.079992
C	4.828563	-1.286065	-0.099181
H	5.351137	-1.849808	-0.887079
C	5.546006	-0.389792	0.704795
H	6.627546	-0.257282	0.547376
C	4.892267	0.340576	1.707004
H	5.465086	1.046387	2.327586
C	3.509409	0.191289	1.927446
C	2.660953	-2.406580	-0.832820
H	1.641358	-2.510682	-0.409620
C	3.279671	-3.811022	-0.903561

```
  H       3.373631     -4.263302      0.104518
  H       2.646670     -4.478320     -1.523344
  H       4.289205     -3.790971     -1.363537
  C       2.508390     -1.776941     -2.229109
  H       3.496369     -1.637863     -2.715834
  H       1.886805     -2.421084     -2.884646
  H       2.014972     -0.782571     -2.148608
  C       2.792942      1.030094      2.978569
  H       1.774392      0.611316      3.107515
  C       2.624862      2.477871      2.482008
  H       2.079811      2.497119      1.513759
  H       2.049978      3.078361      3.217108
  H       3.611239      2.965361      2.334532
  C       3.487726      0.975540      4.347476
  H       4.499102      1.430418      4.312444
  H       2.898224      1.538860      5.099215
  H       3.595911     -0.068276      4.705893
  C      -0.158564      2.010004     -2.643207
  C      -0.033791      1.053814     -3.676805
  C      -1.422515      2.549498     -2.308822
  C      -1.198839      0.541992     -4.264635
  H       0.958873      0.701956     -3.990635
  C      -2.565164      2.010651     -2.912335
  C      -2.482877      0.990622     -3.886640
  H      -1.103213     -0.236243     -5.038732
  H      -3.552406      2.393575     -2.609988
  C      -3.728865      0.374923     -4.467895
  H      -3.537850     -0.082790     -5.458430
  H      -4.113152     -0.430474     -3.802391
  H      -4.544184      1.118091     -4.574910
  Cl      1.374871      3.077444     -2.282200
  H      -1.504695      3.352525     -1.563562
I2_n-BuOAc.xyz
81
Coordinates from ORCA-job I2 E -2016.123847287107
  Pd      0.492883     -0.061743     -1.361055
  N      -0.383156      0.954493      1.434543
  C      -1.691408      1.447510      1.092114
  N       1.587221      0.063669      1.335213
  C      -2.823891      0.754814      1.586084
  C      -4.091544      1.260971      1.243680
  H      -4.993073      0.745579      1.607067
  C      -4.221915      2.399585      0.437777
  H      -5.223292      2.772677      0.173539
  C      -3.082561      3.066862     -0.029390
  H      -3.195112      3.965544     -0.654922
  C      -1.788935      2.612175      0.294082
  C      -2.699052     -0.476147      2.475947
  H      -1.649154     -0.829977      2.416196
  C      -3.592232     -1.635141      2.009261
```

H	-3.384743	-1.903945	0.956415
H	-3.410748	-2.530317	2.638398
H	-4.669300	-1.383869	2.097254
C	-2.993777	-0.114417	3.944089
H	-4.044750	0.223413	4.059829
H	-2.846032	-0.997371	4.599097
H	-2.338290	0.699916	4.311875
C	-0.568719	3.385135	-0.190125
H	0.335903	2.836290	0.138772
C	-0.515613	3.459323	-1.724262
H	-1.395068	3.992860	-2.139861
H	0.395139	3.999805	-2.053135
H	-0.482902	2.440462	-2.164858
C	-0.516463	4.782524	0.452288
H	-0.514226	4.716310	1.559214
H	0.401989	5.318821	0.136898
H	-1.388230	5.399726	0.150902
C	0.471461	0.313183	0.577204
C	0.200234	1.120596	2.690438
H	-0.321129	1.632395	3.503995
C	1.445806	0.555144	2.628691
H	2.241979	0.469099	3.373933
C	2.770184	-0.557828	0.794688
C	2.868666	-1.970689	0.818856
C	4.035459	-2.542102	0.277901
H	4.151793	-3.635315	0.276242
C	5.047113	-1.739248	-0.268174
H	5.948803	-2.208843	-0.690074
C	4.913556	-0.345221	-0.290614
H	5.707890	0.271001	-0.736854
C	3.769237	0.279073	0.242441
C	1.762894	-2.828515	1.419471
H	0.812790	-2.266872	1.294724
C	1.989400	-3.032031	2.930210
H	2.040393	-2.066793	3.471192
H	1.162778	-3.626790	3.369942
H	2.939700	-3.575639	3.112177
C	1.593846	-4.175502	0.703841
H	2.447456	-4.856905	0.897364
H	0.678136	-4.683845	1.066387
H	1.505902	-4.044387	-0.393568
C	3.580175	1.788159	0.149647
H	2.807832	2.080507	0.890682
C	3.044594	2.164918	-1.242412
H	2.065261	1.671453	-1.456146
H	2.868934	3.256963	-1.321486
H	3.748552	1.858420	-2.042346
C	4.854235	2.577083	0.485171
H	5.651301	2.415826	-0.269117
H	4.634113	3.663393	0.502622

```
  H       5.257407      2.289990      1.476923
  C      -1.250739     -0.972188     -1.313837
  C      -1.312631     -2.359288     -1.094465
  C      -2.414226     -0.268301     -1.668798
  C      -2.535922     -3.036620     -1.253742
  H      -0.412857     -2.927896     -0.822439
  C      -3.627910     -0.960655     -1.819428
  C      -3.714762     -2.353497     -1.615499
  H      -2.568708     -4.126081     -1.088054
  H      -4.531796     -0.395244     -2.099942
  C      -5.034000     -3.076179     -1.729356
  H      -4.895350     -4.141191     -2.001674
  H      -5.581563     -3.055158     -0.761548
  H      -5.693896     -2.604837     -2.484625
  Cl      0.890799     -0.432749     -3.648937
  H      -2.387575      0.817811     -1.835972
I2_Ethylacetate.xyz
81
Coordinates from ORCA-job I2 E -2016.125114793366
  Pd      0.494079     -0.057015     -1.361658
  N      -0.383610      0.954769      1.434763
  C      -1.691732      1.448433      1.092561
  N       1.586179      0.062488      1.335195
  C      -2.824445      0.755629      1.585999
  C      -4.092000      1.262232      1.243703
  H      -4.993675      0.746928      1.606902
  C      -4.222092      2.401433      0.438519
  H      -5.223384      2.774928      0.174481
  C      -3.082527      3.068988     -0.027834
  H      -3.194826      3.968368     -0.652424
  C      -1.789002      2.613879      0.295618
  C      -2.700235     -0.475577      2.475628
  H      -1.650295     -0.829432      2.416882
  C      -3.593140     -1.634490      2.008088
  H      -3.384550     -1.903660      0.955525
  H      -3.412483     -2.529541      2.637635
  H      -4.670236     -1.382911      2.094780
  C      -2.996363     -0.114082      3.943563
  H      -4.047637      0.223163      4.058323
  H      -2.848709     -0.997120      4.598471
  H      -2.341468      0.700471      4.311859
  C      -0.568711      3.387470     -0.187365
  H       0.335977      2.838860      0.141692
  C      -0.515104      3.462675     -1.721438
  H      -1.394415      3.996566     -2.136887
  H       0.395932      4.003070     -2.049671
  H      -0.482691      2.443920     -2.162399
  C      -0.517196      4.784548      0.455760
  H      -0.515632      4.717585      1.562632
  H       0.401468      5.321012      0.141282
```

H	-1.388886	5.401668	0.153975
C	0.471217	0.314509	0.577161
C	0.199096	1.118043	2.691366
H	-0.322246	1.628923	3.505552
C	1.444254	0.551693	2.629467
H	2.239888	0.463801	3.375103
C	2.768727	-0.559347	0.794072
C	2.868061	-1.972118	0.820520
C	4.034136	-2.543854	0.278181
H	4.151207	-3.637009	0.278545
C	5.044287	-1.741359	-0.271324
H	5.945477	-2.211185	-0.694076
C	4.910103	-0.347357	-0.295490
H	5.703479	0.268646	-0.743772
C	3.766568	0.277193	0.239047
C	1.764438	-2.829657	1.425436
H	0.813814	-2.268091	1.304193
C	1.996861	-3.032726	2.935361
H	2.050808	-2.067381	3.475829
H	1.171583	-3.626839	3.378452
H	2.947505	-3.577020	3.113443
C	1.593011	-4.177094	0.711159
H	2.448018	-4.857718	0.901136
H	0.679335	-4.685837	1.078224
H	1.500037	-4.046740	-0.385954
C	3.577287	1.786182	0.145717
H	2.805507	2.078917	0.887131
C	3.040679	2.162255	-1.246022
H	2.060187	1.669733	-1.457961
H	2.865266	3.254268	-1.325789
H	3.743428	1.854548	-2.046572
C	4.851605	2.575339	0.479746
H	5.648170	2.413537	-0.274955
H	4.631258	3.661604	0.496705
H	5.255180	2.288822	1.471488
C	-1.248351	-0.969954	-1.314484
C	-1.309579	-2.357080	-1.094724
C	-2.412165	-0.266878	-1.670402
C	-2.532209	-3.035371	-1.255612
H	-0.410012	-2.924959	-0.820337
C	-3.625147	-0.960183	-1.822731
C	-3.711124	-2.353172	-1.619041
H	-2.564597	-4.124769	-1.089442
H	-4.529248	-0.395431	-2.103906
C	-5.029678	-3.076845	-1.734299
H	-4.889943	-4.141721	-2.006563
H	-5.577896	-3.056354	-0.766866
H	-5.689351	-2.605683	-2.489868
Cl	0.893442	-0.428863	-3.651922
H	-2.386399	0.819467	-1.836514

```
I2_Benzylalcohol.xyz
81
Coordinates from ORCA-job I2 E -2016.128586739837
  Pd      0.494425     -0.028500     -1.367009
  N      -0.383920      0.961060      1.434363
  C      -1.692128      1.455795      1.093681
  N       1.582557      0.061283      1.333065
  C      -2.824574      0.760727      1.584900
  C      -4.092474      1.267096      1.242982
  H      -4.993932      0.750561      1.605109
  C      -4.223129      2.408233      0.440446
  H      -5.224635      2.781664      0.176995
  C      -3.083789      3.078384     -0.023091
  H      -3.196456      3.979950     -0.644494
  C      -1.790018      2.623613      0.300293
  C      -2.700454     -0.471662      2.473015
  H      -1.649766     -0.823786      2.417427
  C      -3.590246     -1.631616      2.001849
  H      -3.377488     -1.900973      0.950100
  H      -3.410857     -2.526298      2.632264
  H      -4.667911     -1.381069      2.084252
  C      -3.001720     -0.112278      3.940463
  H      -4.054375      0.221680      4.052291
  H      -2.853232     -0.995747      4.594577
  H      -2.349851      0.703639      4.310965
  C      -0.570246      3.400969     -0.177866
  H       0.335110      2.851951      0.148471
  C      -0.517084      3.485136     -1.711455
  H      -1.394463      4.025078     -2.123189
  H       0.396349      4.023161     -2.036963
  H      -0.490170      2.468421     -2.157698
  C      -0.520431      4.794543      0.472872
  H      -0.518106      4.720871      1.579286
  H       0.397582      5.333676      0.161075
  H      -1.393259      5.411793      0.174596
  C       0.470228      0.322894      0.575350
  C       0.197310      1.114823      2.692816
  H      -0.323074      1.623246      3.509258
  C       1.440303      0.543867      2.629839
  H       2.234421      0.449019      3.376330
  C       2.762524     -0.564938      0.791230
  C       2.860089     -1.977730      0.824273
  C       4.023733     -2.553622      0.280537
  H       4.139847     -3.646917      0.286686
  C       5.033177     -1.754980     -0.276126
  H       5.932690     -2.227977     -0.699034
  C       4.901139     -0.360677     -0.305666
  H       5.694529      0.252446     -0.757902
  C       3.760264      0.267740      0.230231
  C       1.758294     -2.831246      1.438067
```

```
  H       0.807873     -2.268416      1.320777
  C       1.998964     -3.030240      2.947319
  H       2.058509     -2.063647      3.484777
  H       1.174621     -3.621130      3.396361
  H       2.949182     -3.576712      3.120918
  C       1.581291     -4.181398      0.730008
  H       2.435402     -4.862927      0.920568
  H       0.667355     -4.686038      1.102062
  H       1.485606     -4.056393     -0.367568
  C       3.573630      1.776822      0.134275
  H       2.807402      2.073439      0.879753
  C       3.029193      2.150881     -1.254633
  H       2.041766      1.665819     -1.456486
  H       2.858423      3.243343     -1.337538
  H       3.722999      1.835245     -2.059868
  C       4.851894      2.564239      0.457194
  H       5.643036      2.398686     -0.302371
  H       4.633287      3.650831      0.473016
  H       5.260820      2.279209      1.447122
  C      -1.238825     -0.960059     -1.318997
  C      -1.289154     -2.348018     -1.101041
  C      -2.409097     -0.265666     -1.672063
  C      -2.506314     -3.036163     -1.262846
  H      -0.385760     -2.909060     -0.825041
  C      -3.616359     -0.968773     -1.826391
  C      -3.690828     -2.362998     -1.625379
  H      -2.529965     -4.125807     -1.096778
  H      -4.525510     -0.410931     -2.105179
  C      -5.003581     -3.097017     -1.740665
  H      -4.855519     -4.160398     -2.014278
  H      -5.550332     -3.082133     -0.772342
  H      -5.668087     -2.629590     -2.494297
  Cl      0.888859     -0.385987     -3.668321
  H      -2.393000      0.821826     -1.832963
I2_2-pentanol.xyz
81
Coordinates from ORCA-job I2 E -2016.128745354361
  Pd      0.493989     -0.025569     -1.367614
  N      -0.383827      0.961944      1.434256
  C      -1.692067      1.456686      1.093709
  N       1.582267      0.061342      1.332807
  C      -2.824432      0.761273      1.584647
  C      -4.092395      1.267499      1.242727
  H      -4.993797      0.750730      1.604665
  C      -4.223178      2.408821      0.440463
  H      -5.224728      2.782142      0.177018
  C      -3.083911      3.079332     -0.022760
  H      -3.196680      3.981083     -0.643878
  C      -1.790088      2.624713      0.300654
  C      -2.700179     -0.471261      2.472554
```

H	-1.649402	-0.823153	2.417135
C	-3.589622	-1.631357	2.001065
H	-3.376513	-1.900663	0.949371
H	-3.410252	-2.526019	2.631513
H	-4.667355	-1.381000	2.083145
C	-3.001808	-0.112166	3.940000
H	-4.054577	0.221489	4.051671
H	-2.853197	-0.995704	4.593992
H	-2.350202	0.703860	4.310718
C	-0.570401	3.402505	-0.177004
H	0.335031	2.853393	0.148958
C	-0.517324	3.487742	-1.710531
H	-1.394451	4.028449	-2.121804
H	0.396384	4.025488	-2.035737
H	-0.491078	2.471316	-2.157479
C	-0.520762	4.795650	0.474662
H	-0.518340	4.721210	1.581023
H	0.397149	5.335133	0.163169
H	-1.393730	5.412922	0.176836
C	0.470180	0.323836	0.575096
C	0.197311	1.114947	2.692833
H	-0.322937	1.623254	3.509438
C	1.440068	0.543488	2.629760
H	2.234080	0.448060	3.376292
C	2.761946	-0.565452	0.791021
C	2.859160	-1.978254	0.824711
C	4.022569	-2.554678	0.281000
H	4.138448	-3.647998	0.287672
C	5.032099	-1.756539	-0.276239
H	5.931430	-2.229955	-0.699071
C	4.900413	-0.362204	-0.306372
H	5.693896	0.250532	-0.758971
C	3.759800	0.266720	0.229493
C	1.757280	-2.831246	1.439091
H	0.806947	-2.268294	1.321630
C	1.998021	-3.029498	2.948437
H	2.057592	-2.062660	3.485433
H	1.173666	-3.620150	3.397769
H	2.948227	-3.575923	3.122247
C	1.579997	-4.181809	0.731874
H	2.433901	-4.863428	0.923036
H	0.665858	-4.685934	1.104129
H	1.484535	-4.057578	-0.365813
C	3.573580	1.775829	0.133153
H	2.807766	2.072921	0.878860
C	3.028729	2.149719	-1.255613
H	2.040670	1.665507	-1.456774
H	2.858639	3.242255	-1.338908
H	3.721789	1.833177	-2.061130
C	4.852239	2.562947	0.455256

```
  H       5.642989      2.396922     -0.304613
  H       4.633950      3.649603      0.470887
  H       5.261482      2.278044      1.445086
  C      -1.238212     -0.959190     -1.319419
  C      -1.287137     -2.347264     -1.101859
  C      -2.409306     -0.265846     -1.671903
  C      -2.503643     -3.036592     -1.263604
  H      -0.383156     -2.907487     -0.826120
  C      -3.615890     -0.970126     -1.826265
  C      -3.688908     -2.364504     -1.625704
  H      -2.526176     -4.126297     -1.097780
  H      -4.525694     -0.413127     -2.104614
  C      -5.000950     -3.099799     -1.740904
  H      -4.851886     -4.162937     -2.014913
  H      -5.547422     -3.085800     -0.772412
  H      -5.666150     -2.632759     -2.494163
  Cl      0.886612     -0.379876     -3.670162
  H      -2.394368      0.821755     -1.832266
I2_PrOAc.xyz
81
Coordinates from ORCA-job I2 E -2016.124572582912
  Pd      0.493470     -0.057072     -1.361883
  N      -0.383187      0.955583      1.434489
  C      -1.691507      1.448655      1.092336
  N       1.586685      0.063611      1.334871
  C      -2.823860      0.755470      1.585989
  C      -4.091635      1.261437      1.243640
  H      -4.993071      0.745776      1.606905
  C      -4.222233      2.400329      0.438123
  H      -5.223692      2.773288      0.173980
  C      -3.082993      3.068170     -0.028594
  H      -3.195716      3.967201     -0.653601
  C      -1.789271      2.613705      0.294899
  C      -2.698890     -0.475620      2.475677
  H      -1.648822     -0.829058      2.416506
  C      -3.591451     -1.634910      2.008472
  H      -3.383299     -1.903774      0.955756
  H      -3.410038     -2.529976      2.637783
  H      -4.668634     -1.383912      2.095795
  C      -2.994539     -0.114175      3.943712
  H      -4.045795      0.222969      4.058907
  H      -2.846549     -0.997150      4.598633
  H      -2.339655      0.700490      4.311808
  C      -0.569222      3.387450     -0.188451
  H       0.335577      2.838726      0.140132
  C      -0.516049      3.463006     -1.722513
  H      -1.395180      3.997464     -2.137620
  H       0.395120      4.003100     -2.050878
  H      -0.484162      2.444426     -2.163869
  C      -0.517546      4.784334      0.455094
```

H	-0.515231	4.717091	1.561953
H	0.400730	5.321194	0.140151
H	-1.389598	5.401360	0.154163
C	0.471382	0.314752	0.576931
C	0.199921	1.120020	2.690735
H	-0.321309	1.631347	3.504696
C	1.445160	0.553857	2.628809
H	2.241069	0.466627	3.374215
C	2.769155	-0.558710	0.794186
C	2.867191	-1.971592	0.819443
C	4.033534	-2.543811	0.278256
H	4.149614	-3.637062	0.277586
C	5.045159	-1.741677	-0.269004
H	5.946546	-2.211882	-0.690896
C	4.912088	-0.347577	-0.292352
H	5.706493	0.268093	-0.739235
C	3.768265	0.277467	0.240939
C	1.761579	-2.828657	1.421432
H	0.811553	-2.266766	1.297136
C	1.989105	-3.031352	2.932141
H	2.040655	-2.065848	3.472563
H	1.162618	-3.625714	3.372650
H	2.939400	-3.575118	3.113655
C	1.591691	-4.176154	0.706911
H	2.445055	-4.857738	0.900838
H	0.675771	-4.683687	1.070066
H	1.503631	-4.046030	-0.390622
C	3.579738	1.786582	0.147728
H	2.808598	2.079661	0.889701
C	3.042378	2.162968	-1.243690
H	2.061633	1.670955	-1.455234
H	2.867680	3.255116	-1.323330
H	3.744476	1.854903	-2.044657
C	4.854633	2.575154	0.480895
H	5.650494	2.413255	-0.274525
H	4.634825	3.661522	0.498261
H	5.259050	2.288276	1.472193
C	-1.248719	-0.970452	-1.314576
C	-1.308990	-2.357677	-1.095393
C	-2.413174	-0.267888	-1.669233
C	-2.531445	-3.036524	-1.254893
H	-0.408703	-2.925251	-0.822885
C	-3.625977	-0.961756	-1.820290
C	-3.711108	-2.354790	-1.616681
H	-2.562919	-4.126005	-1.089067
H	-4.530618	-0.397399	-2.100515
C	-5.029480	-3.079027	-1.730548
H	-4.889560	-4.143929	-2.002620
H	-5.576981	-3.058441	-0.762704
H	-5.689954	-2.608470	-2.485801

```
  Cl      0.891626     -0.427044     -3.651488
  H      -2.388020      0.818426     -1.835524
I2_THF.xyz
81
Coordinates from ORCA-job I2 E -2016.126382080620
  Pd      0.492974     -0.042293     -1.364765
  N      -0.383362      0.958761      1.434121
  C      -1.691608      1.452564      1.092560
  N       1.584612      0.062462      1.333869
  C      -2.823993      0.758539      1.585131
  C      -4.091821      1.264655      1.242904
  H      -4.993275      0.748626      1.605681
  C      -4.222427      2.404466      0.438593
  H      -5.223902      2.777600      0.174692
  C      -3.083134      3.073307     -0.026756
  H      -3.195818      3.973422     -0.650227
  C      -1.789401      2.618742      0.296773
  C      -2.699393     -0.473082      2.474188
  H      -1.648938     -0.825683      2.417020
  C      -3.590230     -1.632933      2.004930
  H      -3.379263     -1.902296      0.952866
  H      -3.410027     -2.527618      2.635122
  H      -4.667726     -1.382258      2.089211
  C      -2.998134     -0.112576      3.941848
  H      -4.050169      0.222776      4.055227
  H      -2.849789     -0.995680      4.596504
  H      -2.344973      0.702905      4.311134
  C      -0.569419      3.394268     -0.183826
  H       0.335560      2.844301      0.142049
  C      -0.517233      3.476618     -1.717535
  H      -1.395156      4.015459     -2.129538
  H       0.395389      4.015336     -2.044154
  H      -0.489158      2.459749     -2.163135
  C      -0.517465      4.788387      0.465654
  H      -0.514204      4.716063      1.572176
  H       0.400597      5.326594      0.152386
  H      -1.389976      5.406327      0.167896
  C       0.470832      0.318847      0.575937
  C       0.198834      1.118111      2.691428
  H      -0.321910      1.628268      3.506490
  C       1.442843      0.549289      2.629103
  H       2.237886      0.458360      3.375038
  C       2.765882     -0.561928      0.792882
  C       2.863530     -1.974770      0.821999
  C       4.028713     -2.548895      0.280037
  H       4.144696     -3.642181      0.282714
  C       5.039543     -1.748572     -0.271484
  H       5.940123     -2.220235     -0.693533
  C       4.907046     -0.354373     -0.298083
  H       5.701099      0.259956     -0.747463
```

```
  C       3.764510      0.272423      0.236019
  C       1.759247     -2.829967      1.429029
  H       0.809136     -2.267677      1.306965
  C       1.991585     -3.030275      2.939366
  H       2.045634     -2.064038      3.478138
  H       1.166054     -3.623425      3.383257
  H       2.942052     -3.574578      3.118337
  C       1.586626     -4.179013      0.717966
  H       2.440234     -4.860443      0.911252
  H       0.671376     -4.685087      1.084818
  H       1.495829     -4.051751     -0.379719
  C       3.576951      1.781515      0.141109
  H       2.808241      2.076505      0.884772
  C       3.036085      2.156391     -1.249185
  H       2.051276      1.669060     -1.455233
  H       2.864952      3.248896     -1.331303
  H       3.733312      1.842886     -2.052282
  C       4.853540      2.569570      0.469025
  H       5.646836      2.406152     -0.288760
  H       4.634247      3.656028      0.485981
  H       5.260590      2.283387      1.459423
  C      -1.244230     -0.965705     -1.316765
  C      -1.298665     -2.353370     -1.098510
  C      -2.412094     -0.267761     -1.670032
  C      -2.518193     -3.037479     -1.258763
  H      -0.396350     -2.917286     -0.825014
  C      -3.621820     -0.966856     -1.822326
  C      -3.700797     -2.360575     -1.620247
  H      -2.545083     -4.127114     -1.093141
  H      -4.529126     -0.406203     -2.101424
  C      -5.016041     -3.090367     -1.734458
  H      -4.871564     -4.154579     -2.006801
  H      -5.563236     -3.072395     -0.766413
  H      -5.678726     -2.622073     -2.489174
  Cl      0.885932     -0.399198     -3.661406
  H      -2.392014      0.819165     -1.833595
I2_i-PrOH.xyz
81
Coordinates from ORCA-job I2 E -2016.129911263625
  Pd      0.494061     -0.015317     -1.369577
  N      -0.384287      0.963561      1.434167
  C      -1.692409      1.459020      1.094032
  N       1.580709      0.060428      1.332099
  C      -2.824931      0.763121      1.584080
  C      -4.092850      1.269628      1.242226
  H      -4.994334      0.752657      1.603711
  C      -4.223481      2.411702      0.440956
  H      -5.224986      2.785294      0.177682
  C      -3.084079      3.082816     -0.021159
  H      -3.196687      3.985399     -0.641109
```

C	-1.790311	2.627946	0.302271
C	-2.701189	-0.469856	2.471480
H	-1.650334	-0.821645	2.417032
C	-3.590167	-1.629924	1.998944
H	-3.375919	-1.899462	0.947517
H	-3.411527	-2.524507	2.629704
H	-4.667968	-1.379450	2.079730
C	-3.004287	-0.111298	3.938775
H	-4.057378	0.221665	4.049510
H	-2.855691	-0.995012	4.592522
H	-2.353373	0.704944	4.310175
C	-0.570645	3.406794	-0.173695
H	0.334911	2.857334	0.151261
C	-0.517748	3.495135	-1.707047
H	-1.394170	4.037979	-2.117014
H	0.396813	4.031987	-2.031347
H	-0.493479	2.479407	-2.155796
C	-0.521233	4.798689	0.480623
H	-0.518590	4.721918	1.586812
H	0.396615	5.338829	0.170088
H	-1.394406	5.416263	0.184007
C	0.469738	0.326684	0.574369
C	0.196026	1.112550	2.693596
H	-0.324063	1.619586	3.511128
C	1.438043	0.539538	2.630141
H	2.231326	0.441314	3.377114
C	2.759581	-0.567550	0.789894
C	2.856786	-1.980301	0.826176
C	4.019212	-2.557857	0.281368
H	4.135177	-3.651172	0.290271
C	5.027805	-1.760831	-0.279241
H	5.926423	-2.235112	-0.702659
C	4.896380	-0.366451	-0.311323
H	5.689402	0.245499	-0.765814
C	3.756848	0.263519	0.225685
C	1.756510	-2.832245	1.444846
H	0.805998	-2.268936	1.330434
C	2.002557	-3.029778	2.953459
H	2.065388	-2.062781	3.489735
H	1.179113	-3.619227	3.406006
H	2.952703	-3.577383	3.123840
C	1.576425	-4.183482	0.739507
H	2.430670	-4.865164	0.928832
H	0.663200	-4.686758	1.115134
H	1.477732	-4.060656	-0.358077
C	3.571389	1.772661	0.128812
H	2.807078	2.070765	0.875605
C	3.024571	2.146389	-1.259094
H	2.034129	1.665063	-1.457316
H	2.856338	3.239133	-1.343211

```
  H       3.714802      1.827247     -2.066008
  C       4.851187      2.559103      0.448102
  H       5.640505      2.392063     -0.313030
  H       4.633464      3.645862      0.463600
  H       5.261708      2.274362      1.437431
  C      -1.234953     -0.955288     -1.321017
  C      -1.280306     -2.343516     -1.103411
  C      -2.408099     -0.265031     -1.673193
  C      -2.494894     -3.036209     -1.265660
  H      -0.375153     -2.901267     -0.826429
  C      -3.612689     -0.972634     -1.828362
  C      -3.681886     -2.367328     -1.628212
  H      -2.514620     -4.125911     -1.099445
  H      -4.524076     -0.418073     -2.106430
  C      -4.991878     -3.106182     -1.743693
  H      -4.839865     -4.168851     -2.017868
  H      -5.538173     -3.093888     -0.775091
  H      -5.658470     -2.640621     -2.496630
  Cl      0.885182     -0.365628     -3.675753
  H      -2.396358      0.822870     -1.832208
I2_ethyleneglycol.xyz
81
Coordinates from ORCA-job I2 E -2016.130937726842
  Pd      0.493373     -0.003965     -1.371824
  N      -0.384609      0.965800      1.433961
  C      -1.692628      1.461969      1.094327
  N       1.579004      0.059568      1.331214
  C      -2.825247      0.765475      1.583448
  C      -4.093156      1.272169      1.241664
  H      -4.994685      0.754895      1.602645
  C      -4.223713      2.415006      0.441424
  H      -5.225198      2.788791      0.178308
  C      -3.084227      3.086831     -0.019547
  H      -3.196742      3.990253     -0.638303
  C      -1.790483      2.631799      0.303890
  C      -2.701906     -0.467970      2.470292
  H      -1.650860     -0.819354      2.417085
  C      -3.589909     -1.628252      1.996356
  H      -3.373942     -1.898030      0.945321
  H      -3.412102     -2.522673      2.627574
  H      -4.667877     -1.377915      2.075295
  C      -3.006951     -0.110115      3.937370
  H      -4.060468      0.221899      4.046943
  H      -2.858320     -0.993996      4.590875
  H      -2.357024      0.706488      4.309660
  C      -0.570876      3.411799     -0.170319
  H       0.334823      2.861973      0.153558
  C      -0.518194      3.503454     -1.703476
  H      -1.393816      4.048666     -2.112022
  H       0.397300      4.039286     -2.026843
```

H	-0.496133	2.488508	-2.154204
C	-0.521750	4.802354	0.486831
H	-0.518904	4.723137	1.592836
H	0.396003	5.343251	0.177334
H	-1.395154	5.420222	0.191492
C	0.469199	0.329854	0.573479
C	0.194969	1.110728	2.694182
H	-0.324809	1.616655	3.512628
C	1.436086	0.535823	2.630300
H	2.228668	0.434693	3.377651
C	2.756914	-0.569877	0.788617
C	2.853824	-1.982591	0.827565
C	4.015119	-2.561526	0.281629
H	4.130950	-3.654854	0.292762
C	5.022897	-1.765855	-0.282434
H	5.920665	-2.241209	-0.706489
C	4.892025	-0.371412	-0.316504
H	5.684689	0.239564	-0.772951
C	3.753733	0.259857	0.221665
C	1.754989	-2.833227	1.450580
H	0.804364	-2.269552	1.338784
C	2.005906	-3.029491	2.958588
H	2.071657	-2.062150	3.493825
H	1.183329	-3.617693	3.414302
H	2.956006	-3.578068	3.126064
C	1.572203	-4.185407	0.747635
H	2.426617	-4.867192	0.935766
H	0.659691	-4.687553	1.126493
H	1.470719	-4.064480	-0.349926
C	3.569441	1.769097	0.124368
H	2.806527	2.068313	0.872093
C	3.021159	2.143112	-1.262750
H	2.028236	1.665150	-1.458462
H	2.855294	3.236150	-1.347551
H	3.708605	1.821224	-2.070942
C	4.850525	2.554424	0.441347
H	5.638575	2.386240	-0.320841
H	4.633728	3.641356	0.456821
H	5.261972	2.269555	1.430237
C	-1.231882	-0.951271	-1.322729
C	-1.272654	-2.339761	-1.105656
C	-2.407657	-0.264742	-1.673845
C	-2.484913	-3.036550	-1.268176
H	-0.365810	-2.894537	-0.828153
C	-3.609833	-0.976397	-1.829631
C	-3.674204	-2.371516	-1.630431
H	-2.501031	-4.126326	-1.102053
H	-4.523276	-0.424796	-2.106873
C	-4.981675	-3.114761	-1.746022
H	-4.826110	-4.176623	-2.021309

```
  H      -5.527259     -3.105358     -0.776999
  H      -5.650413     -2.650588     -2.497904
  Cl      0.881035     -0.347120     -3.682201
  H      -2.399855      0.823507     -1.831152
I2_n-BuOH.xyz
81
Coordinates from ORCA-job I2 E -2016.129530910177
  Pd      0.494136     -0.019902     -1.368730
  N      -0.384245      0.962564      1.434197
  C      -1.692351      1.457900      1.093864
  N       1.581230      0.060506      1.332395
  C      -2.824921      0.762398      1.584304
  C      -4.092782      1.269012      1.242451
  H      -4.994313      0.752292      1.604168
  C      -4.223299      2.410814      0.440792
  H      -5.224766      2.784482      0.177492
  C      -3.083844      3.081496     -0.021784
  H      -3.196374      3.983782     -0.642177
  C      -1.790123      2.626498      0.301602
  C      -2.701195     -0.470387      2.471955
  H      -1.650429     -0.822391      2.417168
  C      -3.590547     -1.630337      1.999867
  H      -3.376736     -1.899907      0.948368
  H      -3.411817     -2.524935      2.630582
  H      -4.668279     -1.379699      2.081078
  C      -3.003696     -0.111506      3.939287
  H      -4.056621      0.221868      4.050346
  H      -2.855221     -0.995148      4.593163
  H      -2.352398      0.704574      4.310388
  C      -0.570339      3.404735     -0.175075
  H       0.335097      2.855294      0.150260
  C      -0.517412      3.491794     -1.708502
  H      -1.394148      4.033738     -2.118982
  H       0.396788      4.029019     -2.033195
  H      -0.492334      2.475759     -2.156472
  C      -0.520591      4.797136      0.478150
  H      -0.518064      4.721300      1.584408
  H       0.397402      5.336815      0.167243
  H      -1.393556      5.414755      0.181024
  C       0.469805      0.325221      0.574690
  C       0.196396      1.113172      2.693291
  H      -0.323763      1.620714      3.510453
  C       1.438731      0.540823      2.630000
  H       2.232303      0.443742      3.376807
  C       2.760496     -0.566775      0.790238
  C       2.858027     -1.979525      0.825497
  C       4.020858     -2.556389      0.280890
  H       4.137036     -3.649682      0.288919
  C       5.029560     -1.758706     -0.278564
  H       5.928484     -2.232456     -0.701916
```

```
  C       4.897768     -0.364363     -0.309771
  H       5.690801      0.248081     -0.763573
  C       3.757774      0.264950      0.227008
  C       1.757535     -2.832102      1.442897
  H       0.806941     -2.269081      1.327793
  C       2.002322     -3.029993      2.951658
  H       2.064396     -2.063090      3.488218
  H       1.178681     -3.619789      3.403400
  H       2.952497     -3.577318      3.122785
  C       1.578421     -4.183056      0.736821
  H       2.432854     -4.864499      0.926183
  H       0.665257     -4.686956      1.111757
  H       1.480158     -4.059602     -0.360724
  C       3.571792      1.774059      0.130550
  H       2.806623      2.071527      0.876730
  C       3.026088      2.147974     -1.257789
  H       2.036888      1.665070     -1.457627
  H       2.856641      3.240579     -1.341342
  H       3.717893      1.830397     -2.063982
  C       4.850923      2.560911      0.451471
  H       5.641128      2.394370     -0.308851
  H       4.632831      3.647601      0.466971
  H       5.260564      2.276119      1.441158
  C      -1.236301     -0.957062     -1.320433
  C      -1.283431     -2.345205     -1.102712
  C      -2.408440     -0.265319     -1.672890
  C      -2.498938     -3.036277     -1.264870
  H      -0.378937     -2.904133     -0.825945
  C      -3.613979     -0.971325     -1.827839
  C      -3.685047     -2.365865     -1.627437
  H      -2.520096     -4.125959     -1.098710
  H      -4.524582     -0.415594     -2.106131
  C      -4.996013     -3.103008     -1.742890
  H      -4.845416     -4.165809     -2.017351
  H      -5.542239     -3.090246     -0.774252
  H      -5.662052     -2.636467     -2.495709
  Cl      0.886241     -0.372296     -3.673500
  H      -2.395172      0.822462     -1.832441
I2_t-BuOH.xyz
81
Coordinates from ORCA-job I2 E -2016.129319359919
  Pd      0.494286     -0.021382     -1.368405
  N      -0.384141      0.962353      1.434259
  C      -1.692293      1.457494      1.093858
  N       1.581559      0.060812      1.332537
  C      -2.824796      0.761996      1.584437
  C      -4.092695      1.268460      1.242544
  H      -4.994179      0.751708      1.604321
  C      -4.223312      2.410107      0.440689
  H      -5.224809      2.783643      0.177329
```

C	-3.083923	3.080760	-0.022070
H	-3.196538	3.982880	-0.642686
C	-1.790164	2.625914	0.301350
C	-2.700908	-0.470714	2.472166
H	-1.650141	-0.822691	2.417186
C	-3.590304	-1.630703	2.000270
H	-3.376771	-1.900157	0.948688
H	-3.411329	-2.525340	2.630862
H	-4.668033	-1.380165	2.081825
C	-3.003147	-0.111770	3.939534
H	-4.056029	0.221678	4.050774
H	-2.854622	-0.995379	4.593444
H	-2.351768	0.704302	4.310521
C	-0.570433	3.404069	-0.175597
H	0.335023	2.854750	0.149909
C	-0.517458	3.490634	-1.709051
H	-1.394289	4.032256	-2.119754
H	0.396618	4.027986	-2.033879
H	-0.492070	2.474492	-2.156748
C	-0.520766	4.796675	0.477206
H	-0.518215	4.721218	1.583492
H	0.397160	5.336355	0.166099
H	-1.393781	5.414148	0.179928
C	0.469969	0.324943	0.574813
C	0.196575	1.113499	2.693256
H	-0.323666	1.621135	3.510300
C	1.439056	0.541462	2.630016
H	2.232716	0.444779	3.376775
C	2.760937	-0.566345	0.790491
C	2.858340	-1.979112	0.825316
C	4.021334	-2.555888	0.280999
H	4.137397	-3.649192	0.288664
C	5.030294	-1.758106	-0.277827
H	5.929333	-2.231785	-0.701007
C	4.898554	-0.363763	-0.308772
H	5.691728	0.248735	-0.762247
C	3.758394	0.265476	0.227725
C	1.757395	-2.831790	1.441780
H	0.806917	-2.268706	1.326087
C	2.001026	-3.029993	2.950681
H	2.062370	-2.063179	3.487496
H	1.177181	-3.620102	3.401648
H	2.951212	-3.577079	3.122504
C	1.578706	-4.182530	0.735196
H	2.432885	-4.864150	0.925086
H	0.665149	-4.686420	1.109190
H	1.481393	-4.058708	-0.362387
C	3.572356	1.774583	0.131248
H	2.807142	2.071997	0.877418
C	3.026652	2.148424	-1.257137

```
  H       2.037628      1.665313     -1.457066
  H       2.857175      3.241027     -1.340702
  H       3.718579      1.830938     -2.063257
  C       4.851423      2.561514      0.452216
  H       5.641610      2.395134     -0.308160
  H       4.633270      3.648192      0.467807
  H       5.261178      2.276690      1.441849
  C      -1.236687     -0.957480     -1.320123
  C      -1.284376     -2.345576     -1.102300
  C      -2.408491     -0.265298     -1.672747
  C      -2.500168     -3.036149     -1.264331
  H      -0.380058     -2.904861     -0.825717
  C      -3.614333     -0.970805     -1.827519
  C      -3.686000     -2.365275     -1.626914
  H      -2.521736     -4.125816     -1.098128
  H      -4.524674     -0.414718     -2.105947
  C      -4.997288     -3.101870     -1.742268
  H      -4.847135     -4.164852     -2.016275
  H      -5.543715     -3.088450     -0.773749
  H      -5.662968     -2.635374     -2.495435
  Cl      0.886975     -0.375128     -3.672356
  H      -2.394721      0.822413     -1.832657
I2_water.xyz
81
Coordinates from ORCA-job I2 E -2016.131465464156
  Pd      0.492814      0.002485     -1.373134
  N      -0.384788      0.967131      1.433791
  C      -1.692753      1.463680      1.094437
  N       1.578020      0.059109      1.330682
  C      -2.825419      0.766801      1.582985
  C      -4.093323      1.273588      1.241240
  H      -4.994874      0.756105      1.601888
  C      -4.223850      2.416878      0.441618
  H      -5.225328      2.790756      0.178586
  C      -3.084326      3.089139     -0.018673
  H      -3.196798      3.993044     -0.636739
  C      -1.790593      2.634035      0.304775
  C      -2.702259     -0.466945      2.469460
  H      -1.651164     -0.818235      2.416669
  C      -3.589987     -1.627226      1.994962
  H      -3.373417     -1.897115      0.944071
  H      -3.412540     -2.521604      2.626337
  H      -4.667998     -1.376869      2.073227
  C      -3.007980     -0.109480      3.936503
  H      -4.061647      0.222191      4.045686
  H      -2.859332     -0.993500      4.589809
  H      -2.358378      0.707194      4.309178
  C      -0.571024      3.414706     -0.168419
  H       0.334761      2.864751      0.154972
  C      -0.518358      3.508076     -1.701469
```

H	-1.393541	4.054514	-2.109329
H	0.397640	4.043370	-2.024308
H	-0.497435	2.493533	-2.153213
C	-0.522182	4.804572	0.490192
H	-0.519320	4.724101	1.596100
H	0.395516	5.345910	0.181306
H	-1.395715	5.422537	0.195430
C	0.468883	0.331667	0.572926
C	0.194374	1.109804	2.694446
H	-0.325218	1.615150	3.513384
C	1.434970	0.533806	2.630338
H	2.227156	0.431048	3.377898
C	2.755401	-0.571177	0.787922
C	2.852119	-1.983869	0.828347
C	4.012795	-2.563590	0.281831
H	4.128529	-3.656925	0.294191
C	5.020152	-1.768693	-0.284111
H	5.917447	-2.244661	-0.708495
C	4.889614	-0.374216	-0.319285
H	5.682104	0.236203	-0.776790
C	3.752008	0.257797	0.219493
C	1.754045	-2.833765	1.453710
H	0.803392	-2.269821	1.343449
C	2.007704	-3.029514	2.961343
H	2.075209	-2.062044	3.496095
H	1.185572	-3.616981	3.418794
H	2.957720	-3.578751	3.127112
C	1.569666	-4.186381	0.751952
H	2.424105	-4.868319	0.939374
H	0.657496	-4.687863	1.132513
H	1.466678	-4.066386	-0.345583
C	3.568420	1.767101	0.121977
H	2.806219	2.066931	0.870155
C	3.019468	2.141365	-1.264738
H	2.025151	1.665390	-1.459159
H	2.855062	3.234588	-1.349909
H	3.705388	1.817898	-2.073591
C	4.850220	2.551742	0.437824
H	5.637611	2.382950	-0.324910
H	4.633985	3.638778	0.453351
H	5.262112	2.266712	1.426475
C	-1.230284	-0.948984	-1.323631
C	-1.268374	-2.337593	-1.106739
C	-2.407573	-0.264671	-1.674222
C	-2.479260	-3.036787	-1.269365
H	-0.360535	-2.890600	-0.828931
C	-3.608329	-0.978702	-1.830302
C	-3.669879	-2.374041	-1.631531
H	-2.493262	-4.126588	-1.103211
H	-4.522958	-0.428863	-2.107151

```
  C      -4.975862     -3.119867     -1.747178
  H      -4.818190     -4.181321     -2.022821
  H      -5.521188     -3.111894     -0.778002
  H      -5.645734     -2.656712     -2.498677
  Cl      0.878064     -0.336195     -3.685918
  H      -2.402052      0.823748     -1.830697
I2_2-methyl-1-butanol.xyz
81
Coordinates from ORCA-job I2 E -2016.129096710275
  Pd      0.494325     -0.023493     -1.368003
  N      -0.384077      0.961971      1.434277
  C      -1.692250      1.456974      1.093784
  N       1.581875      0.061003      1.332679
  C      -2.824729      0.761580      1.584534
  C      -4.092632      1.268010      1.242636
  H      -4.994104      0.751307      1.604504
  C      -4.223267      2.409515      0.440595
  H      -5.224770      2.783014      0.177210
  C      -3.083898      3.080034     -0.022388
  H      -3.196535      3.981992     -0.643233
  C      -1.790132      2.625219      0.301022
  C      -2.700751     -0.471054      2.472347
  H      -1.650026     -0.823119      2.417084
  C      -3.590358     -1.630988      2.000734
  H      -3.377189     -1.900383      0.949068
  H      -3.411210     -2.525665      2.631221
  H      -4.668051     -1.380419      2.082682
  C      -3.002558     -0.111996      3.939773
  H      -4.055343      0.221663      4.051279
  H      -2.854044     -0.995589      4.593709
  H      -2.350955      0.703980      4.310586
  C      -0.570390      3.403145     -0.176271
  H       0.335034      2.853866      0.149404
  C      -0.517402      3.489109     -1.709761
  H      -1.394374      4.030315     -2.120707
  H       0.396505      4.026634     -2.034774
  H      -0.491630      2.472829     -2.157104
  C      -0.520625      4.795986      0.476026
  H      -0.518081      4.720969      1.582344
  H       0.397316      5.335519      0.164708
  H      -1.393595      5.413427      0.178552
  C       0.470061      0.324366      0.574957
  C       0.196787      1.113907      2.693114
  H      -0.323505      1.621769      3.509978
  C       1.439433      0.542220      2.629951
  H       2.233231      0.446095      3.376631
  C       2.761432     -0.565894      0.790724
  C       2.858859     -1.978670      0.825018
  C       4.022066     -2.555199      0.280933
  H       4.138127     -3.648503      0.288148
```

```
  C       5.031214     -1.757176     -0.277190
  H       5.930411     -2.230664     -0.700239
  C       4.899406     -0.362842     -0.307726
  H       5.692675      0.249830     -0.760793
  C       3.759012      0.266167      0.228529
  C       1.757610     -2.831584      1.440617
  H       0.807161     -2.268567      1.324369
  C       2.000240     -3.030007      2.949644
  H       2.061011     -2.063254      3.486644
  H       1.176208     -3.620338      3.399980
  H       2.950420     -3.576914      3.122075
  C       1.579460     -4.182154      0.733594
  H       2.433583     -4.863766      0.923775
  H       0.665740     -4.686240      1.106928
  H       1.482740     -4.057992     -0.363998
  C       3.572796      1.775264      0.132167
  H       2.807345      2.072472      0.878186
  C       3.027348      2.149100     -1.256346
  H       2.038789      1.665379     -1.456742
  H       2.857452      3.241652     -1.339753
  H       3.719774      1.832137     -2.062244
  C       4.851649      2.562361      0.453558
  H       5.642046      2.396199     -0.306648
  H       4.633355      3.649013      0.469192
  H       5.261257      2.277522      1.443250
  C      -1.237317     -0.958260     -1.319793
  C      -1.285797     -2.346312     -1.101912
  C      -2.408666     -0.265419     -1.672548
  C      -2.502002     -3.036159     -1.263858
  H      -0.381754     -2.906125     -0.825510
  C      -3.614934     -0.970208     -1.827173
  C      -3.687442     -2.364603     -1.626426
  H      -2.524193     -4.125817     -1.097681
  H      -4.524921     -0.413598     -2.105708
  C      -4.999171     -3.100425     -1.741746
  H      -4.849645     -4.163546     -2.015563
  H      -5.545722     -3.086498     -0.773302
  H      -5.664476     -2.633693     -2.495101
  Cl      0.887530     -0.378466     -3.671146
  H      -2.394199      0.822226     -1.832763
I2_IsoamylAlcohol.xyz
81
Coordinates from ORCA-job I2 E -2016.129096792696
  Pd      0.494395     -0.023793     -1.367939
  N      -0.384084      0.961890      1.434286
  C      -1.692253      1.456897      1.093785
  N       1.581892      0.060978      1.332703
  C      -2.824738      0.761546      1.584578
  C      -4.092637      1.267985      1.242677
  H      -4.994114      0.751313      1.604576
```

C	-4.223261	2.409463	0.440596
H	-5.224760	2.782971	0.177210
C	-3.083885	3.079945	-0.022422
H	-3.196514	3.981884	-0.643297
C	-1.790124	2.625117	0.300986
C	-2.700779	-0.471055	2.472441
H	-1.650049	-0.823110	2.417230
C	-3.590357	-1.631012	2.000828
H	-3.377138	-1.900441	0.949180
H	-3.411233	-2.525668	2.631353
H	-4.668056	-1.380445	2.082716
C	-3.002647	-0.111948	3.939842
H	-4.055444	0.221691	4.051299
H	-2.854138	-0.995514	4.593818
H	-2.351076	0.704057	4.310649
C	-0.570376	3.403003	-0.176359
H	0.335045	2.853755	0.149378
C	-0.517367	3.488824	-1.709858
H	-1.394364	4.029942	-2.120869
H	0.396516	4.026370	-2.034907
H	-0.491526	2.472504	-2.157105
C	-0.520620	4.795904	0.475807
H	-0.518106	4.720993	1.582132
H	0.397332	5.335401	0.164461
H	-1.393576	5.413323	0.178251
C	0.470062	0.324272	0.574985
C	0.196789	1.113888	2.693113
H	-0.323509	1.621768	3.509962
C	1.439451	0.542235	2.629958
H	2.233259	0.446158	3.376633
C	2.761469	-0.565862	0.790730
C	2.858932	-1.978635	0.824956
C	4.022150	-2.555111	0.280842
H	4.138235	-3.648412	0.288004
C	5.031277	-1.757038	-0.277248
H	5.930482	-2.230485	-0.700322
C	4.899435	-0.362707	-0.307719
H	5.692687	0.250006	-0.760761
C	3.759027	0.266250	0.228569
C	1.757706	-2.831602	1.440521
H	0.807245	-2.268603	1.324305
C	2.000353	-3.030083	2.949538
H	2.061099	-2.063349	3.486577
H	1.176340	-3.620457	3.399857
H	2.950550	-3.576969	3.121942
C	1.579581	-4.182139	0.733433
H	2.433734	-4.863732	0.923549
H	0.665892	-4.686278	1.106769
H	1.482821	-4.057917	-0.364148
C	3.572781	1.775348	0.132270

```
  H       2.807259      2.072502      0.878239
  C       3.027432      2.149242     -1.256274
  H       2.038931      1.665457     -1.456787
  H       2.857491      3.241797     -1.339622
  H       3.719962      1.832377     -2.062126
  C       4.851589      2.562457      0.453808
  H       5.642054      2.396348     -0.306338
  H       4.633272      3.649104      0.469474
  H       5.261121      2.277581      1.443522
  C      -1.237359     -0.958344     -1.319749
  C      -1.285962     -2.346384     -1.101848
  C      -2.408627     -0.265400     -1.672545
  C      -2.502225     -3.036120     -1.263803
  H      -0.381977     -2.906273     -0.825422
  C      -3.614960     -0.970079     -1.827173
  C      -3.687599     -2.364462     -1.626399
  H      -2.524521     -4.125774     -1.097612
  H      -4.524891     -0.413389     -2.105734
  C      -4.999395     -3.100166     -1.741718
  H      -4.849966     -4.163296     -2.015551
  H      -5.545938     -3.086206     -0.773269
  H      -5.664665     -2.633365     -2.495062
  Cl      0.887789     -0.379022     -3.670997
  H      -2.394059      0.822239     -1.832789
I2_2-methoxyethanol.xyz
81
Coordinates from ORCA-job I2 E -2016.129328476801
  Pd      0.494253     -0.021232     -1.368440
  N      -0.384138      0.962402      1.434245
  C      -1.692293      1.457541      1.093852
  N       1.581546      0.060828      1.332518
  C      -2.824789      0.762022      1.584418
  C      -4.092693      1.268487      1.242539
  H      -4.994172      0.751722      1.604311
  C      -4.223319      2.410149      0.440709
  H      -5.224820      2.783686      0.177362
  C      -3.083936      3.080817     -0.022045
  H      -3.196559      3.982949     -0.642643
  C      -1.790173      2.625972      0.301362
  C      -2.700887     -0.470708      2.472118
  H      -1.650125     -0.822696      2.417096
  C      -3.590313     -1.630678      2.000234
  H      -3.376824     -1.900117      0.948639
  H      -3.411321     -2.525328      2.630803
  H      -4.668037     -1.380131      2.081836
  C      -3.003072     -0.111790      3.939504
  H      -4.055944      0.221674      4.050783
  H      -2.854543     -0.995418      4.593387
  H      -2.351664      0.704259      4.310488
  C      -0.570446      3.404139     -0.175574
```

H	0.335012	2.854792	0.149879
C	-0.517509	3.490779	-1.709026
H	-1.394323	4.032466	-2.119678
H	0.396587	4.028099	-2.033851
H	-0.492188	2.474657	-2.156770
C	-0.520745	4.796709	0.477299
H	-0.518158	4.721191	1.583581
H	0.397175	5.336398	0.166191
H	-1.393766	5.414204	0.180084
C	0.469966	0.324993	0.574796
C	0.196575	1.113523	2.693246
H	-0.323658	1.621159	3.510295
C	1.439046	0.541465	2.630003
H	2.232703	0.444760	3.376763
C	2.760917	-0.566359	0.790490
C	2.858294	-1.979128	0.825330
C	4.021290	-2.555930	0.281044
H	4.137334	-3.649237	0.288724
C	5.030280	-1.758172	-0.277762
H	5.929322	-2.231872	-0.700912
C	4.898568	-0.363826	-0.308721
H	5.691765	0.248655	-0.762177
C	3.758405	0.265437	0.227742
C	1.757321	-2.831781	1.441782
H	0.806849	-2.268693	1.326036
C	2.000896	-3.029943	2.950697
H	2.062240	-2.063117	3.487489
H	1.177025	-3.620025	3.401650
H	2.951066	-3.577044	3.122564
C	1.578648	-4.182543	0.735236
H	2.432812	-4.864165	0.925186
H	0.665069	-4.686410	1.109210
H	1.481381	-4.058762	-0.362355
C	3.572383	1.774544	0.131241
H	2.807235	2.071992	0.877464
C	3.026570	2.148345	-1.257111
H	2.037511	1.665249	-1.456932
H	2.857092	3.240945	-1.340706
H	3.718419	1.830813	-2.063280
C	4.851485	2.561475	0.452066
H	5.641599	2.395068	-0.308380
H	4.633339	3.648154	0.467649
H	5.261329	2.276675	1.441669
C	-1.236659	-0.957449	-1.320139
C	-1.284275	-2.345552	-1.102339
C	-2.408506	-0.265320	-1.672727
C	-2.500035	-3.036184	-1.264366
H	-0.379927	-2.904798	-0.825775
C	-3.614313	-0.970886	-1.827502
C	-3.685906	-2.365365	-1.626924

```
  H      -2.521546     -4.125855     -1.098179
  H      -4.524688     -0.414840     -2.105902
  C      -4.997157     -3.102024     -1.742281
  H      -4.846950     -4.165007     -2.016254
  H      -5.543603     -3.088599     -0.773773
  H      -5.662846     -2.635579     -2.495472
  Cl      0.886875     -0.374884     -3.672443
  H      -2.394795      0.822398     -1.832601
I2_Anisole.xyz
81
Coordinates from ORCA-job I2 E -2016.122489689711
  Pd      0.491585     -0.068644     -1.359932
  N      -0.383094      0.952668      1.434684
  C      -1.691228      1.445674      1.091877
  N       1.588012      0.063516      1.335621
  C      -2.823940      0.753654      1.586170
  C      -4.091382      1.260078      1.243597
  H      -4.993068      0.745005      1.606989
  C      -4.221373      2.398318      0.437190
  H      -5.222610      2.771568      0.172700
  C      -3.081831      3.064830     -0.030457
  H      -3.194105      3.963031     -0.656714
  C      -1.788366      2.609839      0.293064
  C      -2.699331     -0.477124      2.476280
  H      -1.649745     -0.831677      2.415447
  C      -3.593668     -1.635578      2.010596
  H      -3.387332     -1.904414      0.957560
  H      -3.412139     -2.530890      2.639530
  H      -4.670518     -1.383757      2.099716
  C      -2.992338     -0.114889      3.944630
  H      -4.042784      0.224209      4.061377
  H      -2.845043     -0.997814      4.599786
  H      -2.335744      0.698827      4.311855
  C      -0.567913      3.381821     -0.192146
  H       0.336446      2.832904      0.137433
  C      -0.514485      3.454078     -1.726373
  H      -1.394604      3.985747     -2.142949
  H       0.395406      3.995704     -2.055725
  H      -0.479763      2.434874     -2.165941
  C      -0.515192      4.779943      0.448667
  H      -0.513546      4.715270      1.555694
  H       0.403581      5.315534      0.133007
  H      -1.386462      5.397337      0.146267
  C       0.471551      0.310716      0.577508
  C       0.200718      1.121016      2.690076
  H      -0.320830      1.633402      3.503102
  C       1.446766      0.556596      2.628497
  H       2.243338      0.472206      3.373462
  C       2.771701     -0.556619      0.795193
  C       2.871112     -1.969408      0.817964
```

C	4.038557	-2.539529	0.277244
H	4.155398	-3.632668	0.274242
C	5.050006	-1.735544	-0.267402
H	5.952112	-2.204149	-0.689467
C	4.915508	-0.341662	-0.288632
H	5.709559	0.275431	-0.734154
C	3.770474	0.281427	0.244192
C	1.765425	-2.828527	1.416895
H	0.815150	-2.267166	1.292313
C	1.991386	-3.033885	2.927439
H	2.041769	-2.069251	3.469615
H	1.164941	-3.629529	3.366322
H	2.941890	-3.577177	3.109286
C	1.597132	-4.174435	0.699119
H	2.451133	-4.855739	0.891338
H	0.681880	-4.684082	1.060983
H	1.508849	-4.041350	-0.397996
C	3.580610	1.790480	0.152115
H	2.806349	2.081628	0.891670
C	3.047952	2.168018	-1.240934
H	2.070570	1.672846	-1.458045
H	2.871456	3.260006	-1.319204
H	3.754570	1.863509	-2.039276
C	4.853380	2.579834	0.491544
H	5.652320	2.419812	-0.261025
H	4.632809	3.666060	0.509439
H	5.254646	2.292110	1.483901
C	-1.254319	-0.974384	-1.312629
C	-1.318654	-2.361235	-1.092645
C	-2.416283	-0.268605	-1.668387
C	-2.543136	-3.036345	-1.251663
H	-0.419634	-2.931286	-0.821190
C	-3.631257	-0.958728	-1.818341
C	-3.720680	-2.351236	-1.613715
H	-2.577911	-4.125757	-1.086075
H	-4.534003	-0.391874	-2.099590
C	-5.041142	-3.071687	-1.727774
H	-4.904281	-4.136771	-2.000753
H	-5.588886	-3.050353	-0.760059
H	-5.700171	-2.599085	-2.483018
Cl	0.888592	-0.439655	-3.645124
H	-2.387288	0.817128	-1.837291

I2_MeOH.xyz
81
Coordinates from ORCA-job I2 E -2016.130685730546

Pd	0.493594	-0.006857	-1.371274
N	-0.384570	0.965150	1.433989
C	-1.692597	1.461173	1.094205
N	1.579411	0.059734	1.331436
C	-2.825222	0.764897	1.583586

C	-4.093111	1.271613	1.241802
H	-4.994651	0.754474	1.602940
C	-4.223638	2.414254	0.441289
H	-5.225113	2.788049	0.178155
C	-3.084143	3.085822	-0.020012
H	-3.196640	3.989028	-0.639084
C	-1.790413	2.630762	0.303404
C	-2.701840	-0.468438	2.470569
H	-1.650876	-0.820019	2.416994
C	-3.590222	-1.628588	1.997040
H	-3.374795	-1.898314	0.945886
H	-3.412230	-2.523071	2.628118
H	-4.668121	-1.378127	2.076532
C	-3.006292	-0.110385	3.937718
H	-4.059665	0.221969	4.047631
H	-2.857727	-0.994245	4.591267
H	-2.356018	0.706058	4.309763
C	-0.570758	3.410395	-0.171292
H	0.334883	2.860542	0.152708
C	-0.518160	3.501351	-1.704495
H	-1.393933	4.046149	-2.113266
H	0.397156	4.037321	-2.028133
H	-0.495758	2.486245	-2.154817
C	-0.521355	4.801218	0.485277
H	-0.518436	4.722499	1.591320
H	0.396439	5.341868	0.175469
H	-1.394679	5.419123	0.189783
C	0.469328	0.329011	0.573688
C	0.195164	1.111077	2.694029
H	-0.324714	1.617261	3.512246
C	1.436520	0.536671	2.630270
H	2.229265	0.436268	3.377542
C	2.757592	-0.569287	0.788936
C	2.854636	-1.982008	0.827186
C	4.016243	-2.560532	0.281524
H	4.132162	-3.653853	0.292074
C	5.024194	-1.764463	-0.281653
H	5.922199	-2.239500	-0.705550
C	4.893119	-0.370040	-0.315201
H	5.685847	0.241227	-0.771142
C	3.754483	0.260840	0.222686
C	1.755464	-2.833023	1.449082
H	0.804850	-2.269468	1.336630
C	2.005153	-3.029620	2.957243
H	2.070141	-2.062369	3.492752
H	1.182369	-3.618163	3.412148
H	2.955279	-3.577930	3.125445
C	1.573398	-4.184961	0.745514
H	2.427781	-4.866706	0.933943
H	0.660710	-4.687414	1.123542

```
  H       1.472624     -4.063545     -0.352054
  C       3.569832      1.770048      0.125519
  H       2.806470      2.068925      0.872932
  C       3.022058      2.144018     -1.261845
  H       2.029809      1.665161     -1.458342
  H       2.855541      3.236978     -1.346438
  H       3.710327      1.822905     -2.069649
  C       4.850523      2.555702      0.443252
  H       5.638999      2.387828     -0.318565
  H       4.633452      3.642582      0.458709
  H       5.261611      2.270873      1.432308
  C      -1.232608     -0.952327     -1.322361
  C      -1.274582     -2.340732     -1.105034
  C      -2.407702     -0.264850     -1.673798
  C      -2.487456     -3.036462     -1.267410
  H      -0.368190     -2.896271     -0.827601
  C      -3.610515     -0.975458     -1.829360
  C      -3.676152     -2.370449     -1.629784
  H      -2.504518     -4.126202     -1.101148
  H      -4.523423     -0.423101     -2.106850
  C      -4.984290     -3.112552     -1.745244
  H      -4.829676     -4.174600     -2.020356
  H      -5.529925     -3.102493     -0.776254
  H      -5.652574     -2.647950     -2.497265
  Cl      0.882110     -0.351855     -3.680621
  H      -2.398881      0.823302     -1.831613
start.xyz
81
Coordinates from ORCA-job I2 E -2016.112919853605
  Pd      0.483839     -0.093821     -1.356497
  N      -0.380678      0.948835      1.434240
  C      -1.689224      1.438582      1.089550
  N       1.594150      0.068501      1.337136
  C      -2.820972      0.747770      1.586939
  C      -4.088722      1.252106      1.243894
  H      -4.989668      0.736663      1.608221
  C      -4.220000      2.387020      0.433627
  H      -5.221578      2.758443      0.168213
  C      -3.081489      3.051195     -0.038885
  H      -3.194928      3.945324     -0.670619
  C      -1.787581      2.598085      0.284233
  C      -2.693450     -0.481691      2.478290
  H      -1.644438     -0.836836      2.411226
  C      -3.590143     -1.640033      2.017698
  H      -3.390833     -1.906482      0.962880
  H      -3.403951     -2.536402      2.643780
  H      -4.666684     -1.389815      2.115185
  C      -2.977733     -0.117994      3.947882
  H      -4.026009      0.225461      4.070718
  H      -2.830490     -1.000382      4.603796
```

H	-2.317230	0.693985	4.312266
C	-0.567740	3.365682	-0.209513
H	0.336223	2.814911	0.118442
C	-0.518513	3.431370	-1.744206
H	-1.400220	3.959996	-2.161304
H	0.388706	3.974477	-2.078430
H	-0.480986	2.411547	-2.181746
C	-0.509304	4.765779	0.426598
H	-0.503636	4.706158	1.533934
H	0.408576	5.299506	0.105210
H	-1.380417	5.384510	0.126597
C	0.473925	0.302504	0.577886
C	0.205581	1.131251	2.686337
H	-0.317088	1.647362	3.495981
C	1.454103	0.572329	2.626046
H	2.252834	0.497625	3.369458
C	2.781045	-0.548283	0.800761
C	2.878404	-1.961202	0.812405
C	4.050708	-2.528093	0.280122
H	4.165313	-3.621261	0.267125
C	5.068671	-1.721306	-0.246947
H	5.974186	-2.187601	-0.663935
C	4.934750	-0.327770	-0.260876
H	5.732518	0.291398	-0.696575
C	3.784591	0.292526	0.263267
C	1.762227	-2.823277	1.387563
H	0.814705	-2.261571	1.245515
C	1.957690	-3.033928	2.901446
H	1.992273	-2.070997	3.448081
H	1.125017	-3.634126	3.322097
H	2.906453	-3.573403	3.102637
C	1.606814	-4.165239	0.660031
H	2.456168	-4.848987	0.864389
H	0.684937	-4.676906	1.001757
H	1.538576	-4.025251	-0.437484
C	3.593184	1.801520	0.170636
H	2.810678	2.090043	0.902843
C	3.073891	2.179064	-1.227992
H	2.105841	1.675871	-1.460500
H	2.894240	3.270707	-1.305858
H	3.792152	1.881140	-2.018265
C	4.860616	2.592725	0.525214
H	5.667638	2.436024	-0.219391
H	4.639397	3.678878	0.542378
H	5.253963	2.304479	1.520643
C	-1.269822	-0.984169	-1.309392
C	-1.340125	-2.370526	-1.090510
C	-2.428690	-0.272754	-1.661328
C	-2.569100	-3.038460	-1.240862
H	-0.440782	-2.945581	-0.831774

```
  C      -3.648495     -0.955498     -1.801294
  C      -3.744930     -2.346799     -1.594594
  H      -2.608308     -4.128145     -1.078033
  H      -4.548832     -0.383671     -2.079940
  C      -5.069960     -3.060048     -1.701667
  H      -4.940356     -4.126221     -1.974109
  H      -5.615543     -3.034478     -0.732720
  H      -5.728661     -2.586542     -2.456690
  Cl      0.870175     -0.452829     -3.626473
  H      -2.392891      0.811169     -1.838198
I2_Acetone.xyz
81
Coordinates from ORCA-job I2 E -2016.129907103520
  Pd      0.491776     -0.007657     -1.372014
  N      -0.384811      0.964439      1.433214
  C      -1.692607      1.460765      1.092957
  N       1.579277      0.059210      1.331160
  C      -2.825662      0.764958      1.581998
  C      -4.093231      1.272081      1.239745
  H      -4.995070      0.755196      1.600455
  C      -4.223094      2.414667      0.439093
  H      -5.224340      2.788740      0.175516
  C      -3.083227      3.085697     -0.021976
  H      -3.195192      3.988769     -0.641327
  C      -1.789775      2.630187      0.301825
  C      -2.702926     -0.468453      2.468959
  H      -1.652467     -0.821318      2.414004
  C      -3.593251     -1.627552      1.996581
  H      -3.379612     -1.897311      0.945086
  H      -3.415357     -2.522322      2.627282
  H      -4.670772     -1.375991      2.077705
  C      -3.005208     -0.109992      3.936459
  H      -4.057960      0.223924      4.047552
  H      -2.857257     -0.994024      4.589920
  H      -2.353469      0.705544      4.307938
  C      -0.569728      3.409450     -0.172479
  H       0.335569      2.858264      0.150222
  C      -0.517706      3.502689     -1.705546
  H      -1.393085      4.048976     -2.113173
  H       0.397902      4.038413     -2.028760
  H      -0.496364      2.488392     -2.157613
  C      -0.518938      4.799194      0.486301
  H      -0.515543      4.718846      1.592235
  H       0.399002      5.339814      0.176856
  H      -1.392025      5.418142      0.192288
  C       0.469248      0.328077      0.572996
  C       0.194685      1.110737      2.693300
  H      -0.325392      1.617097      3.511254
  C       1.436100      0.536427      2.629871
  H       2.228737      0.436252      3.377262
```

```
  C       2.758121     -0.569147      0.789359
  C       2.856092     -1.981762      0.827796
  C       4.018620     -2.559583      0.283440
  H       4.135147     -3.652835      0.294054
  C       5.026539     -1.762953     -0.278902
  H       5.925198     -2.237441     -0.702001
  C       4.894455     -0.368671     -0.312924
  H       5.687047      0.243025     -0.768515
  C       3.754914      0.261532      0.223768
  C       1.756701     -2.833459      1.448348
  H       0.806212     -2.269633      1.336378
  C       2.006201     -3.032130      2.956246
  H       2.071599     -2.065557      3.492981
  H       1.183272     -3.620978      3.410506
  H       2.956155     -3.580923      3.123853
  C       1.574537     -4.184311      0.742714
  H       2.428931     -4.866397      0.929882
  H       0.661908     -4.687430      1.120021
  H       1.473625     -4.061049     -0.354612
  C       3.569451      1.770606      0.125907
  H       2.804581      2.069090      0.871972
  C       3.023912      2.143902     -1.262542
  H       2.031822      1.665589     -1.460306
  H       2.858785      3.236992     -1.348346
  H       3.713020      1.821443     -2.069072
  C       4.849115      2.557058      0.445874
  H       5.638680      2.390291     -0.315063
  H       4.631305      3.643792      0.461715
  H       5.259317      2.271953      1.435226
  C      -1.234379     -0.952939     -1.322438
  C      -1.275995     -2.341089     -1.103747
  C      -2.409613     -0.265950     -1.674138
  C      -2.488872     -3.037093     -1.264650
  H      -0.369223     -2.896203     -0.826817
  C      -3.612444     -0.976803     -1.828051
  C      -3.677842     -2.371550     -1.626871
  H      -2.505636     -4.126698     -1.097478
  H      -4.525497     -0.424850     -2.105854
  C      -4.985992     -3.113891     -1.740796
  H      -4.831442     -4.176242     -2.014772
  H      -5.531310     -3.102732     -0.771624
  H      -5.654606     -2.650349     -2.493188
  Cl      0.874680     -0.342735     -3.682183
  H      -2.400755      0.821924     -1.833729
I2_Cyclohexane.xyz
81
Coordinates from ORCA-job I2 E -2016.113758343938
  Pd      0.484708     -0.092280     -1.356643
  N      -0.380846      0.949170      1.434308
  C      -1.689395      1.439102      1.089801
```

N	1.593734	0.068263	1.337045
C	-2.821170	0.748122	1.586959
C	-4.088932	1.252554	1.243971
H	-4.989906	0.737091	1.608235
C	-4.220183	2.387728	0.434007
H	-5.221759	2.759229	0.168668
C	-3.081634	3.052170	-0.038122
H	-3.195041	3.946615	-0.669424
C	-1.787730	2.598989	0.285030
C	-2.693788	-0.481451	2.478189
H	-1.644694	-0.836456	2.411646
C	-3.590187	-1.639865	2.017131
H	-3.390274	-1.906438	0.962443
H	-3.404295	-2.536159	2.643407
H	-4.666775	-1.389602	2.113936
C	-2.978840	-0.117934	3.947686
H	-4.027330	0.225068	4.070012
H	-2.831533	-1.000376	4.603513
H	-2.318727	0.694235	4.312329
C	-0.567879	3.366985	-0.208054
H	0.336152	2.816580	0.120306
C	-0.518131	3.432790	-1.742723
H	-1.399752	3.961403	-2.160018
H	0.389266	3.975820	-2.076590
H	-0.480633	2.412897	-2.180129
C	-0.510182	4.767114	0.428047
H	-0.504970	4.707403	1.535379
H	0.407738	5.301021	0.107078
H	-1.381353	5.385528	0.127555
C	0.473774	0.303202	0.577911
C	0.205243	1.130566	2.686654
H	-0.317350	1.646398	3.496552
C	1.453606	0.571287	2.626252
H	2.252196	0.495894	3.369770
C	2.780333	-0.548843	0.800306
C	2.877722	-1.961770	0.812758
C	4.049550	-2.528990	0.279674
H	4.164232	-3.622168	0.267388
C	5.067032	-1.722474	-0.248831
H	5.972194	-2.189007	-0.666344
C	4.933204	-0.328890	-0.263228
H	5.730706	0.290060	-0.699738
C	3.783547	0.291702	0.261740
C	1.762340	-2.823556	1.389864
H	0.814592	-2.261881	1.249165
C	1.960194	-3.033660	2.903530
H	1.995962	-2.070545	3.449749
H	1.128015	-3.633511	3.325642
H	2.909135	-3.573372	3.103257
C	1.605886	-4.165896	0.663216

```
  H       2.455596     -4.849442      0.866714
  H       0.684481     -4.677330      1.006554
  H       1.536105     -4.026556     -0.434298
  C       3.592420      1.800737      0.169344
  H       2.810803      2.089487      0.902384
  C       3.071722      2.178506     -1.228663
  H       2.102744      1.676178     -1.459664
  H       2.892554      3.270227     -1.306398
  H       3.788784      1.879976     -2.019806
  C       4.860485      2.591597      0.522444
  H       5.666611      2.434637     -0.223076
  H       4.639440      3.677778      0.539882
  H       5.254737      2.303210      1.517472
  C      -1.268477     -0.983517     -1.309602
  C      -1.338457     -2.369929     -1.090767
  C      -2.427492     -0.272374     -1.661807
  C      -2.567158     -3.038278     -1.241884
  H      -0.439198     -2.944716     -0.831079
  C      -3.646994     -0.955559     -1.802663
  C      -3.743042     -2.346964     -1.596237
  H      -2.606129     -4.127952     -1.078937
  H      -4.547445     -0.383995     -2.081497
  C      -5.067799     -3.060617     -1.703950
  H      -4.937729     -4.126737     -1.976360
  H      -5.613635     -3.035244     -0.735149
  H      -5.726389     -2.587125     -2.459075
  Cl      0.872353     -0.453426     -3.627629
  H      -2.392102      0.811707     -1.837948
I2_MIBK.xyz
81
Coordinates from ORCA-job I2 E -2016.128695997092
  Pd      0.492716     -0.030101     -1.366455
  N      -0.383386      0.961992      1.434311
  C      -1.691687      1.456709      1.093910
  N       1.582283      0.060438      1.333311
  C      -2.823968      0.761303      1.584990
  C      -4.091965      1.267816      1.243635
  H      -4.993334      0.751055      1.605674
  C      -4.222813      2.409484      0.441872
  H      -5.224389      2.783052      0.178877
  C      -3.083600      3.080058     -0.021400
  H      -3.196446      3.982034     -0.642178
  C      -1.789741      2.625107      0.301418
  C      -2.699468     -0.471273      2.472777
  H      -1.648542     -0.822713      2.417576
  C      -3.588299     -1.631674      2.000950
  H      -3.373773     -1.901681      0.949736
  H      -3.409771     -2.525910      2.632243
  H      -4.666143     -1.381263      2.081387
  C      -3.001453     -0.112320      3.940181
```

H	-4.054319	0.221064	4.051751
H	-2.852691	-0.995837	4.594166
H	-2.350058	0.703856	4.310962
C	-0.569990	3.402505	-0.176734
H	0.335374	2.854389	0.151147
C	-0.515707	3.484520	-1.710412
H	-1.394346	4.021268	-2.123621
H	0.396442	4.024712	-2.035950
H	-0.485465	2.467186	-2.155013
C	-0.521351	4.797062	0.471974
H	-0.521163	4.725101	1.578503
H	0.397334	5.335587	0.161121
H	-1.393481	5.414008	0.171053
C	0.469729	0.321812	0.575804
C	0.198660	1.117252	2.692191
H	-0.320955	1.627219	3.508172
C	1.441206	0.545298	2.629355
H	2.235703	0.451063	3.375526
C	2.762095	-0.565847	0.791193
C	2.859853	-1.978625	0.824370
C	4.022978	-2.554453	0.279452
H	4.139236	-3.647726	0.285618
C	5.031852	-1.755779	-0.278228
H	5.930942	-2.228765	-0.702051
C	4.899838	-0.361476	-0.307442
H	5.692860	0.251715	-0.760249
C	3.759421	0.266882	0.229522
C	1.759174	-2.832064	1.440277
H	0.808182	-2.270158	1.323006
C	2.001496	-3.028558	2.949622
H	2.061807	-2.061141	3.485502
H	1.177593	-3.618630	3.400538
H	2.951852	-3.574860	3.123018
C	1.582579	-4.183489	0.734653
H	2.438426	-4.863235	0.923783
H	0.670748	-4.689270	1.110251
H	1.483448	-4.060248	-0.362825
C	3.572907	1.776000	0.134011
H	2.805969	2.072376	0.878828
C	3.029779	2.150550	-1.255254
H	2.043731	1.663645	-1.459022
H	2.857161	3.242780	-1.337268
H	3.725342	1.836976	-2.059804
C	4.850874	2.563214	0.458580
H	5.643075	2.397268	-0.299797
H	4.632479	3.649861	0.473818
H	5.258285	2.278340	1.449178
C	-1.240560	-0.961576	-1.318481
C	-1.290744	-2.349967	-1.103027
C	-2.410894	-0.266535	-1.670127

```
  C      -2.507862     -3.037863     -1.266480
  H      -0.387337     -2.911420     -0.827711
  C      -3.618081     -0.969451     -1.826051
  C      -3.692359     -2.364099     -1.627986
  H      -2.531682     -4.127857     -1.102807
  H      -4.527366     -0.411279     -2.103773
  C      -5.004896     -3.098166     -1.745407
  H      -4.856537     -4.160489     -2.022964
  H      -5.551293     -3.087094     -0.776839
  H      -5.669833     -2.628222     -2.497083
  Cl      0.883649     -0.378600     -3.669995
  H      -2.394770      0.821298     -1.828792
I2_MEK.xyz
81
Coordinates from ORCA-job I2 E -2016.129653176362
  Pd      0.493927     -0.017965     -1.369141
  N      -0.384351      0.962826      1.434161
  C      -1.692400      1.458343      1.093850
  N       1.580946      0.060350      1.332269
  C      -2.825054      0.762903      1.584204
  C      -4.092869      1.269599      1.242282
  H      -4.994449      0.752929      1.603956
  C      -4.223282      2.411431      0.440651
  H      -5.224712      2.785173      0.177311
  C      -3.083754      3.082073     -0.021813
  H      -3.196190      3.984421     -0.642132
  C      -1.790076      2.626985      0.301633
  C      -2.701543     -0.469903      2.471860
  H      -1.650730     -0.821814      2.417505
  C      -3.590589     -1.629933      1.999369
  H      -3.376282     -1.899507      0.947967
  H      -3.412068     -2.524507      2.630178
  H      -4.668377     -1.379373      2.080080
  C      -3.004690     -0.111087      3.939074
  H      -4.057726      0.222077      4.049716
  H      -2.856298     -0.994709      4.592993
  H      -2.353693      0.705121      4.310421
  C      -0.570255      3.405301     -0.174806
  H       0.335163      2.855488      0.149942
  C      -0.517681      3.493440     -1.708178
  H      -1.394128      4.036317     -2.118045
  H       0.396826      4.030230     -2.032725
  H      -0.493504      2.477704     -2.156879
  C      -0.520134      4.797235      0.479387
  H      -0.517211      4.720595      1.585587
  H       0.397800      5.337048      0.168533
  H      -1.393172      5.415109      0.182999
  C       0.469736      0.325746      0.574514
  C       0.196089      1.112658      2.693437
  H      -0.324091      1.619906      3.510771
```

```
  C       1.438299      0.540046      2.630093
  H       2.231707      0.442421      3.377005
  C       2.760120     -0.567108      0.790118
  C       2.857700     -1.979838      0.825893
  C       4.020439     -2.556889      0.281264
  H       4.136649     -3.650179      0.289734
  C       5.029001     -1.759398     -0.278713
  H       5.927856     -2.233283     -0.702063
  C       4.897197     -0.365057     -0.310356
  H       5.690161      0.247246     -0.764468
  C       3.757321      0.264428      0.226471
  C       1.757446     -2.832269      1.443916
  H       0.806864     -2.269088      1.329510
  C       2.003217     -3.030370      2.952493
  H       2.065953     -2.063550      3.489120
  H       1.179728     -3.619997      3.404730
  H       2.953359     -3.577984      3.122877
  C       1.577716     -4.183165      0.737857
  H       2.432084     -4.864789      0.926854
  H       0.664583     -4.686865      1.113139
  H       1.479057     -4.059693     -0.359652
  C       3.571437      1.773535      0.129810
  H       2.806583      2.071220      0.876227
  C       3.025298      2.147313     -1.258374
  H       2.035662      1.664936     -1.457600
  H       2.856243      3.239957     -1.342176
  H       3.716518      1.829152     -2.064832
  C       4.850776      2.560320      0.450081
  H       5.640617      2.393675     -0.310595
  H       4.632745      3.647020      0.465621
  H       5.260830      2.275552      1.439601
  C      -1.235875     -0.956331     -1.320619
  C      -1.282184     -2.344490     -1.102826
  C      -2.408463     -0.265302     -1.673030
  C      -2.497263     -3.036321     -1.264988
  H      -0.377375     -2.902841     -0.825934
  C      -3.613562     -0.972051     -1.828015
  C      -3.683772     -2.366644     -1.627617
  H      -2.517752     -4.126002     -1.098735
  H      -4.524502     -0.416865     -2.106290
  C      -4.994287     -3.104586     -1.743054
  H      -4.843033     -4.167348     -2.017302
  H      -5.540579     -3.091967     -0.774452
  H      -5.660558     -2.638564     -2.495990
  Cl      0.885297     -0.368561     -3.674612
  H      -2.395879      0.822510     -1.832486
I2_Toluene.xyz
81
Coordinates from ORCA-job I2 E -2016.116125178671
  Pd      0.487940     -0.091118     -1.356091
```

N	-0.381612	0.948463	1.434957
C	-1.689991	1.439363	1.090828
N	1.592569	0.066544	1.337461
C	-2.822165	0.748373	1.587263
C	-4.089742	1.253726	1.244600
H	-4.991010	0.738556	1.608638
C	-4.220440	2.389903	0.435796
H	-5.221853	2.762180	0.170841
C	-3.081495	3.054657	-0.035145
H	-3.194427	3.950196	-0.665010
C	-1.787790	2.600583	0.287882
C	-2.695760	-0.481691	2.477984
H	-1.646744	-0.837103	2.412388
C	-3.592484	-1.639538	2.015926
H	-3.391747	-1.906351	0.961411
H	-3.407664	-2.535915	2.642396
H	-4.668958	-1.388408	2.111619
C	-2.982091	-0.118612	3.947371
H	-4.030879	0.223879	4.068699
H	-2.834988	-1.001379	4.602802
H	-2.322333	0.693555	4.312573
C	-0.567667	3.369080	-0.203727
H	0.336393	2.820076	0.126825
C	-0.515921	3.433096	-1.738425
H	-1.397833	3.959935	-2.157329
H	0.391430	3.976537	-2.071740
H	-0.477049	2.412376	-2.173887
C	-0.512091	4.770152	0.430424
H	-0.509128	4.711753	1.537828
H	0.406448	5.303716	0.110670
H	-1.382859	5.387675	0.126938
C	0.473343	0.303747	0.578557
C	0.203871	1.127168	2.688050
H	-0.318678	1.642158	3.498585
C	1.451967	0.567265	2.627470
H	2.250141	0.490277	3.371338
C	2.778709	-0.550428	0.799320
C	2.877661	-1.963279	0.814284
C	4.048201	-2.530346	0.277950
H	4.164223	-3.623432	0.267850
C	5.062952	-1.723697	-0.255879
H	5.967145	-2.190104	-0.675697
C	4.927943	-0.330139	-0.271797
H	5.723581	0.288946	-0.711576
C	3.779615	0.290244	0.256559
C	1.766018	-2.825190	1.398304
H	0.816963	-2.264416	1.262651
C	1.973034	-3.033580	2.911035
H	2.012437	-2.069916	3.455992
H	1.143293	-3.632955	3.338560

```
  H       2.923092     -3.573382      3.105152
  C       1.606776     -4.168703      0.674384
  H       2.459529     -4.850061      0.872250
  H       0.689091     -4.681454      1.025570
  H       1.528413     -4.031023     -0.422801
  C       3.587930      1.799182      0.164644
  H       2.806816      2.087691      0.898222
  C       3.066061      2.177127     -1.232753
  H       2.095853      1.675680     -1.461788
  H       2.886878      3.268826     -1.310302
  H       3.782090      1.878136     -2.024702
  C       4.856202      2.590185      0.516764
  H       5.661927      2.432888     -0.229120
  H       4.634745      3.676280      0.534113
  H       5.250549      2.301970      1.511797
  C      -1.265294     -0.982123     -1.309755
  C      -1.336779     -2.368494     -1.090404
  C      -2.423259     -0.270066     -1.664138
  C      -2.565627     -3.036082     -1.244832
  H      -0.438947     -2.943793     -0.826580
  C      -3.642871     -0.952553     -1.808399
  C      -3.740204     -2.344026     -1.602423
  H      -2.606039     -4.125616     -1.081338
  H      -4.542442     -0.380364     -2.088829
  C      -5.065146     -3.056848     -1.713177
  H      -4.935147     -4.122749     -1.986452
  H      -5.612061     -3.032223     -0.744994
  H      -5.722424     -2.581743     -2.468413
  Cl      0.881520     -0.463423     -3.628804
  H      -2.387149      0.814322     -1.838649
I2_Heptane.xyz
81
Coordinates from ORCA-job I2 E -2016.112919853605
  Pd      0.483839     -0.093821     -1.356497
  N      -0.380678      0.948835      1.434240
  C      -1.689224      1.438582      1.089550
  N       1.594150      0.068501      1.337136
  C      -2.820972      0.747770      1.586939
  C      -4.088722      1.252106      1.243894
  H      -4.989668      0.736663      1.608221
  C      -4.220000      2.387020      0.433627
  H      -5.221578      2.758443      0.168213
  C      -3.081489      3.051195     -0.038885
  H      -3.194928      3.945324     -0.670619
  C      -1.787581      2.598085      0.284233
  C      -2.693450     -0.481691      2.478290
  H      -1.644438     -0.836836      2.411226
  C      -3.590143     -1.640033      2.017698
  H      -3.390833     -1.906482      0.962880
  H      -3.403951     -2.536402      2.643780
```

H	-4.666684	-1.389815	2.115185
C	-2.977733	-0.117994	3.947882
H	-4.026009	0.225461	4.070718
H	-2.830490	-1.000382	4.603796
H	-2.317230	0.693985	4.312266
C	-0.567740	3.365682	-0.209513
H	0.336223	2.814911	0.118442
C	-0.518513	3.431370	-1.744206
H	-1.400220	3.959996	-2.161304
H	0.388706	3.974477	-2.078430
H	-0.480986	2.411547	-2.181746
C	-0.509304	4.765779	0.426598
H	-0.503636	4.706158	1.533934
H	0.408576	5.299506	0.105210
H	-1.380417	5.384510	0.126597
C	0.473925	0.302504	0.577886
C	0.205581	1.131251	2.686337
H	-0.317088	1.647362	3.495981
C	1.454103	0.572329	2.626046
H	2.252834	0.497625	3.369458
C	2.781045	-0.548283	0.800761
C	2.878404	-1.961202	0.812405
C	4.050708	-2.528093	0.280122
H	4.165313	-3.621261	0.267125
C	5.068671	-1.721306	-0.246947
H	5.974186	-2.187601	-0.663935
C	4.934750	-0.327770	-0.260876
H	5.732518	0.291398	-0.696575
C	3.784591	0.292526	0.263267
C	1.762227	-2.823277	1.387563
H	0.814705	-2.261571	1.245515
C	1.957690	-3.033928	2.901446
H	1.992273	-2.070997	3.448081
H	1.125017	-3.634126	3.322097
H	2.906453	-3.573403	3.102637
C	1.606814	-4.165239	0.660031
H	2.456168	-4.848987	0.864389
H	0.684937	-4.676906	1.001757
H	1.538576	-4.025251	-0.437484
C	3.593184	1.801520	0.170636
H	2.810678	2.090043	0.902843
C	3.073891	2.179064	-1.227992
H	2.105841	1.675871	-1.460500
H	2.894240	3.270707	-1.305858
H	3.792152	1.881140	-2.018265
C	4.860616	2.592725	0.525214
H	5.667638	2.436024	-0.219391
H	4.639397	3.678878	0.542378
H	5.253963	2.304479	1.520643
C	-1.269822	-0.984169	-1.309392

C	-1.340125	-2.370526	-1.090510
C	-2.428690	-0.272754	-1.661328
C	-2.569100	-3.038460	-1.240862
H	-0.440782	-2.945581	-0.831774
C	-3.648495	-0.955498	-1.801294
C	-3.744930	-2.346799	-1.594594
H	-2.608308	-4.128145	-1.078033
H	-4.548832	-0.383671	-2.079940
C	-5.069960	-3.060048	-1.701667
H	-4.940356	-4.126221	-1.974109
H	-5.615543	-3.034478	-0.732720
H	-5.728661	-2.586542	-2.456690
Cl	0.870175	-0.452829	-3.626473
H	-2.392891	0.811169	-1.838198

I2_EtOH.xyz
81
Coordinates from ORCA-job I2 E -2016.130273709447

Pd	0.493834	-0.011411	-1.370350
N	-0.384402	0.964311	1.434103
C	-1.692482	1.460031	1.094140
N	1.580117	0.060106	1.331799
C	-2.825046	0.763950	1.583880
C	-4.092955	1.270538	1.242052
H	-4.994461	0.753479	1.603373
C	-4.223547	2.412871	0.441129
H	-5.225041	2.786544	0.177912
C	-3.084107	3.084212	-0.020598
H	-3.196672	3.987084	-0.640139
C	-1.790353	2.629268	0.302831
C	-2.701463	-0.469182	2.471099
H	-1.650535	-0.820816	2.417135
C	-3.590062	-1.629340	1.998034
H	-3.375147	-1.898970	0.946759
H	-3.411743	-2.523858	2.628974
H	-4.667929	-1.378922	2.078105
C	-3.005313	-0.110867	3.938303
H	-4.058564	0.221742	4.048586
H	-2.856714	-0.994633	4.591977
H	-2.354775	0.705517	4.310035
C	-0.570699	3.408502	-0.172527
H	0.334902	2.858894	0.152032
C	-0.517894	3.498027	-1.705810
H	-1.394040	4.041710	-2.115263
H	0.396991	4.034527	-2.029784
H	-0.494405	2.482580	-2.155270
C	-0.521357	4.799918	0.482804
H	-0.518633	4.722273	1.588928
H	0.396465	5.340312	0.172632
H	-1.394604	5.417614	0.186653
C	0.469548	0.327759	0.574068

C	0.195656	1.111887	2.693809
H	-0.324326	1.618533	3.511660
C	1.437363	0.538221	2.630205
H	2.230403	0.438987	3.377310
C	2.758663	-0.568365	0.789453
C	2.855793	-1.981101	0.826674
C	4.017838	-2.559118	0.281480
H	4.133779	-3.652436	0.291170
C	5.026133	-1.762546	-0.280336
H	5.924467	-2.237186	-0.703969
C	4.894872	-0.368147	-0.313123
H	5.687757	0.243476	-0.768301
C	3.755757	0.262256	0.224282
C	1.756033	-2.832604	1.446864
H	0.805474	-2.269175	1.333385
C	2.003805	-3.029708	2.955263
H	2.067657	-2.062595	3.491184
H	1.180674	-3.618733	3.408922
H	2.953943	-3.577646	3.124609
C	1.575009	-4.184164	0.742345
H	2.429331	-4.865869	0.931217
H	0.662052	-4.687069	1.119122
H	1.475308	-4.061985	-0.355227
C	3.570667	1.771425	0.127236
H	2.806818	2.069909	0.874332
C	3.023357	2.145210	-1.260415
H	2.032089	1.664983	-1.457796
H	2.855877	3.238045	-1.344782
H	3.712668	1.825151	-2.067751
C	4.850884	2.557522	0.445732
H	5.639783	2.390082	-0.315745
H	4.633454	3.644335	0.461188
H	5.261710	2.272773	1.434926
C	-1.233892	-0.953906	-1.321610
C	-1.277679	-2.342229	-1.104206
C	-2.407940	-0.264917	-1.673414
C	-2.491471	-3.036322	-1.266562
H	-0.371950	-2.898967	-0.827041
C	-3.611705	-0.973907	-1.828810
C	-3.679251	-2.368751	-1.629006
H	-2.509965	-4.126052	-1.100391
H	-4.523797	-0.420355	-2.106587
C	-4.988381	-3.109107	-1.744532
H	-4.835159	-4.171472	-2.019207
H	-5.534377	-3.097913	-0.775752
H	-5.655755	-2.643944	-2.497019
Cl	0.883772	-0.359244	-3.677995
H	-2.397548	0.823108	-1.831822

I1_DMSO.xyz
81

```
Coordinates from ORCA-job I1_DMSO E  -2016.090090242406
  N   -1.04142680540058     -1.16665020251843      1.39623585473917
  C   -2.38900401713486     -0.74717286125032      1.12625032931001
  N   1.09825865175003     -1.36677794886062      1.22975115937995
  C   -3.21128535832326     -1.57880213537632      0.32865678631288
  C   -4.49748838051897     -1.10289987968706      0.00716812324617
  H   -5.16051558292062     -1.71674882076682     -0.62117279508854
  C   -4.94123493146295      0.14315669397698      0.47227989201590
  H   -5.94756902160216      0.50085679670104      0.20469537995115
  C   -4.11400390264084      0.93259573733171      1.28381057035677
  H   -4.47860801812420      1.90540052698706      1.64753066102430
  C   -2.81963854255205      0.50254288665665      1.63541629637966
  C   -2.71419202427398     -2.91671402147227     -0.20536085110351
  H   -1.79844047074271     -3.18159137834734      0.36179206162907
  C   -2.31181959308456     -2.79156667764686     -1.68550745952159
  H   -1.53939325442857     -2.00328344972662     -1.81402740748843
  H   -1.89836853941017     -3.75053739854016     -2.06083484392463
  H   -3.18784911415305     -2.52425920001500     -2.31308513807193
  C   -3.73120125470881     -4.04837685807628      0.00893944746065
  H   -4.65370107528112     -3.88825822268895     -0.58627993100947
  H   -3.29643490035843     -5.01716196416564     -0.31075658035210
  H   -4.02287010331326     -4.13598996849470      1.07513243307346
  C   -1.91112592332568      1.37792486113473      2.49128033656143
  H   -1.05171091372400      0.75439495992693      2.81257379720283
  C   -1.34242892592674      2.54340296853883      1.66309897912390
  H   -2.15307591252279      3.21726348438827      1.31564291945497
  H   -0.62957287245330      3.14345013734322      2.26516700421012
  H   -0.80849901820922      2.15914864719792      0.76796219171769
  C   -2.61195228565291      1.87553815045765      3.76498636067700
  H   -3.00888038404317      1.03232434885340      4.36602520385069
  H   -1.89756201124807      2.44234544822570      4.39615155407098
  H   -3.45715101695532      2.55510478583274      3.53065457861704
  C   0.01487472536986     -0.81481825709810      0.58794470251113
  C   -0.62988623268336     -1.91074959138533      2.50053526836715
  H   -1.33610429431295     -2.27876523039274      3.25101188417545
  C   0.73060908893086     -2.03596719672194      2.39599671892998
  H   1.46401431664716     -2.53466598479044      3.03686864623679
  C   2.45042368884297     -1.19625178810365      0.77349966694857
  C   2.98909351562778     -2.14193123139766     -0.13255739714460
  C   4.31398717813893     -1.94374213823961     -0.56699417125735
  H   4.76139178921451     -2.65544790860778     -1.27739463279658
  C   5.06621872543623     -0.85069861962602     -0.11321360250220
  H   6.10144076988893     -0.71517488345653     -0.46270370953612
  C   4.50426775865644      0.07285820190118      0.77897831206326
  H   5.10118052805812      0.93145500335004      1.12332579558745
  C   3.18094227866247     -0.07632109873575      1.23912820256845
  C   2.14804675997956     -3.28625870878951     -0.68313762760347
  H   1.24755614190937     -3.37556002944115     -0.04192594647739
  C   2.87811119648828     -4.63669259029998     -0.64175869885786
  H   3.21685530093581     -4.88260370436096      0.38509352511286
```

```
  H   2.20245758199248     -5.44634273776034     -0.98489755834769
  H   3.76730300814098     -4.64561793823017     -1.30558537515195
  C   1.66565283869085     -2.94207961842617     -2.10504835536644
  H   2.52462318993966     -2.84971064822106     -2.80233917730555
  H   0.98779018746620     -3.73087618028481     -2.49162120053934
  H   1.11227690129990     -1.97476297967360     -2.10130244153424
  C   2.57458960289956      0.94704247956896      2.19135579392951
  H   1.51605370962845      0.66547663318037      2.36179325546228
  C   2.56946519129679      2.35506256932363      1.57324990253583
  H   2.02445214718660      2.36157635467133      0.60782507423774
  H   2.06742939532843      3.07372090704106      2.25279485533204
  H   3.59935387668864      2.72627985810481      1.39128878063232
  C   3.28299269166724      0.92434042477024      3.55646929949824
  H   4.35201024234277      1.20720890989088      3.46043143262289
  H   2.80448025536276      1.64283045771050      4.25318889144600
  H   3.23701412913230     -0.08394376445412      4.01591276159839
  C   0.06118618693721      3.58891662344703     -1.64231849007200
  C   1.02208470764925      2.68606975015907     -2.10632216853098
  C   -1.31872486094949      3.34608563636682     -1.81578950396336
  C   0.60652121844138      1.51281486309590     -2.80824251027837
  H   2.09284762200480      2.89552745319800     -1.97261453358410
  C   -1.72695722166211      2.16880407692815     -2.44838664993298
  C   -0.78674594618036      1.23159748542220     -2.99085407739083
  H   1.36441216493406      0.92923084935667     -3.35633350855592
  H   -2.80296349663879      1.97510652451123     -2.58436235189018
  C   -1.26159379774273      0.17405978427605     -3.96223628395216
  H   -1.45999859074832      0.62683256987903     -4.95826874026427
  H   -0.51000625441711     -0.62681670792300     -4.09813215579566
  H   -2.20628557619058     -0.28877277231112     -3.61520717379166
  Cl  0.57290472283730      5.01254363496385     -0.74779089967159
  H   -2.05651240019853      4.06042625398976     -1.42447054979783
  Pd  -0.01063416018423      0.26371555770531     -1.11459019170926
I2_DMSO.xyz
81
Coordinates from ORCA-job I2_DMSO E  -2016.131096990583
  Pd  0.49330256346159     -0.00210758225403     -1.37223773630376
  N   -0.38475404145373      0.96600942750092      1.43388414585601
  C   -1.69271484505631      1.46236879578395      1.09427824913402
  N   1.57866267239712      0.05932632408037      1.33105632677672
  C   -2.82541793336901      0.76591231592093      1.58328453030910
  C   -4.09327700698061      1.27275581948848      1.24150618472435
  H   -4.99486163481467      0.75554412236748      1.60244471390797
  C   -4.22371659390899      2.41569529378586      0.44138685573789
  H   -5.22516410006787      2.78961048191139      0.17830455940805
  C   -3.08415268117977      3.08748825442500     -0.01945432399908
  H   -3.19656401916020      3.99102774950695     -0.63805929657803
  C   -1.79045543780775      2.63230992450131      0.30398252070866
  C   -2.70227463452546     -0.46759896800215      2.47007304724838
  H   -1.65126055616126     -0.81909467607103      2.41699800191756
  C   -3.59035676532839     -1.62778536924944      1.99602999002279
```

H	-3.37428190955981	-1.89765666232943	0.94503755849162
H	-3.41274716019607	-2.52219716216971	2.62731414248355
H	-4.66830310531862	-1.37730403741530	2.07479687304509
C	-3.00747976179220	-0.10977019427488	3.93712816637551
H	-4.06100239446836	0.22227017536223	4.04656596969766
H	-2.85895286897853	-0.99369015035232	4.59060097541805
H	-2.35755198192532	0.70678647713210	4.30951022221977
C	-0.57079242339366	3.41236175580875	-0.16999682254140
H	0.33488376795924	2.86225167628273	0.15343661376505
C	-0.51836887287400	3.50482669386952	-1.70311180237940
H	-1.39375720986448	4.05076297784421	-2.11119353964887
H	0.39738505701514	4.04028243044149	-2.02636862876672
H	-0.49704480344644	2.49008581900940	-2.15434677961062
C	-0.52134613775237	4.80255881115247	0.48787900195874
H	-0.51825160366395	4.72271456198795	1.59383630813573
H	0.39642180861388	5.34348569185196	0.17847751719812
H	-1.39474838185250	5.42067831853367	0.19305570523876
C	0.46909167811537	0.33032115287587	0.57328687063472
C	0.19463250373692	1.11017677859267	2.69427796866981
H	-0.32513953599428	1.61585562762758	3.51288609185386
C	1.43562026792089	0.53499703582127	2.63034740279868
H	2.22804993625321	0.43335167136275	3.37779379556035
C	2.75646357669332	-0.57025394636719	0.78838329372231
C	2.85344121690300	-1.98295526156035	0.82771971488691
C	4.01458491482148	-2.56200138128032	0.28155120304850
H	4.13049197648340	-3.65532182106444	0.29300922971546
C	5.02214746545080	-1.76645126374720	-0.28308565708254
H	5.91980275394530	-2.24189697940515	-0.70728165688974
C	4.89124859612777	-0.37200888155074	-0.31743624284627
H	5.68379072513730	0.23889074692708	-0.77420154360032
C	3.75312160941114	0.25936247177230	0.22097912270518
C	1.75491968053776	-2.83348209091054	1.45143049706586
H	0.80424478504955	-2.26978286776514	1.34015962532596
C	2.00672527629419	-3.02964495321808	2.95930912955916
H	2.07292854735514	-2.06228339818237	3.49444288302230
H	1.18434743751771	-3.61772267924188	3.41553935595322
H	2.95685522837845	-3.57834238716560	3.12621453116846
C	1.57170972166073	-4.18576784661270	0.74878183692928
H	2.42617197175178	-4.86756589808060	0.93663444494348
H	0.65933694984002	-4.68777864865972	1.12815609884222
H	1.46972724201701	-4.06507298198030	-0.34876328011131
C	3.56893659667325	1.76861390479512	0.12370307534459
H	2.80606008960286	2.06788937852694	0.87143188572718
C	3.02072294930874	2.14281436107582	-1.26337056469378
H	2.02744884664101	1.66541953280931	-1.45897745498242
H	2.85525744709210	3.23591120051656	-1.34818947732854
H	3.70791163933678	1.82059181493100	-2.07164903109606
C	4.85011633039857	2.55378977631084	0.44070294374758
H	5.63817057250384	2.38549050946663	-0.32145606452858
H	4.63342686463401	3.64074044546673	0.45619222644693

```
  H   5.26145481157250      2.26884172602950      1.42961300757099
  C   -1.23131196912667     -0.95065730771254     -1.32311338731093
  C   -1.27137018293426     -2.33914424532782     -1.10585785720491
  C   -2.40750260608289     -0.26477897631646     -1.67418165004830
  C   -2.48326940410253     -3.03660110288319     -1.26826046332005
  H   -0.36427271050014     -2.89340846570926     -0.82815056196519
  C   -3.60930565634205     -0.97709457468231     -1.82991404915454
  C   -3.67292698758228     -2.37223657405712     -1.63057249480164
  H   -2.49882172398903     -4.12635082249429     -1.10191000722501
  H   -4.52307160813631     -0.42600701559934     -2.10711680915953
  C   -4.98000874322435     -3.11618738824290     -1.74598440216451
  H   -4.82391244524206     -4.17791526041073     -2.02148543455581
  H   -5.52531803566642     -3.10726797187271     -0.77680407060661
  H   -5.64921344855692     -2.65222888990717     -2.49758088901494
  Cl  0.88031845697677     -0.34411649148998     -3.68332741019794
  H   -2.40032761320913      0.82349714215938     -1.83140202530481
TS1_DMSO.xyz
81
Coordinates from ORCA-job TS1_DMSO E  -2016.075201510610
  Pd  0.75267424493105      1.23613170276817     -0.81935373311518
  N   -0.73710472070552     -0.59638984646156      1.15337304884674
  C   -2.02945653398669     -0.11397141050161      0.74876097323471
  N   1.39797609487337     -0.87044403762867      1.29388680526752
  C   -2.81246297664254     -0.92060571758422     -0.11711324528003
  C   -4.05844349167019     -0.40936448098395     -0.52636101166526
  H   -4.68942990111416     -1.00046531202270     -1.20565783134020
  C   -4.50592131010811      0.84422676656739     -0.08363715362014
  H   -5.48098739754144      1.22738046857353     -0.42198697975815
  C   -3.72319459642088      1.60533238864880      0.79258986735417
  H   -4.09276980622456      2.58048752888629      1.14557369076536
  C   -2.46789373738871      1.14086009919151      1.23497082047475
  C   -2.30404219234140     -2.27070933122450     -0.61474904283638
  H   -1.66087834938785     -2.69534581376351      0.18377389602608
  C   -1.42023139882630     -2.09668049937268     -1.86335699496312
  H   -0.58170789917585     -1.39604986232855     -1.67505032824577
  H   -0.99343441583621     -3.07178693849505     -2.17686653140970
  H   -2.01194553503635     -1.68892905298845     -2.70809477163654
  C   -3.42726289709689     -3.28297161779939     -0.87508928144191
  H   -4.05770572716267     -2.98660255110276     -1.73889365089131
  H   -2.99263831894922     -4.27438638660840     -1.11398492256719
  H   -4.08830401160694     -3.39941616298883      0.00741719881731
  C   -1.64796354871140      1.96755591363530      2.21822195448874
  H   -0.72074482475756      1.40356011578888      2.44505412971082
  C   -1.21530154766276      3.30936677304898      1.60566725049613
  H   -2.08987396521429      3.93094315328288      1.32192711115375
  H   -0.60524027536126      3.88659508841886      2.33045529972967
  H   -0.59822147325295      3.13219792012996      0.69783167479263
  C   -2.40602450974178      2.16458181676334      3.54244099190856
  H   -2.68963420190338      1.19188696175326      3.99316921911756
  H   -1.77257160966682      2.71060220824038      4.27125008435064
```

H	-3.33328871110729	2.75581675859653	3.39368136185227
C	0.44681734508119	-0.15009377100529	0.60987992664020
C	-0.52542715039780	-1.55263844745369	2.14537405345057
H	-1.35216307658616	-2.01245956694502	2.69542241352096
C	0.82992097456624	-1.72759915196265	2.23409305079862
H	1.43711523000348	-2.37300532745705	2.87593519650845
C	2.81102435339979	-0.70714106848064	1.08853659938722
C	3.44238641476632	-1.46237673902576	0.07142017160932
C	4.82465408818375	-1.26752017574895	-0.11828714918505
H	5.34605681396807	-1.83332225488261	-0.90562964843242
C	5.54125350811805	-0.36149118767617	0.67594563982696
H	6.62078470331403	-0.22224317792622	0.51043261163325
C	4.88956810800791	0.36818871993702	1.68032406762141
H	5.46210094788017	1.07938547560249	2.29518459707212
C	3.50922282851268	0.20975451603225	1.91174622435825
C	2.66186996852779	-2.41351405722730	-0.82601525070705
H	1.63880749455609	-2.50420473522310	-0.40850639482111
C	3.27735108878782	-3.82118716098298	-0.85607304314522
H	3.35794964307936	-4.24881978304674	0.16381243541682
H	2.64941605715734	-4.50145831373591	-1.46683480543351
H	4.29222226031679	-3.81288252381840	-1.30451213475278
C	2.52361553925431	-1.82133128562386	-2.23990706749344
H	3.51551252189061	-1.70492306571062	-2.72458694730284
H	1.90119409521101	-2.47909179052786	-2.88089626744942
H	2.03781445171471	-0.82168796913714	-2.19310725753039
C	2.79846964160773	1.03988295709982	2.97388342392074
H	1.77555972049339	0.62967133292017	3.09488463052953
C	2.64692092425701	2.49869353013531	2.50531655971824
H	2.09712831842388	2.54415816047044	1.54088608030821
H	2.08241717533491	3.09170275116728	3.25428318306153
H	3.63842550508255	2.97726421819238	2.36332167127395
C	3.49208999265110	0.95290029523452	4.34214403111676
H	4.50979201607897	1.39390985067259	4.31350844031159
H	2.90979263212459	1.51147133185807	5.10298265252490
H	3.58478902708852	-0.09843473690783	4.68233677253701
C	-0.15865973786374	2.01088950771212	-2.64883811836351
C	-0.03492375480478	1.05246537608894	-3.68031010448561
C	-1.41948115424095	2.56109467635553	-2.32114503703559
C	-1.20124815977705	0.54688063876416	-4.27164003997626
H	0.95637356114480	0.69264677992603	-3.98981912816152
C	-2.56368948087285	2.02837262041472	-2.92855963492433
C	-2.48406378489558	1.00488350936245	-3.89961857647677
H	-1.10765324252735	-0.23404494444887	-5.04323715435256
H	-3.54965362335433	2.41909401563079	-2.63180579173305
C	-3.73116148534830	0.39453385906786	-4.48379632062590
H	-3.54281042492106	-0.05087155706567	-5.48039841740088
H	-4.10971762045548	-0.42017678807930	-3.82651948400676
H	-4.54821106416391	1.13743901266833	-4.57688261440863
Cl	1.38465061592927	3.07765038004337	-2.28694250743224
H	-1.50017426150740	3.36618242230374	-1.57763243712631
